# Are the EQ-5D-5L and EQ-HWB-9 valid instruments for measuring informal carer outcomes for use in economic evaluations?

**DOI:** 10.1007/s11136-026-04410-w

**Published:** 2026-10-15

**Authors:** Jan Faller, Valeriia Sokolova, Maja Kuharic, Fatima Al Sayah, Gang Chen, Cathrine Mihalopoulos, Brendan Mulhern, Lidia Engel

**Affiliations:** 1 Health Economics Group, School of Public Health and Preventive Medicine, Monash University, 509 St Kilda Rd, Melbourne, VIC 3004 Australia; 2 Department of Medical Social Sciences, Northwestern University Feinberg School of Medicine, Chicago, IL USA; 3 Alberta PROMs and EQ-5D Research and Support Unit (APERSU), School of Public Health, University of Alberta, Edmonton, AB Canada; 4 Cancer Health Services Research, University of Melbourne, Melbourne, VIC Australia; 5 Centre for Health Economics, Monash Business School, Monash University, Melbourne, Australia; 6 Centre for Health Economic Research and Evaluation, University of Technology Sydney, Sydney, Australia

**Keywords:** Informal carer, EQ-5D, EQ-HWB, Health-related quality of life, Wellbeing, Psychometrics

## Abstract

**Objectives:**

Despite growing interest in incorporating informal carer outcomes into economic evaluations, evidence on the performance of utility measures for informal carers remains limited. This study assesses the psychometric performance of the EQ‑5D‑5L and EQ‑HWB‑9 in capturing informal carer outcomes for economic evaluations.

**Methods:**

This study utilized cross-sectional EQ-DAPHNIE survey data from English‑speaking countries (Australia, Canada, New Zealand, UK, and US) to evaluate the EQ‑HWB‑9 (UK pilot value set) and EQ‑5D‑5L (country‑specific value sets), assessing distributional (i.e., acceptability, distribution, floor and ceiling effects) and construct validity (i.e., convergent and known-group) properties, with subgroup analyses by age and financial situation.

**Results:**

There were 6,743 informal carers across Australia (*n* = 1447), Canada (*n* = 1265), New Zealand (*n* = 1435), UK (*n* = 1337), and US (*n* = 1259). The EQ-5D-5L showed a ceiling effect (19%), but not the EQ-HWB-9 (5%). Most respondents reported problems (level 2–5) on the EQ-5D-5L pain/discomfort (67%) and anxiety/depression (60%), with anxiety/depression showing the highest proportion with worst (level 5) responses (5%). For the EQ-HWB-9, ≥ 50% reported problems (level 2–5) across all domains except mobility, with ≥ 5% reporting the worst (level 5) responses in exhaustion, loneliness, cognition, sad/depressed, anxious, and control. Both instruments moderately correlated with the WHO-5, PROMIS-10 (physical and mental t-scores), ASCOT-SCT4, OPQOL-brief, PHQ-2, and GAD-2, and demonstrated known-group validity across groups relating to self-reported physical and mental disability, chronic conditions, sleep, social support, impact of caring, psychological wellbeing, and self-reported quality-of-life. Results were consistent in subgroup analyses.

**Conclusion:**

This study provides evidence supporting the acceptability and construct validity of the EQ-5D-5L and EQ-HWB-9 in measuring informal carer outcomes for use in economic evaluations. Responsiveness evidence remains limited and warrants further research.

**Supplementary Information:**

The online version contains supplementary material available at https://doi.org/10.1007/s11136-026-04410-w.

## Introduction

Informal carers refer to family members, friends, or neighbors who provide care to individuals who are ill, disabled, or elderly [1, 2]. The demand for informal carers is expected to grow significantly over the following decades. This is because caring for an ageing population has been flagged as a significant global issue, with the United Nations estimating that people aged 65 and over will outnumber people under 18 years by 2080, increasing demand for informal care [3]. In addition, there is a move to de-hospitalize health care by shifting it to low-cost home-based care, which requires additional support from informal carers [4]. These issues highlight the vital role informal carers play in our societies.

Many informal carers find fulfilment from their roles [5, 6]; however, informal care is also associated with burden including: financial, emotional, physical, psychological, and social strain [7–9]. While carer-specific measures exist (e.g., CarerQol, ASCOT-Carer, and Carer Experience Scale), they are limited in capturing the health-related effects associated with caregiving [10]. Therefore, it is important to have validated outcomes and measures for economic evaluations that can be used for informal carers, and to be able to quantify the impact of the caring situation on their health and wellbeing. Having appropriate outcome measures for use in economic evaluations can assist in assessing and evaluating the impact of treatments and services, and promoting policies that recognize and value informal carer contribution to the wider society [11].

Using preference-based measures of health-related quality of life (HRQoL) is especially appealing to health technology assessment (HTA) bodies, which provide consistency and enable comparisons across submissions [12, 13]. Preference-weighted scores or value sets can be attached to HRQoL measure responses and used to calculate quality-adjusted life years (QALYs). Value sets are based on societal weighting of health states described by the HRQoL measures, where more desirable health states identified by society are assigned a higher values [14]. These value sets differ across countries and type of valuation techniques used to derive preferences [15].

A recent systematic review found limited, but growing evidence around the validity of preference-based measures for use in informal carers [16]. The EQ-5D is a generic preference-based HRQoL measure, which can be used in different population groups and disease areas [17]. As a generic preference-based measure, it primarily focuses on health status and HRQoL through dimensions relating to mobility, self-care, usual activities, pain/discomfort, and anxiety/depression. It can be further described as a measure to report individual HRQoL, with corresponding preference-weighted scores suitable for use in economic evaluations. It is widely used internationally for economic evaluations, with some HTA bodies preferencing it over other measures [18]. The EQ-5D has been previously used in studies examining informal carer HRQoL outcomes, with studies showing it as a psychometrically sound tool to measure informal carer health outcomes [10, 19, 20]. Compared with carer-specific measures (e.g., ASCOT-Carer, Carer Experience Scale and CarerQol-7D), studies have reported that the EQ-5D appeared sensitive to constructs associated with quality of life (QoL) of informal carers, but was not strongly correlated with care-related measures [10, 21], suggesting that it is sensitive to health-related changes of QoL but has limited sensitivity to care-related changes.

The EQ Health and Wellbeing (EQ-HWB) is a new 25-item generic measure developed to capture broader aspects of health and wellbeing for use in evaluating interventions across health, public health, and social care [22]. A shorter 9-item version (EQ-HWB-9) is available for which preference-weighted scores have been developed to generate QALYs [23]. The EQ-HWB-9 extends beyond traditional health status domains to include social, relational, emotional, cognitive, and autonomy-related aspects of life. Hence, the EQ-HWB-9 can be described as a broader multidimensional measure of health and wellbeing, with corresponding preference-weighted scores suitable for contexts, populations, and settings where both wellbeing and health are relevant for economic evaluations. Although the EQ-HWB was designed to enable the inclusion of impacts to informal carers, there is still limited evidence about its performance amongst informal carers, with only three studies published that have exclusively focused on carers [24–26]. Given that the EQ-5D-5L and EQ-HWB-9 capture distinct, though overlapping, constructs, they should not be considered interchangeable measures. It is, therefore, important to evaluate the validity of both instruments for assessing informal carer outcomes, recognizing that health status, HRQoL, and wellbeing are related but conceptually distinct constructs [27].

To date, only two studies evaluated the EQ-5D-5L and EQ-HWB-9 together [16], one being a population-based study evaluating whether the measures can differentiate informal carers and from non-carers [28], the other evaluated their performance in informal carers of adult care recipients [26]. Existing evidence suggests mixed findings around known-group and convergent validity of the EQ-5D-5L and EQ-HWB-9, reinforcing the need to further evaluate the performance of both measures in terms of these properties [16].

To address these gaps, this study aims to examine the psychometric performance focusing on the distributional (i.e., acceptability, distribution, floor and ceiling effects) and construct validity (i.e., convergent validity, and known-group validity) properties of the EQ-5D-5L and EQ-HWB-9 in measuring informal carer HRQoL and health and wellbeing outcomes, respectively, for use in economic evaluations across multiple countries using population-based data from the EQ-DAPHNIE (EuroQol’s Data for the Assessment of Population Health Needs and Instrument Evaluation) project.

## Methodology

This study is a secondary data analysis of the data obtained from the EQ-DAPHNIE survey [29], a EuroQol Group initiative designed to develop a research infrastructure and create comprehensive nationally representative datasets across multiple countries. Following the completion of a pilot study in 2023 in the United Kingdom (UK), data collection has been completed in five countries in 2024 (UK, Canada, United States [US], New Zealand, Australia), with ongoing data collection in other countries by the time this study was conducted.

The survey was organized into four sections: (1) Social Determinants of Health; (2) Overall Health and Wellbeing; (3) Health Behaviors and Habits; (4) Health Services and Coverage.

This study limits its analysis to the five English-speaking countries: Australia, Canada, New Zealand, UK, and the US to mitigate the potential impact of translation bias, which can arise from linguistic differences and variations in cultural interpretations. The focus on English-speaking populations was to maintain consistency in the wording and meaning of the survey instruments, ensuring that the data collected is directly comparable and not influenced by discrepancies introduced through translation.

Data collection was done via an online survey utilizing online research panels provided by Dynata. Respondents were recruited using quota sampling based on age, sex, income and area of residence (rural or urban). Survey participation was open to any panelist who was at least 18 years old and a resident of their country. Further details of the EQ-DAPHNIE methodology and the design and implementation of data quality controls are reported in Johnson et al. (2025) [29] and Al Sayah et al. (2025) [30]. Ethics approval was obtained from Monash University (Project ID: 46777) for this analysis.

### Study population

Informal carers were identified based on an affirmative response to the following question in the survey: “*Have you personally provided help or support to anyone in the last three months because they have long-term physical or mental ill-health*,* a disability or problems relating to old age? Do not include help given in a professional capacity or as part of a job*,* but include help or support given to your family*,* friends or neighbors.”*

### Measures

The Overall Health and Wellbeing section of the survey included a list of standardized measures. Aside from the EQ-5D-5L and the EQ-HWB-9 measures, the survey included other measures which were included in this study to assist with convergent and known-group validity testing. The other measures included measures of general physical and mental HRQoL (PROMIS-10), social care-related QoL (ASCOT-SCT4), QoL in the older age (OPQOL-brief), psychological wellbeing (WHO-5), and mental health (PHQ-2 and GAD-2). These measures were considered relevant given that the informal carer role is associated with emotional, physical, psychological burden. The EQ-5D-5L was always shown first in the survey, while other measures were randomized to avoid ordering bias.

#### EQ-5D-5L

The EQ-5D-5L assesses five dimensions of HRQoL: mobility, self-care, usual activities, pain/discomfort, and anxiety/depression [31, 32]. Each dimension is comprised of a single item measured in a five-point, 1 (no problems) to 5 (severe), severity scale [31, 32]. We used country-specific value sets to transform the responses into EQ-5D-5L utility values [33–37]. Higher values indicate better HRQoL.

#### EuroQol Health and Wellbeing instrument (EQ-HWB)

The EQ-HWB assesses seven health and wellbeing domains: activity, autonomy, cognition, feelings and emotions, physical sensations, relationships, and self-identity [22]. Each item is rated using a 5-point scale ranging from 1 (absence of issues) to 5 (most severe level of response). A sum score for the 25-item EQ-HWB was calculated, generating a score ranging from 25 (best possible health and wellbeing) to 125 (worst possible health and wellbeing) [22]. The EQ-HWB was included in the New Zealand and UK surveys.

The EQ-HWB-9 comprises nine items from the EQ-HWB. The EQ-HWB-9 can be used either as a stand-alone measure or derived from the 25-item EQ-HWB responses. Each of the 9-items denotes a dimension: activities, mobility, pain, exhaustion, loneliness, cognition, sad/depressed, anxious, and control. The EQ-HWB-9 was scored using the UK pilot value set to calculate utility values [23]. Higher values indicate better health and wellbeing. The EQ-HWB-9 was included in the Australia, Canada, and US surveys. However, EQ-HWB responses were used to derive the EQ-HWB-9 in New Zealand and UK.

#### World Health Organization Well-Being Index (WHO-5)

The WHO-5 is a five-item self-report measure assessing psychological wellbeing [38]. Each item is scored using a 6-point response (0 to 5) scale, with higher scores indicating better psychological wellbeing. A raw score can be calculated, ranging from 0 (poorest state) to 25 (best state) [38, 39]. The WHO-5 was only included in the Australian, Canadian, and US surveys.

#### Patient Reported Outcomes Measurement Information System Global Health Short Form (PROMIS-10)

The PROMIS-10 is a 10-item patient reported outcome measure assessing physical and mental health domains [40]. The PROMIS-10 physical and mental health components are scored using a T-score metric for the US general population. Higher T-scores reflect better overall physical and mental health [40].

#### Adult Social Care Outcomes Toolkit (ASCOT-SCT4)

The ASCOT-SCT4 is a multi-dimensional preference-weighted measure assessing social care-related QoL (SCRQoL) [41–43]. It comprises eight attributes: control over daily life, personal cleanliness and comfort, food and drink, personal safety, social participation and involvement, occupation, accommodation cleanliness and comfort, and dignity. Responses to each attribute range from 1 (ideal) to 4 (having high-level needs). Responses to the ASCOT-SCT4 were converted into current SCRQoL values using a scoring algorithm anchored on a 0 (dead) to 1 (ideal state) scale [42].

#### Older People’s Quality of Life questionnaire (OPQOL-brief)

The OPQOL-brief is a 13-item measure assessing QoL in the older age [44]. Each item is scored using a 1 to 5 scale, with the summary scores ranging from 13 to 65. Higher scores reflect a higher QoL [–44]. This measure was only completed by respondents aged 65 years and older.

#### Patient Health Questionnaire (PHQ-2)

The PHQ-2 is a brief screening tool for depression [45]. The two items, which are around the frequency of depressed mood and anhedonia, are scored on a 4-point scale (0 = not at all; 3 = nearly every day). Total scores range from 0 to 6, with higher scores indicating a higher frequency of depressive symptoms [45].

#### Generalized Anxiety Disorder (GAD-2)

The GAD-2 is a brief screening tool for anxiety symptoms [46]. The two items inquire about the frequency of feeling anxious and worried. Each item is scored on a 4-point scale (0 = not at all; 3 = nearly every day). Total scores range from 0 to 6, with higher scores indicating a higher frequency of anxiety symptoms [46].

### Statistical analysis

Demographic characteristics were reported for the total sample and by country. Descriptive statistics for demographics were reported as means with standard deviations or population with percentages. All five country-specific EQ-5D-5L value sets were applied and reported for the total informal carer sample as well as for informal carers for each country. Primary analyses were conducted on the combined dataset (i.e., applying each value set to participants from all five countries) and for each country dataset (i.e., country-specific value set). All analyses, including formulation of a priori hypotheses, were pre-planned. Data analyses were conducted in STATA SE version 18 [47]. Missing data were excluded from the analysis.

The following psychometric properties were explored: acceptability, distributional properties, convergent validity, and known-group validity.

#### Acceptability

Acceptability was assessed by examining missing responses on the item level of each instrument and overall. Skipping responses was possible in the survey, except in the UK pilot survey that had two versions where one included mandatory responses and the other allowed skipping. Any item with ≥ 5% missing values was flagged.

#### Distributional properties

Distributional properties were explored descriptively for each measure and distribution was further assessed using bar graphs. In addition, floor and ceiling effects, defined as the percentage with the lowest and highest possible score, were used to determine the distribution properties of the measures. Floor and ceiling effects were examined at the instrument level using a 15% threshold [48]. This threshold was chosen as it is commonly used in the psychometric literature.

#### Convergent validity

Convergent validity was examined by evaluating whether the EQ-5D-5 L and EQ-HWB-9 correlate with similar concepts based on a priori hypotheses (Online Appendix 1) using Spearman’s rank correlations. We hypothesized that items/dimensions that assess similar constructs should have moderate-strong correlation (evidence of convergent validity), and those that do not assess similar constructs should have a weak correlation (evidence of divergent validity). Correlations were assessed in accordance with Cohen’s criteria, which classify correlations as follows: strong (≥ 0.51), moderate (0.31–0.50), weak (0.11–0.30), and none (0–0.10) [49]. In addition, item-level correlation was also explored for items expected to have moderate-to-strong correlations. Item-level a priori hypotheses were formulated by two researchers (JF and LE).

#### Known-group validity

Known-group validity, or the extent to which instrument scores differ between groups that were expected to differ, was examined by investigating a priori hypotheses (Online Appendix 2). The non-parametric Mann-Whitney (two groups) or Kruskal-Wallis (more than two groups) test was used to see if there was a statistically significant difference between known groups. Differences between groups were assessed using Cohen’s d effect size (≥ 0.2 small, ≥ 0.5 moderate, ≥ 0.8 large) [50]. Confidence intervals were obtained from bootstrapping.

#### Subgroup analyses

Subgroup analyses were conducted by repeating analyses for subgroups defined by age (i.e., 60 years and older and under 60 years old); and financial comfort (i.e., coping financially, based on responses being able to live comfortably or coping with the present income; and financially strained, based on survey responses finding it difficult or very difficult with the present income).

## Results

There were 6743 informal carer respondents across Australia (*n* = 1447), Canada (*n* = 1265), New Zealand (*n* = 1435), United Kingdom (*n* = 1337), and the United States (*n* = 1259) (Table 1). Over half of the informal carers were women (56%), around 97% completed at least secondary education, and half were currently employed fulltime or part-time (50%). Demographic characteristics of subgroups are presented in Online Appendix 3.

**Table 1:** Demographic characteristics

	Australia	Canada	New Zealand	United Kingdom	United States	Total
N (%)	1447 (21.5%)	1265 (18.8%)	1435 (21.3%)	1337 (19.8%)	1259 (18.7%)	6743 (100%)
Sex at birth—N (%)						
Male	605 (42.3%)	486 (39.2%)	548 (38.7%)	601 (45.3%)	617 (49.5%)	2857 (42.9%)
Female	824 (57.7%)	754 (60.8%)	869 (61.3%)	725 (54.7%)	630 (50.5%)	3802 (57.1%)
Age—mean (SD)	49 (16.6)	50.5 (16.7)	46.2 (16.8)	46.4 (16.4)	46.8 (16.2)	47.8 (16.6)
Gender—N (%)						
Man	614 (42.5%)	493 (39.1%)	568 (39.7%)	604 (45.2%)	620 (49.4%)	2899 (43.1%)
Woman	822 (56.8%)	750 (59.4%)	851 (59.6%)	723 (54.2%)	617 (49.1%)	3763 (55.9%)
Non-binary	7 (0.5%)	15 (1.2%)	5 (0.3%)	6 (0.4%)	9 (0.7%)	42 (0.6%)
Transgender Man	0 (0%)	3 (0.2%)	1 (0.1%)	2 (0.1%)	6 (0.5%)	12 (0.2%)
Transgender Woman	0 (0%)	1 (0.1%)	0 (0%)	0 (0%)	2 (0.2%)	3 (0%)
other	3 (0.2%)	0 (0%)	4 (0.3%)	0 (0%)	2 (0.2%)	9 (0.1%)
Highest level of education—N (%)						
No formal schooling	8 (0.6%)	7 (0.6%)	15 (1.0%)	15 (1.1%)	4 (0.3%)	49 (0.7%)
Completed primary school (grades 1–6)	20 (1.4%)	45 (3.6%)	53 (3.7%)	18 (1.4%)	16 (1.3%)	152 (2.3%)
Completed secondary school (grades 7–12)	461 (32.1%)	352 (27.9%)	284 (19.9%)	487 (36.6%)	411 (32.9%)	1995 (29.7%)
Completed polytechnic school	271 (18.9%)	397 (31.5%)	377 (26.4%)	210 (15.8%)	203 (16.2%)	1458 (21.7%)
Completed Bachelor's degree or equivalent	438 (30.5%)	330 (26.2%)	548 (38.3%)	400 (30.1%)	372 (29.7%)	2088 (31.1%)
Completed Master's degree or equivalent	205 (14.3%)	111 (8.8%)	116 (8.1%)	167 (12.6%)	184 (14.7%)	783 (11.7%)
Completed Doctorate degree or equivalent	32 (2.2%)	19 (1.5%)	36 (2.5%)	33 (2.5%)	61 (4.9%)	181 (2.7%)
Current employment—N (%)						
Stay-at-home parent	71 (4.9%)	26 (2.1%)	121 (8.5%)	54 (4.1%)	46 (3.7%)	318 (4.7%)
Self employed	85 (5.9%)	100 (7.9%)	90 (6.3%)	86 (6.5%)	132 (10.5%)	493 (7.3%)
Employed full-time	574 (39.8%)	343 (27.2%)	633 (44.4%)	400 (30%)	529 (42.1%)	2,479 (36.9%)
Employed part-time	214 (14.8%)	153 (12.1%)	166 (11.6%)	251 (18.8%)	125 (10%)	909 (13.5%)
Unemployed	88 (6.1%)	119 (9.4%)	77 (5.4%)	87 (6.5%)	82 (6.5%)	453 (6.7%)
Retired	281 (19.5%)	318 (25.2%)	212 (14.9%)	235 (17.6%)	206 (16.4%)	1,252 (18.6%)
Student	24 (1.7%)	60 (4.8%)	30 (2.1%)	69 (5.2%)	49 (3.9%)	232 (3.5%)
Unable to work due to disability or illness	71 (4.9%)	122 (9.7%)	71 (5.0%)	107 (8.0%)	75 (6.0%)	446 (6.6%)
On maternity/paternity leave	4 (0.3%)	4 (0.3%)	10 (0.7%)	5 (0.4%)	1 (0.1%)	24 (0.4%)
other	32 (2.2%)	15 (1.2%)	15 (1.1%)	39 (2.9%)	11 (0.9%)	112 (1.7%)
Health and wellbeing measures—mean (sd)						
EQ-5D-5L index AU, country specific	0.845 (0.2)	0.820 (0.2)	0.862 (0.2)	0.839 (0.2)	0.810 (0.3)	0.836 (0.2)
EQ-5D-5L index CA, country specific	0.791 (0.2)	0.768 (0.2)	0.808 (0.2)	0.790 (0.2)	0.762 (0.2)	0.785 (0.2)
EQ-5D-5L index NZ, country specific	0.762 (0.2)	0.730 (0.3)	0.786 (0.3)	0.763 (0.3)	0.723 (0.3)	0.754 (0.3)
EQ-5D-5L index UK, country specific	0.798 (0.2)	0.771 (0.2)	0.815 (0.2)	0.799 (0.2)	0.767 (0.2)	0.791 (0.2)
EQ-5D-5L index US, country specific	0.762 (0.2)	0.728 (0.3)	0.782 (0.3)	0.762 (0.3)	0.725 (0.3)	0.753 (0.3)
EQ VAS	70.3 (20.6)	68.6 (21.3)	73.5 (20.3)	68.5 (22.8)	71.8 (20.5)	70.6 (21.2)
EQ-HWB score	na	na	96.3 (17.5)	96 (18.9)	na	96.2 (18.2)
EQ-HWB-9 index UK	0.702 (0.3)	0.698 (0.2)	0.736 (0.2)	0.722 (0.3)	0.674 (0.3)	0.707 (0.3)
WHO-5 global score	12.6 (6.3)	12.1 (6.2)	na	na	13.1 (6.4)	12.6 (6.3)
Promis-10 t-score physical	45.6 (8.9)	45.2 (8.5)	45.9 (8.5)	46.3 (9.6)	46.1 (8.6)	45.8 (8.8)
Promis-10 t-score mental	45.7 (9.8)	44.8 (9.8)	47.2 (9.9)	44.9 (10.6)	46.3 (10.0)	45.8 (10.0)
ASCOT social care-related quality of life	0.78 (0.2)	na	0.80 (0.2)	0.75 (0.2)	na	0.77 (0.2)
OPQOL score	54.3 (7.3)	54.5 (7.6)	55.6 (7.1)	54.2 (7.6)	55.4 (7.9)	54.8 (7.5)
PHQ-2 score	1.8 (1.7)	1.9 (1.7)	1.6 (1.6)	1.9 (1.8)	1.9 (1.8)	1.8 (1.7)
GAD-2 score	2.0 (1.8)	2.0 (1.8)	1.7 (1.7)	2.0 (1.9)	2.1 (1.9)	1.9 (1.8)
Survey response duration in minutes—median (IQR)	19 (14–26)	19 (14–27)	23 (17–33)	19 (15–27)	18 (13–27)	20 (15–28)

na, instrument not administered; IQR, interquartile range

### Acceptability

There were no issues around acceptability for both the EQ-5D-5L and EQ-HWB-9, with the largest proportion of missing values at 1% (Online Appendix 4). The EQ-5D-5L usual activities item and EQ-HWB-9 items pain, loneliness, cognition, sad/depressed, and anxiety had the highest proportion of missing responses. Similarly, no acceptability concerns (≤ 1% missing in any item) were identified during subgroup analysis (Online Appendix 5).

### Distribution properties

**Fig. 1:**
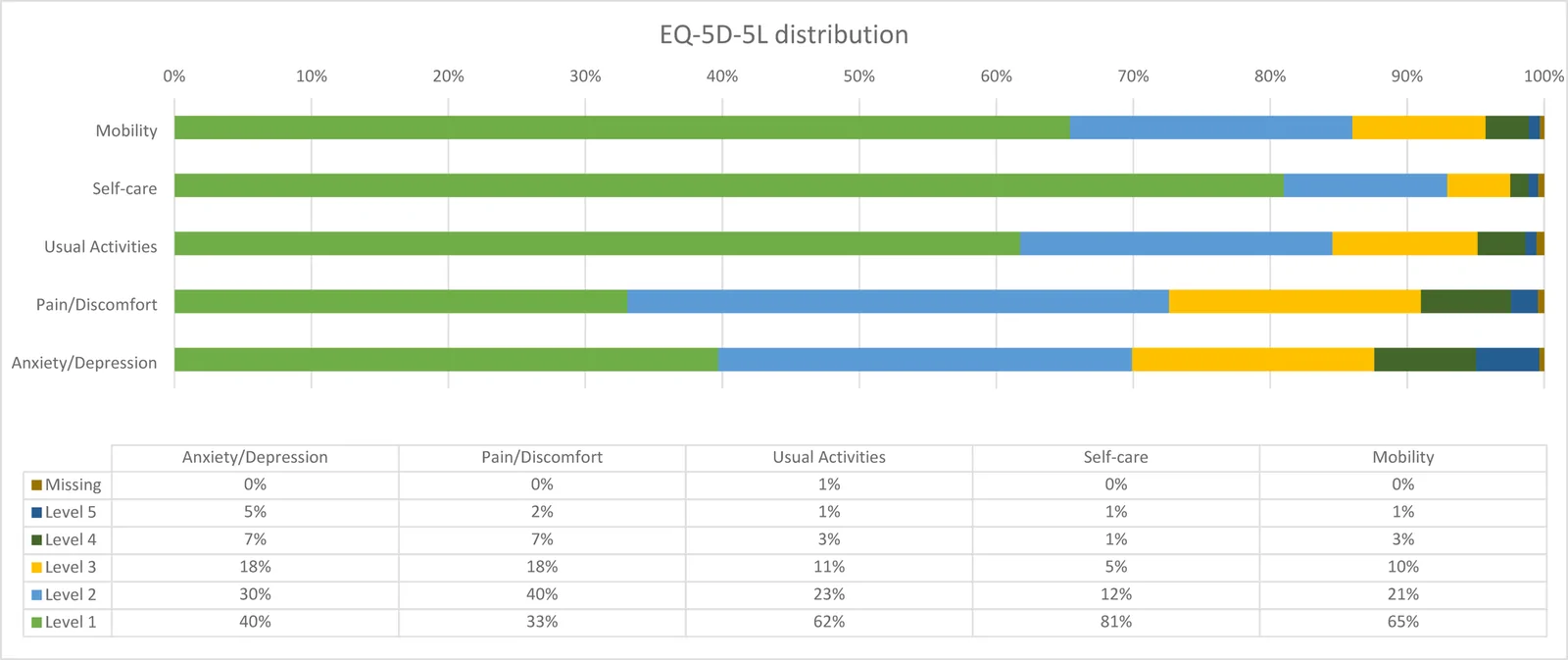
Response distribution to the EQ-5D-5 L using data from all countries

**Fig. 2:**
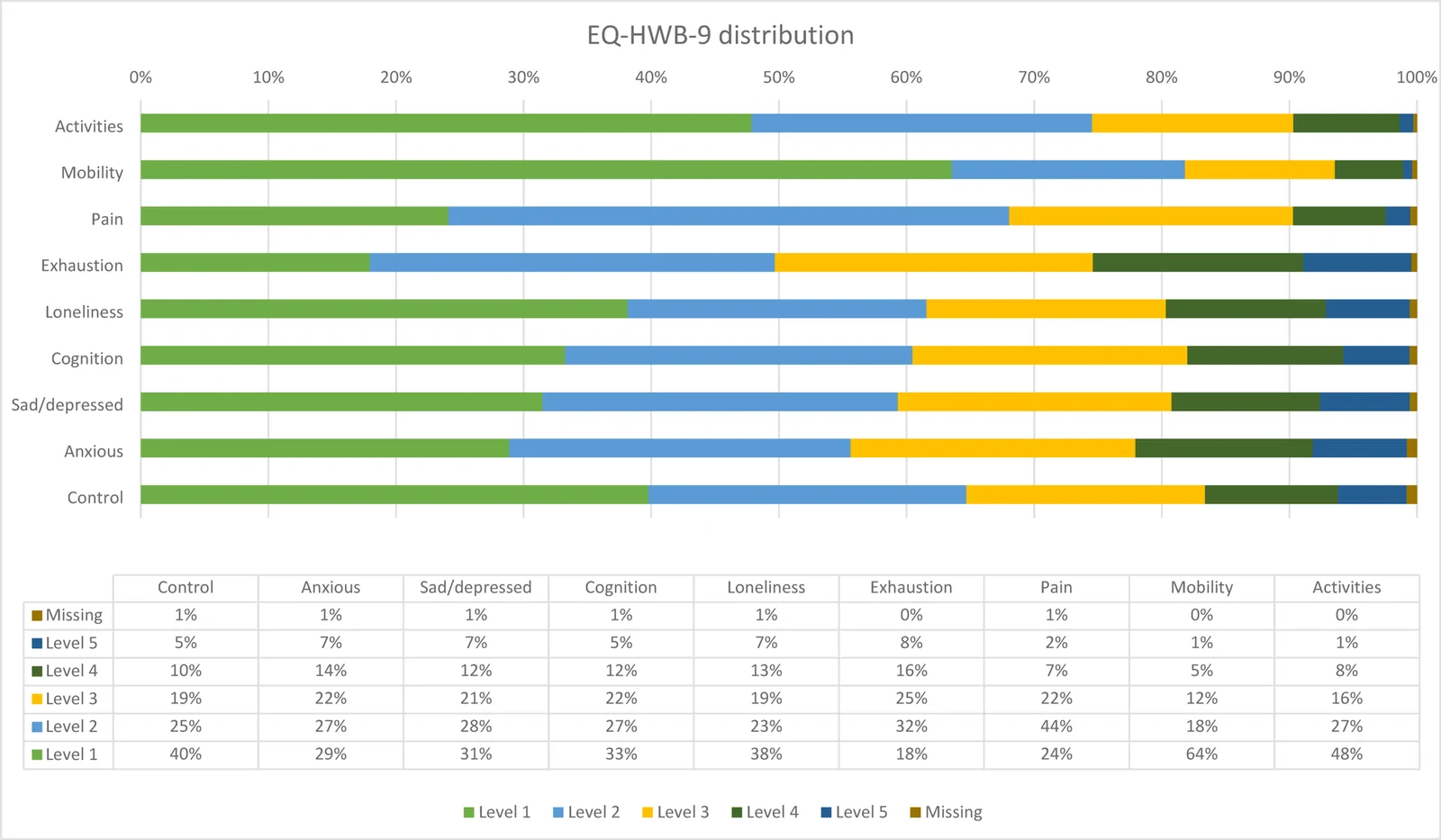
Response distribution to the EQ-HWB-9 using data from all countries

All EQ-5D-5L and EQ-HWB-9 item responses ranged from having ‘no problems’ (level 1) to ‘severe problems’ (level 5) (Figs. 1 and 2). At least half of the respondents reported no issues in the EQ-5D-5L mobility, self-care and usual activities domains. At least 60% reported having concerns (levels 2–5) for pain/discomfort (67%) and anxiety/depression (60%) domains. The anxiety/depression domain showed the highest number with the worst response (5%).

**Table 2:** Ceiling and floor effects

		Australia	Canada	New Zealand*	United Kingdom*	United States	Total
		1447	1265	1435	1337	1259	6743
EQ-5D-5L	Proportion reporting full health	279 (19%)	165 (13%)	324 (23%)	308 (23%)	191 (15%)	1267 (19%)
	Proportion reporting poorest health	0 (0%)	0 (0%)	2 (0%)	1 (0%)	10 (1%)	13 (0%)
EQ-HWB-9	Proportion reporting full health	80 (6%)	45 (4%)	57 (4%)	92 (7%)	49 (4%)	323 (5%)
	Proportion reporting poorest health	0 (0%)	0 (0%)	0 (0%)	3 (0%)	0 (0%)	3 (0%)

* full version of EQ-HWB was administered

Threshold for floor/ceiling effect is 15%

**Table 3:** Convergent validity (total sample)

Value set	EQ-5D-5L							
		Australian value set	Canadian value set	New Zealand value set*	United Kingdom value set*	United States value set	EQ-HWB-9 UK value set	
	n	rho	rho	rho	rho	rho	n	rho
EQ-HWB-9	6448	0.72 (s)	0.73 (s)	0.74 (s)	0.73 (s)	0.73 (s)		
EQ-HWB-25	2594	0.66 (s)	0.68 (s)	0.70 (s)	0.69 (s)	0.68 (s)	2630	0.93 (s)
EQ VAS	6139	0.57 (s)	0.58 (s)	0.59 (s)	0.58 (s)	0.58 (s)	6105	0.54 (s)
WHO-5	3881	0.43 (m)	0.44 (m)	0.47 (m)	0.46 (m)	0.44 (m)	3878	0.54ˆ (s)
PROMIS t-score physical	6566	0.68 (s)	0.69 (s)	0.70 (s)	0.69 (s)	0.70 (s)	6529	0.73 (s)
PROMIS t-score mental	6551	0.54 (s)	0.54 (s)	0.57 (s)	0.57 (s)	0.54 (s)	6513	0.65 (s)
ASCOT SCRQoL	4025	0.54ˆ (s)	0.54ˆ (s)	0.57ˆ (s)	0.56ˆ (s)	0.54ˆ (s)	3991	0.60ˆ (s)
OPQOL-brief	1280	0.51ˆ (s)	0.52ˆ (s)	0.55ˆ (s)	0.55ˆ (s)	0.53ˆ (s)	1272	0.59 (s)
PHQ-2	6599	− 0.49 (m)	− 0.50 (m)	− 0.53ˆ (s)	− 0.53ˆ (s)	− 0.50 (m)	6558	− 0.73ˆ (s)
GAD-2	6585	− 0.50 (m)	− 0.50 (m)	− 0.54ˆ (s)	− 0.53ˆ (s)	− 0.50 (m)	6545	− 0.71ˆ (s)

* full version of EQ-HWB was administered

ˆ - overperformed a priori hypothesis

s, strong, ≥ 0.51; m, moderate, 0.31-0.50; w, weak, 0.11-0.30; and n, none, 0.00-0.10

For the EQ-HWB-9, except for the mobility (36%) domain, at least 50% of respondents reported issues (levels 2–5) with activity (52%), pain (76%), exhaustion (82%), loneliness (62%), cognition (66%), sad/depressed (68%), anxiety (71%) and control (60%) (Fig. 2). At least 5% of respondents reported the worst response for the exhaustion (8%), loneliness (7%), cognition (5%), sad/depressed (7%), anxiety (7%), and control (5%) domains.

Subgroup analyses (Online Appendix 6) show that, compared with 60 years and over, those under 60 years had at least 50% reporting problems on the EQ-5D-5L anxiety/depression dimension, and EQ-HWB-9 activities, loneliness, cognition, and control domains. Across all EQ-5D-5L dimensions, the proportion of respondents reporting problems (levels 2–5) was consistently higher among the financially strained cohort than those classified as financially coping. For the EQ-HWB-9, at least 50% of respondents in both the financially coping and financially strained groups reported having problems (level 2–5) in the pain, exhaustion, loneliness, cognition, sad/depressed, anxious, and control. Less than 50% reported having problems with mobility in either group; however, having problems with activities were reported by a higher proportion of the financially strained group (63%) compared with financially coping (46%). There were no floor effects observed for both the EQ-5D-5L and EQ-HWB-9. For ceiling effects, the EQ-HWB-9 responses indicate no issues (5%), whereas the EQ-5D-5L reached the predefined 15% threshold overall and in four out of the five countries (Table 2). Similarly, the EQ-5D-5L responses reached the 15% threshold in reporting full health subgroups over 60, under 60, and those coping financially (Online Appendix 7).

**Table 4:** Convergent validity by country (using country-specific value sets)

	Australia dataset		Canada dataset		New Zealand* dataset		United Kingdom* dataset		United States dataset	
	n	rho	n	rho	n	rho	n	rho	n	rho
	**EQ-5D-5L country-specific value sets applied to the respective country**									
EQ-HWB-9	1399	0.67 (s)	1198	0.75 (s)	1369	0.72 (s)	1287	0.76 (s)	1195	0.74 (s)
EQ-HWB-25					1334	0.66 (s)	1260	0.72 (s)		
EQ VAS	1346	0.55 (s)	1153	0.61 (s)	1281	0.58 (s)	1211	0.63 (s)	1148	0.55 (s)
WHO-5	1420	0.44 (m)	1237	0.47 (m)					1224	0.41 (m)
PROMIS t-score physical	1414	0.66 (s)	1228	0.72 (s)	1402	0.67 (s)	1309	0.73 (s)	1213	0.69 (s)
PROMIS t-score mental	1412	0.52 (s)	1230	0.55 (s)	1396	0.55 (s)	1299	0.63 (s)	1214	0.53 (s)
ASCOT SCRQoL	1374	0.56ˆ (s)			1369	0.50 (m)	1282	0.60ˆ (s)		
OPQOL-brief	311	0.49ˆ (m)	302	0.59ˆ (s)	257	0.48ˆ (m)	210	0.56ˆ (s)	200	0.58ˆ (s)
PHQ-2	1419	− 0.48 (m)	1231	− 0.50 (m)	1417	− 0.49 (m)	1312	− 0.56ˆ (s)	1220	− 0.53ˆ (s)
GAD-2	1418	− 0.48 (m)	1232	− 0.47 (m)	1411	− 0.51ˆ (s)	1309	− 0.59ˆ (s)	1215	− 0.53ˆ (s)
	**EQ-HWB-9 UK pilot value set applied to the country datasets**									
EQ-5D-5L value set - Australia	1399	0.67 (s)	1198	0.74 (s)	1369	0.69 (s)	1287	0.75 (s)	1195	0.73 (s)
EQ-5D-5L value set - Canada	1399	0.68 (s)	1198	0.75 (s)	1369	0.70 (s)	1287	0.76 (s)	1195	0.73 (s)
EQ-5D-5L value set - New Zealand	1399	0.70 (s)	1198	0.76 (s)	1369	0.72 (s)	1287	0.77 (s)	1195	0.76 (s)
EQ-5D-5L value set - UK	1399	0.69 (s)	1198	0.76 (s)	1369	0.71 (s)	1287	0.76 (s)	1195	0.74 (s)
EQ-5D-5L value set - US	1399	0.68 (s)	1198	0.74 (s)	1369	0.70 (s)	1287	0.75 (s)	1195	0.74 (s)
EQ-HWB-25					1349	0.92 (s)	1281	0.94 (s)		
EQ VAS	1356	0.55 (s)	1144	0.58 (s)	1249	0.49 (m)	1203	0.58 (s)	1153	0.51 (s)
WHO-5	1425	0.52 (s)	1223	0.64 (s)					1230	0.49 (m)
PROMIS t-score physical	1420	0.76 (s)	1216	0.73 (s)	1370	0.71 (s)	1303	0.75 (s)	1220	0.72 (s)
PROMIS t-score mental	1416	0.65 (s)	1218	0.68 (s)	1362	0.63 (s)	1294	0.68 (s)	1223	0.63 (s)
ASCOT SCRQoL	1379	0.58ˆ (s)			1338	0.54ˆ (s)	1274	0.68ˆ (s)		
OPQOL-brief	316	0.56 (s)	299	0.64 (s)	245	0.51 (s)	212	0.62 (s)	200	0.65 (s)
PHQ-2	1423	− 0.75ˆ (s)	1219	− 0.71ˆ (s)	1382	− 0.71ˆ (s)	1306	− 0.73ˆ (s)	1228	− 0.75ˆ (s)
GAD-2	1422	− 0.72ˆ (s)	1221	− 0.67ˆ (s)	1377	− 0.70ˆ (s)	1303	− 0.72ˆ (s)	1222	− 0.74ˆ (s)

* full version of EQ-HWB was administered

ˆ- overperformed a priori hypothesis

s, strong, ≥ 0.51; m, moderate, 0.31–0.50; w, weak, 0.11–0.30; and n, none, 0.00–0.10

### Convergent validity

All a-priori hypotheses were supported, with several correlations exceeding the expected magnitudes (Table 3). Strong correlations were found between the EQ‑5D‑5L value sets and the other measures that represent health constructs (EQ VAS, PROMIS-10). Moderate to strong correlations were observed between the EQ-5D-5L and measures that have overlapping constructs, including wellbeing (WHO-5), health and wellbeing (EQ-HWB-25, EQ-HWB-9), quality of life in the older age (OPQOL-brief), and mental health (PHQ-2, GAD-2) constructs. The correlation with OPQOL-brief was stronger than hypothesized. The New Zealand and UK value sets demonstrated strong correlations with the PHQ‑2 and GAD‑2, while the remaining three country-specific value sets yielded correlations that were under the strong threshold (≥ 0.51). In addition, the EQ-5D-5L displayed a strong correlation with the ASCOT-SCT4, a measure of social care quality of life which was considered conceptually more distant from health or HRQoL.

**Table 5:** Known-groups validity

		EQ-5D-5L					EQ-HWB-9	
	N	Australian value set	Canadian value set	New Zealand value set	United Kingdom value set	United States value set	N	UK pilot value set
*Physical disability*								
No	4064	0.912 (0.13)	0.857 (0.12)	0.859 (0.17)	0.873 (0.14)	0.861 (0.16)	4027	0.789 (0.19)
Yes	2326	0.710 (0.28)	0.665 (0.23)	0.580 (0.32)	0.654 (0.25)	0.573 (0.32)	2318	0.574 (0.28)
		z = 40.7 (39.2, 42.3)	z = 41.4 (40.0, 42.9)	z = 41.0 (39.5, 42.5)	z = 40.7 (39.2, 42.3)	z = 42.5 (41.1, 44.0)		z = 33.2 (31.6, 34.9)
		d = 1.18 (1.12, 1.25)	d = 1.21 (1.15, 1.27)	d = 1.2 (1.14, 1.25)	d = 1.18 (1.12, 1.25)	d = 1.26 (1.20, 1.32)		d = 0.92 (0.86, 0.97)
		ES = L	ES = L	ES = L	ES = L	ES = L		ES = L
*Mental disability*								
No	4208	0.907 (0.14)	0.851 (0.13)	0.850 (0.18)	0.867 (0.15)	0.848 (0.19)	4172	0.807 (0.18)
Yes	2047	0.694 (0.28)	0.653 (0.23)	0.565 (0.32)	0.641 (0.25)	0.565 (0.32)	2039	0.513 (0.27)
		z = 39.3 (37.7, 40.8)	z = 39.8 (38.3, 41.2)	z = 41.1 (39.6, 42.6)	z = 40.5 (39.0, 42.0)	z = 40.0 (38.5, 41.5)		z = 43.0 (41.6, 44.4)
		d = 1.14 (1.08, 1.20)	d = 1.16 (1.11, 1.22)	d = 1.22 (1.16, 1.28)	d = 1.19 (1.13, 1.25)	d = 1.17 (1.11, 1.23)		d = 1.30 (1.24, 1.36)
		ES = L	ES = L	ES = L	ES = L	ES = L		ES = L
*Chronic conditions*								
0 to 1 health condition	2801	0.920 (0.14)	0.865 (0.13)	0.871 (0.19)	0.885 (0.15)	0.871 (0.19)	2777	0.800 (0.20)
2 or more health conditions	3814	0.775 (0.24)	0.726 (0.21)	0.669 (0.29)	0.722 (0.23)	0.666 (0.29)	3793	0.639 (0.27)
		z = 37.4 (35.9, 39.0)	z = 37.6 (36.0, 39.2)	z = 37.6 (36.0, 39.2)	z = 37.3 (35.7, 38.8)	z = 37.7 (36.1, 39.4)		z = 28.4 (26.7, 30.2)
		d = 1.04 (0.98, 1.09)	d = 1.04 (0.99, 1.10)	d = 1.04 (0.99, 1.10)	d = 1.03 (0.98, 1.09)	d = 1.05 (0.99, 1.10)		d = 0.75 (0.70, 0.80)
		ES = L	ES = L	ES = L	ES = L	ES = L		ES = M to L
*Sleep quality*								
Good to very good	2526	0.884 (0.21)	0.832 (0.18)	0.822 (0.27)	0.848 (0.21)	0.822 (0.26)	2510	0.777 (0.25)
Very poor to fair	4076	0.807 (0.22)	0.756 (0.19)	0.713 (0.26)	0.756 (0.21)	0.711 (0.27)	4051	0.664 (0.25)
		z = 23.5 (21.6, 25.4)	z = 23.4 (21.5, 25.2)	z = 24.0 (22.2, 25.9)	z = 23.7 (21.8, 25.7)	z = 22.9 (21.0, 24.7)		z = 23.6 (21.7, 25.6)
		d = 0.56 (0.51, 0.60)	d = 0.55 (0.51, 0.59)	d = 0.57 (0.53, 0.61)	d = 0.56 (0.52, 0.60)	d = 0.54 (0.50, 0.58)		d = 0.56 (0.52, 0.60)
		ES = M	ES = M	ES = M	ES = M	ES = M		ES = M
*Social support*								
Good to excellent	4694	0.861 (0.21)	0.809 (0.18)	0.789 (0.26)	0.819 (0.21)	0.788 (0.26)	4664	0.749 (0.23)
Poor to fair	1865	0.775 (0.24)	0.726 (0.21)	0.672 (0.28)	0.723 (0.22)	0.670 (0.29)	1853	0.605 (0.27)
		z = 19.3 (17.5, 21.2)	z = 19.3 (17.5, 21.0)	z = 20.2 (18.4, 22.0)	z = 20.0 (18.2, 21.8)	z = 19.0 (17.2, 20.9)		z = 22.6 (20.8, 24.4)
		d = 0.46 (0.42, 0.51)	d = 0.46 (0.42, 0.50)	d = 0.48 (0.44, 0.53)	d = 0.48 (0.44, 0.52)	d = 0.46 (0.42, 0.50)		d = 0.54 (0.50, 0.58)
		ES = S to M	ES = S to M	ES = S to M	ES = S to M	ES = S		ES = S to M
*Time for caring*								
Always enough time	3229	0.846 (0.22)	0.797 (0.19)	0.772 (0.28)	0.807 (0.22)	0.771 (0.28)	3221	0.729 (0.26)
Never/sometimes enough time	3355	0.826 (0.21)	0.773 (0.19)	0.738 (0.26)	0.776 (0.21)	0.736 (0.27)	3319	0.685 (0.25)
		z = 7.4 (5.6, 9.3)	z = 7.6 (5.7, 9.5)	z = 8.3 (6.4, 10.1)	z = 8.5 (6.6, 10.5)	z = 7.7 (5.7, 9.6)		z = 10.1 (8.2, 12.0)
		d = 0.18 (0.14, 0.23)	d = 0.19 (0.14, 0.23)	d = 0.20 (0.16, 0.25)	d = 0.21 (0.16, 0.26)	d = 0.19 (0.14, 0.24)		d = 0.25 (0.2, 0.29)
		ES = Nil to S	ES = Nil to S	ES = Nil to S	ES = Nil to S	ES = Nil to S		ES = S
*Impact of caring on own health*								
No impact	1751	0.913 (0.14)	0.859 (0.13)	0.863 (0.18)	0.880 (0.15)	0.861 (0.19)	1767	0.844 (0.18)
Impacts	4864	0.809 (0.24)	0.758 (0.20)	0.715 (0.29)	0.759 (0.23)	0.714 (0.29)	4803	0.657 (0.26)
		z = 21.7 (19.9, 23.6)	z = 22.2 (20.5, 24.0)	z = 23.6 (21.8, 25.4)	z = 23.6 (21.8, 25.4)	z = 22.4 (20.6, 24.2)		z = 31.9 (30.3, 33.6)
		d = 0.56 (0.50, 0.61)	d = 0.57 (0.52, 0.62)	d = 0.61 (0.56, 0.66)	d = 0.61 (0.56, 0.66)	d = 0.57 (0.52, 0.62)		d = 0.86 (0.80, 0.91)
		ES = M	ES = M	ES = M	ES = M	ES = M		ES = L
*Psychological wellbeing*								
Good mental wellbeing	1973	0.882 (0.20)	0.830 (0.17)	0.820 (0.26)	0.845 (0.20)	0.820 (0.25)	1971	0.794 (0.23)
Poor mental wellbeing	4642	0.817 (0.23)	0.766 (0.20)	0.726 (0.27)	0.768 (0.22)	0.725 (0.28)	4599	0.670 (0.25)
		z = 14.9 (13.0, 16.8)	z = 15.4 (13.5, 17.2)	z = 16.8 (15.0, 18.6)	z = 16.5 (14.7, 18.4)	z = 15.6 (13.8, 17.4)		z = 22.5 (20.7, 24.3)
		d = 0.37 (0.32, 0.42)	d = 0.38 (0.34, 0.43.0)	d = 0.42 (0.37, 0.47)	d = 0.42 (0.37, 0.46)	d = 0.39 (0.34, 0.44)		d = 0.58 (0.53, 0.63)
		ES = S	ES = S	ES = S	ES = S	ES = S		ES = M
*Self-reported quality of life*								
Excellent/very good	2935	0.899 (0.19)	0.847 (0.16)	0.844 (0.24)	0.864 (0.19)	0.844 (0.23)	2916	0.798 (0.22)
Good	2204	0.850 (0.17)	0.791 (0.16)	0.759 (0.22)	0.794 (0.17)	0.758 (0.22)	2194	0.720 (0.20)
Poor/Fair	1457	0.690 (0.27)	0.650 (0.23)	0.566 (0.30)	0.640 (0.24)	0.563 (0.31)	1446	0.502 (0.27)
		KW = 1281 (1165, 1397)	KW = 1300 (1182, 1417)	KW = 1400 (1282, 1517)	KW = 1354 (1232, 1475)	KW = 1300 (1180, 1420)		KW = 1520 (1398, 1641)
		d = 0.98 (0.93, 1.04)	d = 0.99 (0.93, 1.05)	d = 1.04 (0.98, 1.09)	d = 1.02 (0.96, 1.07)	d = 0.99 (0.93, 1.05)		d = 1.1 (1.04, 1.16)
		ES = L	ES = L	ES = L	ES = L	ES = L		ES = L

*p*-values: *** < 0.001; ** < 0.01; * < 0.05

Cohen’s d effect sizes: ≥ 0.2 small (S), ≥ 0.5 medium (M) and ≥ 0.8 large (L)

KW, Kruskal-Wallis H-statistic; z,  Mann-Whitney z-score

All correlations examined for the EQ‑HWB‑9 supported the a priori hypotheses. A strong correlation was observed between the EQ-HWB-9 and the full EQ-HWB-25. The EQ-HWB-9 also demonstrated strong correlations with measures assessing overlapping or related constructs, including health (EQ-5D-5L, EQ VAS, and PROMIS-10), wellbeing (WHO-5), quality of life in older age (OPQOL-brief), social care-related quality of life (ASCOT-SCT4), and mental health specific (PHQ-2, GAD2). Notably, the correlations of EQ‑HWB‑9 with the ASCOT-SCT4, PHQ‑2, and GAD‑2 were stronger than anticipated, exceeding the hypothesized moderate correlation strength.

In subgroup analyses (Online Appendix 8), among participants over 60 years, correlations observed supporting the a priori hypotheses. The hypothesized strong correlation between the EQ‑5D‑5L and the PROMIS‑10 mental T‑score was not supported when using the Australian, Canadian, UK, and US value sets within the coping financially subgroup. All other EQ-5D-5L hypothesized relationships were confirmed. For the EQ‑HWB‑9, the correlation strengths observed in the full sample were similarly reflected in the over 60 and financially strained subgroup. However, relationships that were strong in the overall sample, particularly those involving the EQ VAS, WHO‑5 and OPQOL-brief, were reduced to moderate among participants under 60 and those coping financially.

Exploring the performance of EQ-5D-5L by applying country-specific value sets to the respective country-level datasets confirmed main analysis results (Table 4), while using the EQ-HWB-9 pilot value set in country-level datasets also mirrored most main analysis results, with the exception of a smaller magnitude correlation with the EQ VAS in the New Zealand dataset and the WHO-5 in the US dataset. Subgroup analysis mirrored main results (Online Appendix 9).

Of the 24 item-level hypothesized correlations for the EQ-5D-5L, 96% were met (strong correlation, 54%; moderate correlation, 42%) (Online Appendix 10). For the EQ-HWB-9, 37 of 39 (95%) a priori hypotheses were met (strong correlation, 67%; moderate correlation, 28%) (Online Appendix 11).

### Known-group validity

Both EQ-5D-5L and EQ-HWB-9 were able to discriminate between a priori hypothesized groups (*p* < 0.001) (Table 5). For the EQ-5D-5L, large effect sizes were observed in self-reported physical and mental disability, chronic conditions groups, and self-reported QoL; while reduced effect sizes were observed in sleep quality (medium), social support (small to medium), time for caring (nil to small), impact of caring (medium), and psychological wellbeing (small). For the EQ-HWB-9, large effect sizes were observed in physical disability, mental disability, impact of caring groups, and self-reported QoL groups; medium effect sizes in terms of having chronic conditions, sleep quality, social support groups, and psychological wellbeing; and a small effect size for time spent for caring. Country-level analyses for the EQ-5D-5L and EQ-HWB-9 value sets mirrored main known-group validity results (Table 6 and 7). Subgroup analyses support the main findings, where both the EQ-5D-5L and EQ-HWB-9 were able to discriminate between groups(Appendix 12-14)."

### Psychometric performance summary

Results across the difference psychometric criteria are summarized in Table 8. With the exception of ceiling effects in the EQ-5D-5L, both measures showed overall positive psychometric performance across the tested criteria. Results were generally maintained in subgroup analysis (Online Appendix 15).

**Table 6:** Known-groups validity—EQ-5D-5L country-specific value set applied to country-specific dataset

	Australian dataset and value set		Canadian dataset and value set		New Zealand dataset and value set		United Kingdom dataset and value set		United States dataset and value set	
*Physical disability*										
No	861	0.910 (0.13)	730	0.845 (0.12)	890	0.874 (0.15)	871	0.881 (0.14)	712	0.855 (0.17)
Yes	509	0.741 (0.24)	450	0.653 (0.21)	486	0.626 (0.33)	402	0.627 (0.27)	479	0.538 (0.37)
	z = 18.5 (16.9, 20.1)		z = 18.9 (17.5, 20.3)		z = 17.9 (16.3, 19.4)		z = 18.6 (17.0, 20.1)		z = 18.4 (17.0, 19.9)	
	d = 1.15 (1.02, 1.28)		d = 1.32 (1.18, 1.46)		d = 1.10 (0.97, 1.22)		d = 1.22 (1.08, 1.36)		d = 1.26 (1.12, 1.40)	
	ES = L		ES = L		ES = L		ES = L		ES = L	
*Mental disability*										
No	899	0.908 (0.13)	770	0.829 (0.14)	926	0.870 (0.16)	865	0.882 (0.15)	748	0.840 (0.19)
Yes	443	0.714 (0.25)	398	0.656 (0.21)	402	0.606 (0.33)	388	0.619 (0.26)	416	0.526 (0.37)
	z = 18.1 (16.5, 19.6)		z = 15.9 (14.4, 17.4)		z = 18.5 (16.9, 20.1)		z = 19.4 (18.0, 20.8)		z = 17.6 (16.1, 19.1)	
	d = 1.13 (1.01, 1.26)		d = 1.05 (0.92, 1.18)		d = 1.18 (1.04, 1.32)		d = 1.31 (1.18, 1.45)		d = 1.21 (1.07, 1.34)	
	ES = L		ES = L		ES = L		ES = L		ES = L	
*Chronic conditions*										
0 to 1 health condition	574	0.924 (0.12)	475	0.849 (0.13)	652	0.881 (0.19)	640	0.900 (0.14)	460	0.853 (0.24)
2 or more health conditions	846	0.790 (0.22)	762	0.717 (0.19)	767	0.705 (0.28)	675	0.703 (0.25)	764	0.648 (0.32)
	z = 17.1 (15.4, 18.7)		z = 15.4 (13.8, 17.0)		z = 16.6 (15, 18.3)		z = 18.6 (17.1, 20.1)		z = 15.5 (13.9, 17.1)	
	d = 1.02 (0.89, 1.14)		d = 0.97 (0.85, 1.10)		d = 0.98 (0.86, 1.10)		d = 1.20 (1.06, 1.33)		d = 0.99 (0.86, 1.11)	
	ES = L		ES = L		ES = L		ES = L		ES = L	
*Sleep quality*										
Good to very good	556	0.893 (0.17)	436	0.834 (0.14)	548	0.818 (0.30)	475	0.887 (0.17)	511	0.777 (0.34)
Very poor to fair	863	0.813 (0.21)	796	0.734 (0.19)	867	0.766 (0.22)	840	0.749 (0.24)	710	0.688 (0.28)
	z = 10.5 (8.7, 12.3)		z = 10.8 (9.0, 12.6)		z = 8.6 (6.7, 10.5)		z = 13.7 (12.0, 15.4)		z = 9.7 (7.9, 11.5)	
	d = 0.54 (0.45, 0.62)		d = 0.59 (0.50, 0.68)		d = 0.44 (0.35, 0.54)		d = 0.71 (0.63, 0.78)		d = 0.54 (0.44, 0.63)	
	ES = S to M		ES = M		ES = S to M		ES = M		ES = S to M	
*Social support*										
Good to excellent	1,021	0.868 (0.18)	848	0.798 (0.17)	1,010	0.810 (0.26)	890	0.838 (0.19)	925	0.755 (0.30)
Poor to fair	394	0.783 (0.22)	378	0.704 (0.20)	384	0.734 (0.23)	419	0.716 (0.26)	290	0.635 (0.29)
	z = 9.1 (7.3, 10.9)		z = 8.8 (6.9, 10.6)		z = 8.0 (6.1, 9.9)		z = 9.8 (8.0, 11.6)		z = 8.5 (6.8, 10.3)	
	d = 0.47 (0.38, 0.56)		d = 0.49 (0.39, 0.58)		d = 0.42 (0.33, 0.51)		d = 0.52 (0.43, 0.61)		d = 0.47 (0.38, 0.57)	
	ES = S to M		ES = S to M		ES = S to M		ES = S to M		ES = S to M	
*Time for caring*										
Always enough time	752	0.861 (0.19)	573	0.783 (0.18)	660	0.787 (0.29)	627	0.832 (0.20)	617	0.722 (0.34)
Never/sometimes enough time	662	0.826 (0.21)	662	0.754 (0.19)	743	0.787 (0.22)	683	0.768 (0.24)	605	0.728 (0.27)
	z = 4.0 (2.1, 5.9)		z = 3.5 (1.5, 5.5)		z = 2.5 (0.6, 4.4)		z = 5.5 (3.5, 7.4)		z = 1.9 (0.1, 3.9)	
	d = 0.21 (0.11, 0.31)		d = 0.20 (0.09, 0.31)		d = 0.13 (0.03, 0.23)		d = 0.30 (0.19, 0.40)		d = 0.11 (0, 0.22)	
	ES = Nil to S		ES = Nil to S		ES = Nil to S		ES = Nil to S		ES = Nil to S	
*Impact of caring on own health*										
No impact	402	0.917 (0.12)	317	0.840 (0.14)	381	0.873 (0.16)	361	0.894 (0.15)	290	0.848 (0.20)
Impacts	1,018	0.816 (0.21)	920	0.743 (0.19)	1,038	0.754 (0.28)	954	0.763 (0.24)	934	0.687 (0.32)
	z = 11.1 (9.3, 12.9)		z = 9.6 (7.8, 11.4)		z = 8.7 (6.9, 10.5)		z = 11.5 (9.7, 13.2)		z = 9.1 (7.3, 11.0)	
	d = 0.61 (0.50, 0.72)		d = 0.57 (0.45, 0.68)		d = 0.48 (0.37, 0.58)		d = 0.67 (0.55, 0.78)		d = 0.54 (0.42, 0.66)	
	ES = M		ES = S to M		ES = S to M		ES = M		ES = S to M	
*Psychological wellbeing*										
Good mental wellbeing	718	0.899 (0.16)	586	0.838 (0.13)					669	0.789 (0.31)
Poor mental wellbeing	702	0.789 (0.22)	651	0.705 (0.20)					555	0.648 (0.28)
	z = 14.0 (12.2, 15.8)		z = 13.8 (12.1, 15.4)						z = 12.6 (10.8, 14.4)	
	d = 0.80 (0.68, 0.92)		d = 0.85 (0.73, 0.97)						d = 0.77 (0.64, 0.90)	
	ES = M to L		ES = M to L						ES = M to L	
*Self-reported quality of life*										
Excellent/Very good	627	0.903 (0.16)	460	0.846 (0.13)	720	0.851 (0.25)	532	0.899 (0.14)	596	0.802 (0.31)
Good	509	0.853 (0.16)	463	0.785 (0.15)	440	0.772 (0.21)	413	0.821 (0.16)	379	0.716 (0.26)
Poor/Fair	283	0.699 (0.25)	311	0.630 (0.22)	252	0.624 (0.28)	363	0.625 (0.28)	248	0.555 (0.30)
	KW = 265 (212, 317)		KW = 272 (219, 324)		KW = 253 (201, 305)		KW = 354 (298, 410)		KW = 210 (160, 259)	
	d = 0.95 (0.84, 1.07)		d = 1.06 (0.93, 1.19)		d = 0.93 (0.81, 1.05)		d = 1.21 (1.08, 1.35)		d = 0.91 (0.78, 1.04)	
	ES = L		ES = L		ES = L		ES = L		ES = M to L	

*p*-values: *** < 0.001; ** < 0.01; * < 0.05

d: Cohen's d effect sizes: ≥ 0.2 small (S), ≥ 0.5 medium (M) and ≥ 0.8 large (L)

z:  Mann-Whitney z-score; KW:  Kruskal-Wallis H-statistic

## Discussion

This study explored the psychometric performance of the EQ-5D-5L and EQ-HWB-9 in informal carers across multiple countries. Several psychometric properties were explored including distributional (i.e., acceptability, distribution, and floor and ceiling effects) and construct (i.e., convergent, and known-group) validity. Based on a recent systematic review [16], this sample of informal carers is the largest analyzed to date, and this is the first examining Canadian and New Zealand informal carer population, including the use of their country-specific EQ-5D-5L value sets in psychometric validity analysis. In addition, this is the first study that explored the psychometric properties of the EQ-HWB-9 in other English-speaking countries outside of Australia and the US within the informal carer population. Overall, results indicate that both measures have good performance in terms of acceptability and construct validity, particularly in measuring HRQoL (EQ-5D-5L) and health and wellbeing (EQ-HWB-9) outcomes, for use in the informal carer population.

**Table 7:** Known-groups validity—EQ-HWB-9 value set and country-specific dataset

	Australia		Canada		New Zealand		United Kingdom		United States	
*Physical disability*										
No	863	0.774 (0.22)	720	0.775 (0.19)	861	0.808 (0.17)	868	0.802 (0.18)	715	0.782 (0.19)
Yes	514	0.591 (0.27)	446	0.588 (0.26)	479	0.611 (0.26)	398	0.556 (0.30)	481	0.522 (0.30)
	z = 13.9 (12.2, 15.6)		z = 13.4 (11.7, 15.1)		z = 14.9 (13.3, 16.5)		z = 15.5 (13.9, 17.1)		z = 16.2 (14.7, 17.7)	
	d = 0.81 (0.69, 0.92)		d = 0.85 (0.72, 0.98)		d = 0.89 (0.78, 1.01)		d = 0.97 (0.84, 1.09)		d = 1.06 (0.93, 1.19)	
	ES = M to L		ES = M to L		ES = M to L		ES = L		ES = L	
*Mental disability*										
No	902	0.794 (0.20)	759	0.790 (0.18)	898	0.826 (0.16)	860	0.823 (0.17)	753	0.797 (0.18)
Yes	444	0.515 (0.26)	395	0.533 (0.25)	396	0.546 (0.25)	384	0.505 (0.29)	420	0.466 (0.30)
	z = 19.2 (17.8, 20.5)		z = 17.5 (16.0, 19.0)		z = 19.8 (18.5, 21.2)		z = 19.8 (18.4, 21.1)		z = 19.5 (18.2, 20.9)	
	d = 1.23 (1.11, 1.34)		d = 1.2 (1.06, 1.34)		d = 1.32 (1.20, 1.45)		d = 1.36 (1.22, 1.49)		d = 1.39 (1.25, 1.53)	
	ES = L		ES = L		ES = L		ES = L		ES = L	
*Chronic conditions*										
0 to 1 health condition	579	0.787 (0.22)	467	0.789 (0.18)	630	0.811 (0.19)	642	0.820 (0.18)	459	0.787 (0.22)
2 or more health conditions	846	0.643 (0.26)	756	0.642 (0.25)	754	0.673 (0.24)	666	0.626 (0.28)	771	0.607 (0.28)
	z = 11.9 (10.2, 13.7)		z = 10.9 (9.2, 12.7)		z = 12.6 (10.7, 14.4)		z = 14.7 (13.0, 16.4)		z = 12.6 (10.9, 14.3)	
	d = 0.67 (0.56, 0.77)		d = 0.66 (0.54, 0.77)		d = 0.72 (0.60, 0.83)		d = 0.89 (0.76, 1.01)		d = 0.77 (0.65, 0.89)	
	ES = M		ES = M		ES = M to L		ES = M to L		ES = M to L	
*Sleep quality*										
Good to very good	560	0.758 (0.25)	428	0.804 (0.18)	536	0.778 (0.25)	474	0.819 (0.22)	512	0.735 (0.29)
Very poor to fair	864	0.665 (0.25)	791	0.643 (0.24)	846	0.710 (0.21)	834	0.666 (0.26)	716	0.630 (0.25)
	z = 9.1 (7.1, 11.0)		z = 12.5 (10.7, 14.2)		z = 9.5 (7.5, 11.5)		z = 13.1 (11.3, 14.9)		z = 9.7 (7.8, 11.5)	
	d = 0.47 (0.37, 0.56)		d = 0.67 (0.59, 0.76)		d = 0.50 (0.40, 0.59)		d = 0.68 (0.60, 0.76)		d = 0.53 (0.44, 0.63)	
	ES = S to M		ES = M		ES = S to M		ES = M		ES = S to M	
*Social support*										
Good to excellent	1020	0.742 (0.23)	834	0.753 (0.21)	992	0.768 (0.23)	890	0.767 (0.23)	928	0.713 (0.27)
Poor to fair	400	0.595 (0.28)	378	0.584 (0.26)	368	0.658 (0.23)	413	0.623 (0.28)	294	0.552 (0.27)
	z = 10.0 (8.2, 11.8)		z = 11.5 (9.7, 13.3)		z = 10.0 (8.2, 11.7)		z = 10.1 (8.3, 11.8)		z = 9.9 (8.2, 11.6)	
	d = 0.51 (0.43, 0.60)		d = 0.63 (0.54, 0.71)		d = 0.52 (0.44, 0.61)		d = 0.54 (0.45, 0.63)		d = 0.55 (0.46, 0.63)	
	ES = S to M		ES = M		ES = S to M		ES = S to M		ES = S to M	
*Time for caring*										
Always enough time	757	0.729 (0.25)	572	0.730 (0.23)	647	0.742 (0.25)	625	0.766 (0.25)	620	0.679 (0.30)
Never/sometimes enough time	662	0.671 (0.26)	649	0.671 (0.24)	722	0.732 (0.22)	678	0.680 (0.26)	608	0.669 (0.25)
	z = 5.4 (3.5, 7.3)		z = 5.1 (3.1, 7.0)		z = 2.6 (0.6, 4.5)		z = 7.4 (5.5, 9.4)		z = 2.3 (0.4, 4.3)	
	d = 0.28 (0.18, 0.38)		d = 0.29 (0.18, 0.40)		d = 0.14 (0.03, 0.24)		d = 0.40 (0.30, 0.50)		d = 0.13 (0.02, 0.24)	
	ES = Nil to S		ES = Nil to S		ES = Nil to S		ES = S to M		ES = Nil to S	
*Impact of caring on own health*										
No impact	405	0.861 (0.16)	319	0.825 (0.18)	380	0.852 (0.16)	364	0.849 (0.18)	299	0.822 (0.21)
Impacts	1020	0.638 (0.26)	904	0.653 (0.24)	1004	0.692 (0.24)	944	0.673 (0.26)	931	0.627 (0.28)
	z = 17.7 (16.0, 19.3)		z = 12.8 (11.1, 14.5)		z = 13.8 (12.0, 15.5)		z = 13.7 (12, 15.4)		z = 13.0 (11.2, 14.8)	
	d = 1.06 (0.93, 1.18)		d = 0.78 (0.66, 0.91)		d = 0.80 (0.68, 0.92)		d = 0.82 (0.70, 0.94)		d = 0.80 (0.67, 0.93)	
	ES = L		ES = M to L		ES = M to L		ES = M to L		ES = M to L	
*Psychological wellbeing*										
Good mental wellbeing	720	0.800 (0.22)	579	0.825 (0.16)					672	0.760 (0.27)
Poor mental wellbeing	705	0.601 (0.25)	644	0.584 (0.24)					558	0.571 (0.24)
	z = 17.8 (16.1, 19.4)		z = 19.6 (18.2, 21.0)						z = 15.8 (14.0, 17.5)	
	d = 1.07 (0.94, 1.20)		d = 1.35 (1.21, 1.49)						d = 1.01 (0.87, 1.14)	
	ES = L		ES = L						ES = L	
*Self-reported quality of life*										
Excellent/very good	630	0.787 (0.22)	455	0.813 (0.18)	700	0.808 (0.21)	536	0.829 (0.20)	595	0.760 (0.27)
Good	510	0.729 (0.19)	458	0.726 (0.18)	427	0.729 (0.18)	411	0.751 (0.19)	388	0.661 (0.23)
Poor/Fair	284	0.463 (0.27)	308	0.489 (0.25)	251	0.546 (0.24)	356	0.523 (0.29)	247	0.490 (0.26)
	KW = 320 (264, 376)		KW = 351 (297, 405)		KW = 298 (242, 354)		KW = 344 (288, 400)		KW = 242 (192, 292)	
	d = 1.07 (0.95, 1.20)		d = 1.27 (1.13, 1.41)		d = 1.05 (0.92, 1.17)		d = 1.19 (1.06, 1.33)		d = 0.99 (0.86, 1.11)	
	ES = L		ES = L		ES = L		ES = L		ES = L	

All differences between groups were significant (*p *< 0.001)

z,  Mann-Whitney z-score; KW,  Kruskal-Wallis H-statistic; d, Cohen’s d effect size

Cohen's d effect sizes: ≥ 0.2 small (S), ≥ 0.5 medium (M) and ≥ 0.8 large (L)

In summary, the EQ-5D-5L and EQ-HWB-9 performed well across the different psychometric properties explored. There was still a concern around ceiling effect for the EQ-5D-5L, which had mixed evidence in previous studies that focused on informal carers of similar age range (40 years to 52 years). Studies by Sokolova et al. (2024) in dementia informal carers and Kuharic et al. (2024) in general informal carers observed ceiling effect [19, 26], but studies by Faller et al. (2025) in general informal carers and Zhang et al. (2023) in cancer informal carers did not [10, 51]. For the EQ-HWB-9, ceiling effects were not a concern confirming findings from Kuharic et al. (2024) [26]. That absence of ceiling effect in the EQ-HWB-9 could possibly be related to having a broader construct being measured and more items compared with the EQ-5D-5L. Response distribution indicate that the EQ-5D-5L dimensions may not fully reflect impacts of informal care and that informal carers are impacted in other domains, particularly in dimensions around exhaustion, which the EQ-HWB-9 was able to highlight. Both instruments also had at least moderate correlations with the other validated measures that represent similar or overlapping constructs: WHO-5 (wellbeing), PROMIS-10 (health), ASCOT-SCT4 (social care QoL), OPQOL-brief (older person QoL), PHQ-2 (mental health), and GAD-2 (mental health), which are a novel set of observations as the EQ-5D-5L and EQ-HWB-9 have not been compared with these measures previously in this population [16]. Convergence with these other instruments that measure concepts known to be impacted by providing informal care (e.g., emotional, physical health, mental health) support the use the EQ-5D-5L and EQ-HWB-9, as both seem to capture these relevant concepts. Both instruments also performed well in terms of known-group validity and were able to discriminate whether the caring situation affected informal carer health supporting a previously reported observations [26, 52]. The relatively small effect size observed for time for caring groups could be a result of the limitations of the self-reported categorical nature of the item, which may not fully capture the broader impact of caring (e.g., hours spent for caring) as both EQ-5D-5L and EQ-HWB-9 were previously found to being able to discriminate between groups when using hours of care provided per week [26]. Convergent validity and known-group validity analysis findings were consistent among the different EQ-5D-5L country value sets. Findings of the main analysis were also confirmed in subgroup analysis.

**Table 8:** Summary of the psychometric performance of the EQ-5D-5L and EQ-HWB-9

Total sample (*N* = 6,743)						
	EQ-5D-5L Australia value set	EQ-5D-5L Canada value set	EQ-5D-5L New Zealand value set	EQ-5D-5L United Kingdom value set	EQ-5D-5L United States value set	EQ-HWB-9 UK pilot value set
Acceptability (missing)^a^	✔					✔
Floor/ceiling effects^b^	✘					✔
Convergent validity^c^	✔	✔	✔	✔	✔	✔
Known-group validity^d^	✔	✔	✔	✔	✔	✔
	(8/9 groups)	(8/9 groups)	(9/9 groups)	(9/9 groups)	(8/9 groups)	(9/9 groups)
*EQ-5D-5 L country-specific value sets applied to country-specific datasets*						
	Australia dataset and value set	Canada dataset and value set	New Zealand dataset and value set	United Kingdom dataset and value set	United States dataset and value set	
Acceptability (missing)^a^	✔	✔	✔	✔	✔	
Floor/ceiling effects^b^	✘	✔	✘	✘	✘	
Convergent validity^c^	✔	✔	✔	✔	✔	
Known-group validity^d^	✔	✔	✔	✔	✔	
	(9/9 groups)	(8/9 groups)	(7/8 groups)	(8/8 groups)	(8/9 groups)	
*EQ-HWB-9 UK pilot value set applied to country-specific datasets*						
	Australia dataset	Canada dataset	New Zealand dataset	United Kingdom dataset	United States dataset	
Acceptability (missing)^a^	✔	✔	✔	✔	✔	
Floor/ceiling effects^b^	✔	✔	✔	✔	✔	
Convergent validity^c^	✔	✔	✔	✔	✔	
Known-group validity^d^	✔	✔	✔	✔	✔	
	(9/9 groups)	(8/9 groups)	(7/8 groups)	(8/8 groups)	(8/9 groups)	

^a^Acceptability (missing): ✔ below the thresholds of 5% for any item; ✘ above the thresholds of 5% for any item

^b^Floor and ceiling effects: ✔ below the thresholds of 15%; ✘ above the thresholds of 15%

^c^Convergent validity: ✔ strong correlations (≥ 0.60) across majority of correlation coefficients; ± moderate correlations across majority of correlation coefficients (0.40–0.59)

^d^Known-group validity: ✔ at least small effect sizes (≥ 0.20) for at least 75% groups; otherwise ±

The study findings provide valuable evidence around the acceptability of using generic measures in informal carers, thus supporting the use of the EQ-5D-5L and the EQ-HWB-9 to generate QALYs for economic evaluations [10]. Although the study evaluated the EQ-5D-5L and EQ-HWB-9 in the absence of a ‘gold standard’ measure, one does not appear to be superior over the other. A measure with additional dimensions, such as the EQ-HWB-9, certainly provides the ability to capture broader constructs of health and wellbeing. The EQ-HWB-9 includes dimensions such as control, loneliness and exhaustion that extend beyond traditional health status domains. This broader conceptual coverage may make it more sensitive to certain impacts of informal caregiving; however, this reflects measurement of a wider construct of health and wellbeing rather than evidence of superior validity. The EQ-5D-5L and EQ-HWB-9 should also not be considered interchangeable measures of a single construct. Rather, they represent instruments with different conceptual coverage that may capture some overlapping and complementary aspects of informal carers’ outcomes. The selection of which measure to use should still depend on the study purpose. For example, if an economic evaluation is to be submitted to a HTA body, the selection of a measure would be based on agency guidelines. In the UK, the National Institute for Health and Care Excellence (NICE) recommends the EQ-5D-5L for economic evaluations, whereas the Pharmaceutical Benefits Advisory Committee (PBAC) in Australia does not specify a preferred instrument, potentially permitting the use of either the EQ-5D-5L or the EQ-HWB-9 if well justified [53, 54].

The findings remain limited in its capacity to distinguish the extent to which the measures reflect individuals’ overall HRQoL or health and wellbeing, as opposed to effects that can be directly attributed to informal caregiving. Information about the condition of the care recipients was not available, which would have allowed us to conduct further subgroup analysis around the severity of the condition. Further analysis regarding informal caregiver characteristics (e.g., relationship with the care recipient, hours of care provided) were also not possible due to the availability of the data. The study also examined an earlier version of the EQ-HWB, which may potentially differ from the final version. Only the UK pilot EQ-HWB-9 value set was available at the time of the study, which was applied to all countries. This potentially may not reflect preferences of the population in other countries. However, as the primary aim of the study was to explore the psychometric performance rather than compare utility values, we do not believe this substantially weakens the results of the construct validity. Moreover, understanding utility value differences is equally important, and will need to be done when country-specific value sets emerge in the future. Participant recruitment was through an online panel potentially lowering generalizability. Despite the use of quota sampling, there remains a risk of selection bias. Generally, online surveys restrict participation to individuals with access to the internet and who are members of the Dynata online panel. The sample would not be representative of people with lower digital literacy, limited internet access, or possibly lower educational attainment. In addition, the survey sampled the general population while this study only focused on informal carers. Consequently, the analytical sample may not be fully representative of the population of informal carers in each country. The cross-sectional survey also did not allow us to review whether the two instruments can detect changes over time. A longitudinal survey would be ideal if it captures information on both the informal carer and the care recipient, and includes carer-specific measures related to caregiving QoL and burden. This would allow the assessment of the responsiveness of preference-based measures to changes in the care recipient’s health and variations to caregiving burden over time. Similar analyses should still be attempted once country-specific EQ-HWB-9 value sets are available.

## Conclusion

In conclusion, this study provides supporting acceptability and construct validity evidence for both the EQ-5D-5L and EQ-HWB-9 in capturing informal carer HRQoL and health and wellbeing outcomes, respectively, for use in economic evaluations. Responsiveness evidence remains limited but essential in evaluating the broader performance of these measures in this population. Further research is warranted, particularly longitudinal studies that examine the psychometric performance of these measures within the dyadic care recipient and informal carer relationship across the course of the health condition.

## Supplementary Information

Below is the link to the electronic supplementary material.

Supplementary Material 1.

## Data Availability

No datasets were generated or analysed during the current study.
