# Supplementary material for: Are the EQ-5D-5L and EQ-HWB-9 valid instruments for measuring informal carer outcomes for use in economic evaluations?
Source: Qual Life Res. 2026 Oct 15;35(11):307. doi: 10.1007/s11136-026-04410-w (PMC13672468; doi:10.1007/s11136-026-04410-w)
Supplement: Supplementary file 1 [file 11136_2026_4410_MOESM1_ESM.docx]

[**Appendix *1*: A priori hypothesized correlations between the EQ-5D-5L, EQ-HWB-9 and other measures**	1](#_Toc240030139)

[**Appendix *2*: A priori hypotheses to be tested in the known-group validity analysis**	2](#_Toc240030140)

[**Appendix *3*: Demographic characteristics - subgroup analysis**	4](#_Toc240030141)

[**Appendix *4*: Feasibility (missing values)**	5](#_Toc240030142)

[**Appendix *5*: Feasibility (missing values) – subgroup analysis**	6](#_Toc240030143)

[**Appendix *6*: Response distribution – subgroup analysis**	7](#_Toc240030144)

[**Appendix *7*: Ceiling and floor effects – subgroup analysis**	11](#_Toc240030145)

[**Appendix *8*: Convergent validity – subgroup analysis**	12](#_Toc240030146)

[**Appendix *9*: Convergent validity subgroup analysis (EQ-5D-5L country value sets and EQ-HWB-9 UK pilot value set applied to country datasets)**	14](#_Toc240030147)

[**Appendix *10*: Item-level correlation (EQ-5D-5L)**	17](#_Toc240030148)

[**Appendix *11*: Item-level correlation (EQ-HWB-9)**	20](#_Toc240030149)

[**Appendix *12*: Known-groups validity - subgroup analysis (EQ-5D-5L and EQ-HWB-9 value sets in study population)**	23](#_Toc240030150)

[**Appendix *13*: Known-groups validity - subgroup analysis (EQ-5D-5L country value sets in country datasets)**	30](#_Toc240030151)

[**Appendix *14*: Known-groups validity - subgroup analysis (EQ-HWB-9 UK pilot value set in country datasets)**	37](#_Toc240030152)

[**Appendix 15: Summary of psychometric *p*erformance *of the EQ-5D-5L and EQ-HWB-9* - subgroup analysis**	44](#_Toc240030153)

**Appendix *1*: A priori hypothesized correlations between the EQ-5D-5L, EQ-HWB-9 and other measures**

| **Instrument** |  | **EQ-5D-5L** | **EQ-HWB-9** |
| --- | --- | --- | --- |
|  | **Construct** | *Health* | *Health and wellbeing* |
| **EQ-5D-5L** | *Health* | - | Strong correlation |
| **EQ-HWB-9** | *Health and wellbeing* | Strong correlation | - |
| **EQ-HWB-25** | *Health and wellbeing* | Strong correlation | - |
| **EQ-VAS** | *Health* | Strong correlation | Strong correlation |
| **WHO-5** | *Wellbeing* | Moderate correlation | Moderate correlation |
| **PROMIS-10** | *Health* | Strong correlation | Strong correlation |
| **ASCOT** | *Social care quality of life* | Weak correlation | Moderate correlation |
| **OPQOL-brief** | *Quality of life in older age* | Moderate correlation | Strong correlation |
| **PHQ-2** | *Mental health* | Moderate correlation | Moderate correlation |
| **GAD-2** | *Mental health* | Moderate correlation | Moderate correlation |

**Appendix *2*: A priori hypotheses to be tested in the known-group validity analysis**

| **Construct** | **Question in the survey** | **Groups tested** | **Hypothesis** |
| --- | --- | --- | --- |
| Physical disability | *Do you currently experience a physical disability that persisted for 6 months or more?* | Group 1: No  Group 2: Yes | Lower QoL in Group 2 compared with Group 1 |
| Mental disability | *Do you currently experience a mental disability that persisted for 6 months or more?* | Group 1: No  Group 2: Yes | Lower QoL in Group 2 compared with Group 1 |
| Chronic conditions | *Have you ever received a diagnosis for any of the following health conditions from a doctor or other healthcare professional?* (28 conditions presented) | Group 1: 0 to 1 condition  Group 2:  Two or more conditions | Lower QoL in Group 2 compared with Group 1 |
| Sleep quality | *My sleep quality was*: (very poor, poor, fair, good, very good) | Group 1:  good, very good  Group 2:  very poor, poor, fair | Lower QoL in Group 2 compared with Group 1 |
| Social support | *Overall, which of the following best describes your social support network* (Excellent, Very good, Good, Fair, Poor) | Group 1:  Excellent, Very good, Good  Group 2:  Fair, Poor | Lower QoL in Group 2 compared with Group 1 |
| Time for caring | *Thinking about people you have caring responsibilities for, which of the following best describes your current situation?* (1. I always have enough time to care for them; 2. I sometimes have enough time to care for them; 3. I never have enough time to care for them) | Group 1:  Always enough time  Group 2:  Never/Sometimes enough time | Lower QoL in Group 2 compared with Group 1 |
| Impact of caring on own health | *In the last three months, has your own health been affected, in any of the ways listed, by the help or support that you give to the people you care for?* (1. Feeling tired; 2. Feeling depressed; 3. Loss of appetite; 4. Disturbed sleep; 5. General feeling of stress; 6. Physical strain; 7. Short tempered; 8. Developed my own health condition; 9. Made an existing condition worse; 10. No, none of these) | Group 1:  No, none of these  Group 2:  Yes, to any of the 9 areas of health | Lower QoL in Group 2 compared with Group 1 |
| Psychological distress | WHO-5 percentage score: scores greater than or equal to 50 shows good mental wellbeing; scores less than 50 indicates poor mental wellbeing | Group 1:  Good mental wellbeing  Group 2:  Poor mental wellbeing | Lower QoL in Group 2 compared with Group 1 |
| Self-reported quality of life | PROMIS-10 global item on quality-of-life responses: 1 = poor; 2 = fair; 3 = good; 4 = very good; 5 = excellent | Group 1: Poor/Fair quality-of-life  Group 2: Good quality-of-life  Group 3: Excellent/Very good quality-of-life | Gradient increase in QoL from Group1 (lowest QoL) to Group 5 (highest QoL) |

**Appendix *3*: Demographic characteristics - subgroup analysis**

|  | **Over 60** | **Under 60** | **Coping financially** | **Financially strained** |
| --- | --- | --- | --- | --- |
| N (%) | 1,901 (27.8%) | 4,842 (71.8%) | 4,259 (63.4%) | 2,458 (36.6%) |
| Sex at birth – N (%) |  |  |  |  |
| Male | 799 (42.9%) | 2,058 (42.9%) | 2,010 (47.8%) | 831 (34.2%) |
| Female | 1,065 (57.1%) | 2,737 (57.1%) | 2,193 (52.2%) | 1,601 (65.8%) |
| Age – mean (sd) | 69.46 (6.70) | 39.49 (10.82) | 48.30 (17.41) | 46.80 (15.15) |
| Gender – N (%) |  |  |  |  |
| Man | 817 (43.2%) | 2,082 (43.1%) | 2,040 (48.0%) | 841 (34.3%) |
| Woman | 1,074 (56.7%) | 2,689 (55.6%) | 2,175 (51.2%) | 1,580 (64.4%) |
| Non-binary | 1 (0.1%) | 41 (0.8%) | 18 (0.4%) | 24 (1.0%) |
| Transgender Man | 1 (0.1%) | 11 (0.2%) | 8 (0.2%) | 4 (0.2%) |
| Transgender Woman | 0 (0.0%) | 3 (0.1%) | 2 (0.0%) | 1 (0.0%) |
| other | 0 (0.0%) | 9 (0.2%) | 6 (0.1%) | 3 (0.1%) |
| Highest level of education – N (%) |  |  |  |  |
| No formal schooling | 5 (0.3%) | 44 (0.9%) | 26 (0.6%) | 23 (0.9%) |
| Completed primary school (grades 1-6) | 35 (1.9%) | 117 (2.4%) | 76 (1.8%) | 76 (3.1%) |
| Completed secondary school (grades 7-12) | 598 (31.7%) | 1,397 (29.0%) | 1,095 (25.8%) | 895 (36.6%) |
| Completed polytechnic school | 537 (28.4%) | 921 (19.1%) | 812 (19.2%) | 639 (26.1%) |
| Completed Bachelor's degree or equivalent | 486 (25.7%) | 1,602 (33.3%) | 1,461 (34.5%) | 616 (25.2%) |
| Completed Master's degree or equivalent | 177 (9.4%) | 606 (12.6%) | 619 (14.6%) | 163 (6.7%) |
| Completed Doctorate degree or equivalent | 50 (2.6%) | 131 (2.7%) | 147 (3.5%) | 34 (1.4%) |
| Current employment – N (%) |  |  |  |  |
| Stay-at-home parent | 21 (1.1%) | 297 (6.2%) | 163 (3.8%) | 155 (6.3%) |
| Self employed | 120 (6.3%) | 373 (7.7%) | 288 (6.8%) | 205 (8.4%) |
| Employed full-time | 216 (11.4%) | 2,263 (46.9%) | 1,874 (44.1%) | 597 (24.4%) |
| Employed part-time | 193 (10.2%) | 716 (14.8%) | 521 (12.3%) | 386 (15.8%) |
| Unemployed | 56 (3.0%) | 397 (8.2%) | 152 (3.6%) | 299 (12.2%) |
| Retired | 1,169 (61.7%) | 83 (1.7%) | 936 (22.0%) | 309 (12.6%) |
| Student | 4 (0.2%) | 228 (4.7%) | 123 (2.9%) | 109 (4.5%) |
| Unable to work due to disability or illness | 83 (4.4%) | 363 (7.5%) | 124 (2.9%) | 320 (13.1%) |
| On maternity / paternity leave | 1 (0.1%) | 23 (0.5%) | 13 (0.3%) | 11 (0.4%) |
| other | 31 (1.6%) | 81 (1.7%) | 55 (1.3%) | 57 (2.3%) |
| Health and wellbeing measures – mean (sd) |  |  |  |  |
| EQ-5D-5L index AU, country specific | 0.860 (0.177) | 0.827 (0.234) | 0.871 (0.196) | 0.776 (0.243) |
| EQ-5D-5L index CA, country specific | 0.803 (0.162) | 0.778 (0.203) | 0.818 (0.173) | 0.728 (0.211) |
| EQ-5D-5L index NZ, country specific | 0.781 (0.226) | 0.744 (0.287) | 0.800 (0.251) | 0.674 (0.286) |
| EQ-5D-5L index UK, country specific | 0.811 (0.183) | 0.783 (0.226) | 0.828 (0.198) | 0.727 (0.229) |
| EQ-5D-5L index US, country specific | 0.773 (0.233) | 0.745 (0.285) | 0.799 (0.249) | 0.674 (0.292) |
| EQ-VAS | 72.62 (19.33) | 69.74 (21.89) | 75.12 (19.07) | 62.82 (22.35) |
| EQ-HWB score | 104.21 (15.06) | 93.50 (18.34) | 99.66 (17.20) | 90.00 (18.33) |
| EQ-HWB-9 index | 0.787 (0.205) | 0.675 (0.262) | 0.755 (0.231) | 0.625 (0.267) |
| WHO-5 global score | 9.137 (8.483) | 6.774 (7.520) | 8.248 (8.407) | 6.061 (6.625) |
| Promis-10 t-score physical | 46.58 (8.79) | 45.53 (8.84) | 47.58 (8.44) | 42.809 (8.70) |
| Promis-10 t-score mental | 48.26 (9.48) | 44.82 (10.08) | 48.81 (9.02) | 40.58 (9.58) |
| ASCOT social care-related quality of life | 0.834 (0.163) | 0.754 (0.221) | 0.835 (0.165) | 0.670 (0.238) |
| OPQOL score | 54.77 (7.48) | na | 56.40 (6.45) | 50.22 (8.22) |
| PHQ-2 score | 1.017 (1.429) | 2.120 (1.747) | 1.491 (1.612) | 2.353 (1.805) |
| GAD-2 score | 1.110 (1.469) | 2.268 (1.828) | 1.572 (1.647) | 2.570 (1.904) |

**Appendix *4*: Feasibility (missing values)**

|  | **Australia** | **Canada** | **New Zealand*** | **United Kingdom*** | **United States** | **Total** |
| --- | --- | --- | --- | --- | --- | --- |
| N | 1,447 | 1,265 | 1,435 | 1,337 | 1,259 | 6,743 |
| **EQ-5D-5L** |  |  |  |  |  |  |
| Mobility | 6 (0%) | 2 (0%) | 5 (0%) | 3 (0%) | 5 (0%) | 21 (0%) |
| Self-care | 5 (0%) | 8 (1%) | 7 (0%) | 7 (1%) | 3 (0%) | 30 (0%) |
| Usual activities | 6 (0%) | 13 (1%) | 4 (0%) | 7 (1%) | 8 (1%) | 38 (1%) |
| Pain/  discomfort | 7 (0%) | 7 (1%) | 1 (0%) | 4 (0%) | 12 (1%) | 31 (0%) |
| Anxiety/  depression | 5 (0%) | 3 (0%) | 4 (0%) | 4 (0%) | 8 (1%) | 24 (0%) |
| **EQ-HWB-9** |  |  |  |  |  |  |
| Activities | 0 (0%) | 13 (1%) | 5 (0%) | 1 (0%) | 0 (0%) | 19 (0%) |
| Mobility | 0 (0%) | 16 (1%) | 4 (0%) | 2 (0%) | 3 (0%) | 25 (0%) |
| Pain | 3 (0%) | 21 (2%) | 4 (0%) | 2 (0%) | 5 (0%) | 35 (1%) |
| Exhaustion | 5 (0%) | 17 (1%) | 1 (0%) | 4 (0%) | 3 (0%) | 30 (0%) |
| Loneliness | 3 (0%) | 19 (2%) | 6 (0%) | 5 (0%) | 5 (0%) | 38 (1%) |
| Cognition | 1 (0%) | 18 (1%) | 11 (1%) | 4 (0%) | 5 (0%) | 39 (1%) |
| Sad/  depressed | 5 (0%) | 18 (1%) | 5 (0%) | 2 (0%) | 7 (1%) | 37 (1%) |
| Anxiety | 8 (1%) | 21 (2%) | 11 (1%) | 6 (0%) | 7 (1%) | 53 (1%) |
| Control | 2 (0%) | 17 (1%) | 15 (1%) | 13 (1%) | 7 (1%) | 54 (1%) |

* full version of EQ-HWB was administered

**Appendix *5*: Feasibility (missing values) – subgroup analysis**

|  | **Over 60** | **Under 60** |  | **Coping financially** | **Financially strained** |
| --- | --- | --- | --- | --- | --- |
| N | 1,901 | 4,842 |  | 4,259 | 2,458 |
| **EQ-5D-5L** |  |  |  |  |  |
| Mobility | 7 (0%) | 14 (0%) |  | 11 (0%) | 10 (0%) |
| Self-care | 14 (1%) | 16 (0%) |  | 21 (0%) | 9 (0%) |
| Usual activities | 5 (0%) | 33 (1%) |  | 26 (1%) | 12 (0%) |
| Pain/  discomfort | 6 (0%) | 25 (1%) |  | 21 (0%) | 10 (0%) |
| Anxiety/  depression | 4 (0%) | 20 (0%) |  | 15 (0%) | 9 (0%) |
| **EQ-HWB-9** |  |  |  |  |  |
| Activities | 4 (0%) | 15 (0%) |  | 10 (0%) | 9 (0%) |
| Mobility | 5 (0%) | 20 (0%) |  | 12 (0%) | 13 (1%) |
| Pain | 5 (0%) | 30 (1%) |  | 13 (0%) | 22 (1%) |
| Exhaustion | 5 (0%) | 25 (1%) |  | 17 (0%) | 13 (1%) |
| Loneliness | 11 (1%) | 27 (1%) |  | 19 (0%) | 19 (1%) |
| Cognition | 5 (0%) | 34 (1%) |  | 22 (1%) | 17 (1%) |
| Sad/  depressed | 10 (1%) | 27 (1%) |  | 22 (1%) | 15 (1%) |
| Anxiety | 12 (1%) | 41 (1%) |  | 37 (1%) | 16 (1%) |
| Control | 18 (1%) | 36 (1%) |  | 35 (1%) | 19 (1%) |

**Appendix *6*: Response distribution – subgroup analysis**

EQ-5D-5L over 60

|  | Level 1 | Level 2 | Level 3 | Level 4 | Level 5 | Missing |
| --- | --- | --- | --- | --- | --- | --- |
| Anxiety and Depression | 1040 | 532 | 239 | 70 | 16 | 4 |
| Pain and Discomfort | 471 | 830 | 439 | 139 | 16 | 6 |
| Usual activities | 1107 | 512 | 218 | 53 | 6 | 5 |
| Self care | 1614 | 205 | 57 | 6 | 5 | 14 |
| Mobility | 1049 | 500 | 257 | 81 | 7 | 7 |

|  | L1 | L2 | L3 | L4 | L5 | miss |
| --- | --- | --- | --- | --- | --- | --- |
| MO | 1049 (55%) | 500 (26%) | 257 (14%) | 81 (4%) | 7 (0%) | 7 (0%) |
| SC | 1614 (85%) | 205 (11%) | 57 (3%) | 6 (0%) | 5 (0%) | 14 (1%) |
| UA | 1107 (58%) | 512 (27%) | 218 (11%) | 53 (3%) | 6 (0%) | 5 (0%) |
| PD | 471 (25%) | 830 (44%) | 439 (23%) | 139 (7%) | 16 (1%) | 6 (0%) |
| AD | 1040 (55%) | 532 (28%) | 239 (13%) | 70 (4%) | 16 (1%) | 4 (0%) |

EQ-5D-5L under 60

|  | Level 1 | Level 2 | Level 3 | Level 4 | Level 5 | Missing |
| --- | --- | --- | --- | --- | --- | --- |
| Anxiety and Depression | 1637 | 1505 | 954 | 431 | 295 | 20 |
| Pain and Discomfort | 1760 | 1836 | 801 | 304 | 116 | 25 |
| Usual activities | 3057 | 1024 | 497 | 183 | 48 | 33 |
| Self care | 3848 | 599 | 254 | 82 | 43 | 16 |
| Mobility | 3360 | 890 | 399 | 132 | 47 | 14 |

|  | L1 | L2 | L3 | L4 | L5 | miss |
| --- | --- | --- | --- | --- | --- | --- |
| MO | 3360 (69%) | 890 (18%) | 399 (8%) | 132 (3%) | 47 (1%) | 14 (0%) |
| SC | 3848 (79%) | 599 (12%) | 254 (5%) | 82 (2%) | 43 (1%) | 16 (0%) |
| UA | 3057 (63%) | 1024 (21%) | 497 (10%) | 183 (4%) | 48 (1%) | 33 (1%) |
| PD | 1760 (36%) | 1836 (38%) | 801 (17%) | 304 (6%) | 116 (2%) | 25 (1%) |
| AD | 1637 (34%) | 1505 (31%) | 954 (20%) | 431 (9%) | 295 (6%) | 20 (0%) |

EQ-HWB-9 over 60

|  | Level 1 | Level 2 | Level 3 | Level 4 | Level 5 | Missing |
| --- | --- | --- | --- | --- | --- | --- |
| Control | 1062 | 426 | 228 | 105 | 62 | 18 |
| Anxious | 887 | 571 | 260 | 124 | 47 | 12 |
| Sad/depressed | 944 | 528 | 263 | 102 | 54 | 10 |
| Cognition | 1025 | 525 | 237 | 83 | 26 | 5 |
| Loneliness | 1094 | 408 | 230 | 108 | 50 | 11 |
| Exhaustion | 491 | 741 | 411 | 178 | 75 | 5 |
| Pain | 356 | 894 | 493 | 140 | 13 | 5 |
| Mobility | 1283 | 375 | 178 | 57 | 3 | 5 |
| Activities | 1026 | 520 | 235 | 105 | 11 | 4 |

|  | L1 | L2 | L3 | L4 | L5 | miss |
| --- | --- | --- | --- | --- | --- | --- |
| Activities | 1026 (54%) | 520 (27%) | 235 (12%) | 105 (6%) | 11 (1%) | 4 (0%) |
| Mobility | 1283 (67%) | 375 (20%) | 178 (9%) | 57 (3%) | 3 (0%) | 5 (0%) |
| Pain | 356 (19%) | 894 (47%) | 493 (26%) | 140 (7%) | 13 (1%) | 5 (0%) |
| Exhaustion | 491 (26%) | 741 (39%) | 411 (22%) | 178 (9%) | 75 (4%) | 5 (0%) |
| Loneliness | 1094 (58%) | 408 (21%) | 230 (12%) | 108 (6%) | 50 (3%) | 11 (1%) |
| Cognition | 1025 (54%) | 525 (28%) | 237 (12%) | 83 (4%) | 26 (1%) | 5 (0%) |
| Sad/depressed | 944 (50%) | 528 (28%) | 263 (14%) | 102 (5%) | 54 (3%) | 10 (1%) |
| Anxious | 887 (47%) | 571 (30%) | 260 (14%) | 124 (7%) | 47 (2%) | 12 (1%) |
| Control | 1062 (56%) | 426 (22%) | 228 (12%) | 105 (6%) | 62 (3%) | 18 (1%) |

EQ-HWB-9 under 60

|  | Level 1 | Level 2 | Level 3 | Level 4 | Level 5 | Missing |
| --- | --- | --- | --- | --- | --- | --- |
| Control | 1621 | 1254 | 1032 | 598 | 301 | 36 |
| Anxious | 1059 | 1233 | 1245 | 813 | 451 | 41 |
| Sad/depressed | 1179 | 1350 | 1183 | 682 | 421 | 27 |
| Cognition | 1217 | 1311 | 1215 | 741 | 324 | 34 |
| Loneliness | 1479 | 1171 | 1034 | 739 | 392 | 27 |
| Exhaustion | 719 | 1399 | 1270 | 934 | 495 | 25 |
| Pain | 1270 | 2069 | 1007 | 348 | 118 | 30 |
| Mobility | 3002 | 857 | 615 | 303 | 45 | 20 |
| Activities | 2201 | 1280 | 828 | 457 | 61 | 15 |

|  | L1 | L2 | L3 | L4 | L5 | miss |
| --- | --- | --- | --- | --- | --- | --- |
| Activities | 2201 (45%) | 1280 (26%) | 828 (17%) | 457 (9%) | 61 (1%) | 15 (0%) |
| Mobility | 3002 (62%) | 857 (18%) | 615 (13%) | 303 (6%) | 45 (1%) | 20 (0%) |
| Pain | 1270 (26%) | 2069 (43%) | 1007 (21%) | 348 (7%) | 118 (2%) | 30 (1%) |
| Exhaustion | 719 (15%) | 1399 (29%) | 1270 (26%) | 934 (19%) | 495 (10%) | 25 (1%) |
| Loneliness | 1479 (31%) | 1171 (24%) | 1034 (21%) | 739 (15%) | 392 (8%) | 27 (1%) |
| Cognition | 1217 (25%) | 1311 (27%) | 1215 (25%) | 741 (15%) | 324 (7%) | 34 (1%) |
| Sad/depressed | 1179 (24%) | 1350 (28%) | 1183 (24%) | 682 (14%) | 421 (9%) | 27 (1%) |
| Anxious | 1059 (22%) | 1233 (25%) | 1245 (26%) | 813 (17%) | 451 (9%) | 41 (1%) |
| Control | 1621 (33%) | 1254 (26%) | 1032 (21%) | 598 (12%) | 301 (6%) | 36 (1%) |

EQ-5D-5L coping financially

|  | Level 1 | Level 2 | Level 3 | Level 4 | Level 5 | Missing |
| --- | --- | --- | --- | --- | --- | --- |
| Anxiety and Depression | 2084 | 1290 | 555 | 202 | 113 | 15 |
| Pain and Discomfort | 1647 | 1713 | 627 | 188 | 63 | 21 |
| Usual activities | 2883 | 891 | 334 | 94 | 31 | 26 |
| Self care | 3526 | 450 | 172 | 56 | 34 | 21 |
| Mobility | 2944 | 814 | 343 | 111 | 36 | 11 |

|  | L1 | L2 | L3 | L4 | L5 | miss |
| --- | --- | --- | --- | --- | --- | --- |
| Mobility | 2944 (69%) | 814 (19%) | 343 (8%) | 111 (3%) | 36 (1%) | 11 (0%) |
| Self care | 3526 (83%) | 450 (11%) | 172 (4%) | 56 (1%) | 34 (1%) | 21 (0%) |
| Usual activities | 2883 (68%) | 891 (21%) | 334 (8%) | 94 (2%) | 31 (1%) | 26 (1%) |
| Pain/ discomfort | 1647 (39%) | 1713 (40%) | 627 (15%) | 188 (4%) | 63 (1%) | 21 (0%) |
| Anxiety/ depression | 2084 (49%) | 1290 (30%) | 555 (13%) | 202 (5%) | 113 (3%) | 15 (0%) |

EQ-5D-5L financially strained

|  | Level 1 | Level 2 | Level 3 | Level 4 | Level 5 | Missing |
| --- | --- | --- | --- | --- | --- | --- |
| Anxiety and Depression | 588 | 729 | 635 | 299 | 198 | 9 |
| Pain and Discomfort | 578 | 936 | 610 | 255 | 69 | 10 |
| Usual activities | 1270 | 634 | 378 | 142 | 22 | 12 |
| Self care | 1919 | 348 | 137 | 32 | 13 | 9 |
| Mobility | 1454 | 564 | 311 | 102 | 17 | 10 |

|  | L1 | L2 | L3 | L4 | L5 | miss |
| --- | --- | --- | --- | --- | --- | --- |
| Mobility | 1454 (59%) | 564 (23%) | 311 (13%) | 102 (4%) | 17 (1%) | 10 (0%) |
| Self care | 1919 (78%) | 348 (14%) | 137 (6%) | 32 (1%) | 13 (1%) | 9 (0%) |
| Usual activities | 1270 (52%) | 634 (26%) | 378 (15%) | 142 (6%) | 22 (1%) | 12 (0%) |
| Pain/ discomfort | 578 (24%) | 936 (38%) | 610 (25%) | 255 (10%) | 69 (3%) | 10 (0%) |
| Anxiety/ depression | 588 (24%) | 729 (30%) | 635 (26%) | 299 (12%) | 198 (8%) | 9 (0%) |

EQ-HWB-9 coping financially

|  | Level 1 | Level 2 | Level 3 | Level 4 | Level 5 | Missing |
| --- | --- | --- | --- | --- | --- | --- |
| Control | 2005 | 1036 | 696 | 334 | 153 | 35 |
| Anxious | 1510 | 1198 | 883 | 444 | 187 | 37 |
| Sad/depressed | 1645 | 1246 | 803 | 363 | 180 | 22 |
| Cognition | 1704 | 1162 | 820 | 412 | 139 | 22 |
| Loneliness | 1897 | 1021 | 727 | 436 | 159 | 19 |
| Exhaustion | 973 | 1465 | 1046 | 524 | 234 | 17 |
| Pain | 1219 | 1920 | 810 | 221 | 76 | 13 |
| Mobility | 2818 | 733 | 442 | 222 | 32 | 12 |
| Activities | 2308 | 1061 | 556 | 279 | 45 | 10 |

|  | L1 | L2 | L3 | L4 | L5 | miss |
| --- | --- | --- | --- | --- | --- | --- |
| Activities | 2308 (54%) | 1061 (25%) | 556 (13%) | 279 (7%) | 45 (1%) | 10 (0%) |
| Mobility | 2818 (66%) | 733 (17%) | 442 (10%) | 222 (5%) | 32 (1%) | 12 (0%) |
| Pain | 1219 (29%) | 1920 (45%) | 810 (19%) | 221 (5%) | 76 (2%) | 13 (0%) |
| Exhaustion | 973 (23%) | 1465 (34%) | 1046 (25%) | 524 (12%) | 234 (5%) | 17 (0%) |
| Loneliness | 1897 (45%) | 1021 (24%) | 727 (17%) | 436 (10%) | 159 (4%) | 19 (0%) |
| Cognition | 1704 (40%) | 1162 (27%) | 820 (19%) | 412 (10%) | 139 (3%) | 22 (1%) |
| Sad/depressed | 1645 (39%) | 1246 (29%) | 803 (19%) | 363 (9%) | 180 (4%) | 22 (1%) |
| Anxious | 1510 (35%) | 1198 (28%) | 883 (21%) | 444 (10%) | 187 (4%) | 37 (1%) |
| Control | 2005 (47%) | 1036 (24%) | 696 (16%) | 334 (8%) | 153 (4%) | 35 (1%) |

EQ-HWB-9 financially strained

|  | Level 1 | Level 2 | Level 3 | Level 4 | Level 5 | Missing |
| --- | --- | --- | --- | --- | --- | --- |
| Control | 672 | 636 | 556 | 367 | 208 | 19 |
| Anxious | 433 | 599 | 612 | 488 | 310 | 16 |
| Sad/depressed | 474 | 622 | 634 | 419 | 294 | 15 |
| Cognition | 532 | 665 | 625 | 408 | 211 | 17 |
| Loneliness | 672 | 551 | 527 | 406 | 283 | 19 |
| Exhaustion | 235 | 664 | 629 | 585 | 332 | 13 |
| Pain | 402 | 1029 | 684 | 266 | 55 | 22 |
| Mobility | 1457 | 491 | 346 | 135 | 16 | 13 |
| Activities | 908 | 733 | 500 | 282 | 26 | 9 |

|  | L1 | L2 | L3 | L4 | L5 | miss |
| --- | --- | --- | --- | --- | --- | --- |
| Activities | 908 (37%) | 733 (30%) | 500 (20%) | 282 (11%) | 26 (1%) | 9 (0%) |
| Mobility | 1457 (59%) | 491 (20%) | 346 (14%) | 135 (5%) | 16 (1%) | 13 (1%) |
| Pain | 402 (16%) | 1029 (42%) | 684 (28%) | 266 (11%) | 55 (2%) | 22 (1%) |
| Exhaustion | 235 (10%) | 664 (27%) | 629 (26%) | 585 (24%) | 332 (14%) | 13 (1%) |
| Loneliness | 672 (27%) | 551 (22%) | 527 (21%) | 406 (17%) | 283 (12%) | 19 (1%) |
| Cognition | 532 (22%) | 665 (27%) | 625 (25%) | 408 (17%) | 211 (9%) | 17 (1%) |
| Sad/depressed | 474 (19%) | 622 (25%) | 634 (26%) | 419 (17%) | 294 (12%) | 15 (1%) |
| Anxious | 433 (18%) | 599 (24%) | 612 (25%) | 488 (20%) | 310 (13%) | 16 (1%) |
| Control | 672 (27%) | 636 (26%) | 556 (23%) | 367 (15%) | 208 (8%) | 19 (1%) |

**Appendix *7*: Ceiling and floor effects – subgroup analysis**

|  |  | **Over 60** | **Under 60** |  | **Coping financially** | **Financially strained** |
| --- | --- | --- | --- | --- | --- | --- |
|  |  |  |  |  |  |  |
| **EQ-5D-5L** | Proportion reporting full health | 308 (16%) | 959 (20%) |  | 1039 (24%) | 224 (9%) |
|  | Proportion reporting poorest health | 0 (0%) | 13 (0%) |  | 12 (0%) | 1 (0%) |
| **EQ-HWB-9** | Proportion reporting full health | 95 (5%) | 228 (5%) |  | 289 (7%) | 32 (1%) |
|  | Proportion reporting poorest health | 0 (0%) | 3 (0%) |  | 3 (0%) | 0 (0%) |

**Appendix *8*: Convergent validity – subgroup analysis**

|  | **Over 60** | | | | | | | |
| --- | --- | --- | --- | --- | --- | --- | --- | --- |
|  | **EQ-5D-5L** | | | | | | **EQ-HWB-9** | |
|  |  | **Australian value set** | **Canadian value set** | **New Zealand value set** | **United Kingdom value set** | **United States value set** | **UK pilot value set** | |
|  | n | rho | rho | rho | rho | rho | n | rho |
| EQ-HWB-9 | 1,831 | 0.82 (s) | 0.83 (s) | 0.85 (s) | 0.84 (s) | 0.82 (s) |  |  |
| EQ-HWB-25 | 648 | 0.73 (s) | 0.74 (s) | 0.76 (s) | 0.76 (s) | 0.73 (s) | 655 | 0.89 (s) |
| EQ-VAS | 1,826 | 0.65 (s) | 0.65 (s) | 0.66 (s) | 0.65 (s) | 0.65 (s) | 1,817 | 0.63 (s) |
| WHO-5 global | 1,186 | **0.58 (s)** | **0.59 (s)** | **0.62 (s)** | **0.61 (s)** | **0.59 (s)** | 1,189 | **0.71 (s)** |
| PROMIS t-score physical | 1,857 | 0.79 (s) | 0.80 (s) | 0.80 (s) | 0.79 (s) | 0.81 (s) | 1,850 | 0.80 (s) |
| PROMIS t-score mental | 1,852 | 0.56 (s) | 0.57 (s) | 0.61 (s) | 0.61 (s) | 0.56 (s) | 1,847 | 0.69 (s) |
| ASCOT SCRQoL | 1,083 | **0.50 (m)** | **0.51 (s)** | **0.54 (s)** | **0.54 (s)** | **0.51 (s)** | 1,075 | **0.59 (s)** |
| OPQOL-brief | 1,280 | **0.51 (s)** | **0.52 (s)** | **0.55 (s)** | **0.55 (s)** | **0.53 (s)** | 1,272 | 0.59 (s) |
| PHQ-2 | 1,865 | -0.49 (m) | -0.50 (m) | **-0.53 (s)** | **-0.54 (s)** | -0.49 (m) | 1,859 | **-0.65 (s)** |
| GAD-2 | 1,866 | -0.46 (m) | -0.46 (m) | **-0.51 (s)** | **-0.52 (s)** | -0.46 (m) | 1,860 | **-0.62 (s)** |
|  |  |  |  |  |  |  |  |  |
|  | **Under 60** | | | | | | | |
|  | **EQ-5D-5L** | | | | | | **EQ-HWB-9** | |
|  |  | **Australian value set** | **Canadian value set** | **New Zealand value set** | **United Kingdom value set** | **United States value set** | **UK pilot value set** | |
|  | n | rho | rho | rho | rho | rho | n | rho |
| EQ-HWB-9 | 4,617 | 0.70 (s) | 0.71 (s) | 0.72 (s) | 0.71 (s) | 0.71 (s) |  |  |
| EQ-HWB-25 | 1,946 | 0.68 (s) | 0.69 (s) | 0.71 (s) | 0.70 (s) | 0.70 (s) | 1,975 | 0.94 (s) |
| EQ-VAS | 4,313 | 0.55 (s) | 0.55 (s) | 0.56 (s) | 0.55 (s) | 0.55 (s) | 4,288 | **0.50 (m)** |
| WHO-5 global | 2,695 | 0.38 (m) | 0.39 (m) | 0.40 (m) | 0.40 (m) | 0.39 (m) | 2,689 | 0.44 (m) |
| PROMIS t-score physical | 4,709 | 0.65 (s) | 0.65 (s) | 0.66 (s) | 0.65 (s) | 0.66 (s) | 4,679 | 0.71 (s) |
| PROMIS t-score mental | 4,699 | 0.54 (s) | 0.54 (s) | 0.56 (s) | 0.55 (s) | 0.53 (s) | 4,666 | 0.61 (s) |
| ASCOT SCRQoL | 2,942 | **0.55 (s)** | **0.55 (s)** | **0.57 (s)** | **0.56 (s)** | **0.56 (s)** | 2,916 | **0.57 (s)** |
| OPQOL-brief | |  |  |  |  |  |  |  |
| PHQ-2 | 4,734 | **-0.53 (s)** | **-0.53 (s)** | **-0.55 (s)** | **-0.55 (s)** | **-0.54 (s)** | 4,699 | **-0.73 (s)** |
| GAD-2 | 4,719 | **-0.54 (s)** | **-0.54 (s)** | **-0.56 (s)** | **-0.56 (s)** | **-0.55 (s)** | 4,685 | **-0.71 (s)** |
|  |  |  |  |  |  |  |  |  |
|  | **Coping financially** | | | | | | | |
|  | **EQ-5D-5L** | | | | | | **EQ-HWB-9** | |
|  |  | **Australian value set** | **Canadian value set** | **New Zealand value set** | **United Kingdom value set** | **United States value set** | **UK pilot value set** | |
|  | n |  |  |  |  |  | n | rho |
| EQ-HWB-9 | 4,072 | 0.66 (s) | 0.67 (s) | 0.69 (s) | 0.68 (s) | 0.67 (s) |  |  |
| EQ-HWB-25 | 1,652 | 0.60 (s) | 0.62 (s) | 0.64 (s) | 0.63 (s) | 0.62 (s) | 1,679 | 0.92 (s) |
| EQ-VAS | 3,872 | 0.51 (s) | 0.52 (s) | 0.53 (s) | 0.52 (s) | 0.52 (s) | 3,853 | **0.48 (m)** |
| WHO-5 global | 2,425 | 0.36 (m) | 0.37 (m) | 0.40 (m) | 0.40 (m) | 0.38 (m) | 2,429 | 0.47 (m) |
| PROMIS t-score physical | 4,148 | 0.63 (s) | 0.64 (s) | 0.65 (s) | 0.64 (s) | 0.65 (s) | 4,128 | 0.72 (s) |
| PROMIS t-score mental | 4,134 | **0.47 (m)** | **0.47 (m)** | 0.51 (s) | **0.50 (m)** | **0.47 (m)** | 4,114 | 0.59 (s) |
| ASCOT SCRQoL | 2,551 | 0.48 (m) | 0.49 (m) | 0.51 (s) | 0.50 (m) | 0.48 (m) | 2,529 | 0.51 (s) |
| OPQOL-brief | 933 | 0.44 (m) | 0.45 (m) | 0.48 (m) | 0.48 (m) | 0.45 (m) | 928 | **0.50 (m)** |
| PHQ-2 | 4,165 | -0.43 (m) | -0.44 (m) | -0.47 (m) | -0.47 (m) | -0.44 (m) | 4,143 | **-0.71 (s)** |
| GAD-2 | 4,153 | -0.45 (m) | -0.45 (m) | -0.49 (m) | -0.49 (m) | -0.45 (m) | 4,131 | **-0.70 (s)** |
|  |  |  |  |  |  |  |  |  |
|  | **Financially strained** | | | | | | | |
|  | **EQ-5D-5L** | | | | | | **EQ-HWB-9** | |
|  |  | **Australian value set** | **Canadian value set** | **New Zealand value set** | **United Kingdom value set** | **United States value set** | **UK pilot value set** | |
|  | n | rho | rho | rho | rho | rho | n | rho |
| EQ-HWB-9 | 2,350 | 0.76 (s) | 0.77 (s) | 0.78 (s) | 0.77 (s) | 0.77 (s) |  |  |
| EQ-HWB-25 | 927 | 0.70 (s) | 0.71 (s) | 0.73 (s) | 0.72 (s) | 0.71 (s) | 936 | 0.94 (s) |
| EQ-VAS | 2,248 | 0.60 (s) | 0.60 (s) | 0.61 (s) | 0.59 (s) | 0.60 (s) | 2,233 | 0.56 (s) |
| WHO-5 global | 1,447 | 0.44 (m) | 0.44 (m) | 0.46 (m) | 0.45 (m) | 0.44 (m) | 1,440 | 0.60 (s) |
| PROMIS t-score physical | 2,393 | 0.71 (s) | 0.72 (s) | 0.72 (s) | 0.71 (s) | 0.72 (s) | 2,376 | 0.71 (s) |
| PROMIS t-score mental | 2,391 | 0.55 (s) | 0.55 (s) | 0.57 (s) | 0.56 (s) | 0.54 (s) | 2,373 | 0.70 (s) |
| ASCOT SCRQoL | 1,452 | **0.51 (s)** | **0.52 (s)** | **0.55 (s)** | **0.54 (s)** | **0.53 (s)** | 1,440 | **0.66 (s)** |
| OPQOL-brief | 341 | 0.48 (m) | **0.51 (s)** | **0.52 (s)** | **0.53 (s)** | **0.51 (s)** | 338 | 0.60 (s) |
| PHQ-2 | 2,409 | **-0.51 (s)** | **-0.52 (s)** | **-0.54 (s)** | **-0.53 (s)** | **-0.51 (s)** | 2,390 | **-0.71 (s)** |
| GAD-2 | 2,407 | -0.49 (m) | -0.49 (m) | **-0.51 (s)** | **-0.51 (s)** | -0.48 (m) | 2,389 | **-0.67 (s)** |
| * full version of EQ-HWB was administered green highlight - overperformed a priori hypothesis amber highlight - hypothesised correlation not met s – strong, ≥ 0.51; m - moderate, 0.31-0.50; w – weak, 0.11-0.30; and n – none, 0.00-0.10 | | | | | | | | |

**Appendix *9*: Convergent validity subgroup analysis (EQ-5D-5L country value sets and EQ-HWB-9 UK pilot value set applied to country datasets)**

|  | **Over 60** | | | | | | | | | |
| --- | --- | --- | --- | --- | --- | --- | --- | --- | --- | --- |
|  | **Australia dataset** | | **Canada dataset** | | **New Zealand* dataset** | | **United Kingdom* dataset** | | **United States dataset** | |
|  | **EQ-5D-5L country-specific value sets applied to the respective country** | | | | | | | | | |
|  | n | rho | n | rho | n | rho | n | rho | n | rho |
| EQ-HWB-9 | 426 | 0.79 (s) | 432 | 0.83 (s) | 336 | 0.83 (s) | 329 | 0.86 (s) | 308 | 0.87 (s) |
| EQ-HWB-25 |  |  |  |  | 328 | 0.73 (s) | 320 | 0.79 (s) |  |  |
| EQ-VAS | 420 | 0.63 (s) | 432 | 0.67 (s) | 340 | 0.70 (s) | 328 | 0.62 (s) | 306 | 0.68 (s) |
| WHO-5 | 430 | **0.55 (s)** | 443 | **0.61 (s)** |  |  |  |  | 313 | **0.60 (s)** |
| PROMIS t-score physical | 428 | 0.77 (s) | 441 | 0.83 (s) | 347 | 0.79 (s) | 331 | 0.78 (s) | 310 | 0.82 (s) |
| PROMIS t-score mental | 426 | 0.53 (s) | 438 | 0.63 (s) | 344 | 0.56 (s) | 333 | 0.62 (s) | 311 | 0.61 (s) |
| ASCOT SCRQoL | 418 | **0.53 (s)** |  |  | 337 | **0.44 (m)** | 328 | **0.61 (s)** |  |  |
| OPQOL-brief | 311 | 0.49 (m) | 302 | **0.59 (s)** | 257 | 0.48 (m) | 210 | **0.56 (s)** | 200 | **0.58 (s)** |
| PHQ-2 | 430 | -0.48 (m) | 439 | -0.55 (s) | 349 | -0.40 (m) | 334 | **-0.58 (s)** | 313 | **-0.53 (s)** |
| GAD-2 | 430 | -0.42 (m) | 440 | -0.48 (m) | 349 | -0.39 (m) | 334 | **-0.59 (s)** | 313 | **-0.54 (s)** |
|  | **EQ-HWB-9 value set applied to the country datasets** | | | | | | | | | |
|  | n | rho | n | rho | n | rho | n | rho | n | rho |
| EQ-5D-5L value set - Australia | 426 | 0.79 (s) | 432 | 0.82 (s) | 336 | 0.81 (s) | 329 | 0.84 (s) | 308 | 0.85 (s) |
| EQ-5D-5L value set - Canada | 426 | 0.80 (s) | 432 | 0.83 (s) | 336 | 0.82 (s) | 329 | 0.85 (s) | 308 | 0.86 (s) |
| EQ-5D-5L value set - New Zealand | 426 | 0.81 (s) | 432 | 0.85 (s) | 336 | 0.83 (s) | 329 | 0.87 (s) | 308 | 0.89 (s) |
| EQ-5D-5L value set - UK | 426 | 0.81 (s) | 432 | 0.84 (s) | 336 | 0.82 (s) | 329 | 0.86 (s) | 308 | 0.88 (s) |
| EQ-5D-5L value set - US | 426 | 0.79 (s) | 432 | 0.82 (s) | 336 | 0.81 (s) | 329 | 0.84 (s) | 308 | 0.87 (s) |
| EQ-HWB-25 |  |  |  |  | 329 | 0.85 (s) | 326 | 0.91 (s) |  |  |
| EQ-VAS | 424 | 0.56 (s) | 427 | 0.64 (s) | 328 | 0.66 (s) | 329 | 0.64 (s) | 309 | 0.69 (s) |
| WHO-5 | 434 | 0.66 (s) | 438 | 0.73 (s) |  |  |  |  | 317 | 0.74 (s) |
| PROMIS t-score physical | 432 | 0.77 (s) | 437 | 0.81 (s) | 335 | 0.78 (s) | 332 | 0.80 (s) | 314 | 0.84 (s) |
| PROMIS t-score mental | 430 | 0.67 (s) | 435 | 0.73 (s) | 332 | 0.62 (s) | 334 | 0.71 (s) | 316 | 0.74 (s) |
| ASCOT SCRQoL | 422 | **0.57 (s)** |  |  | 325 | **0.51 (s)** | 328 | **0.69 (s)** |  |  |
| OPQOL-brief | 316 | 0.56 (s) | 299 | 0.64 (s) | 245 | 0.51 (s) | 212 | 0.62 (s) | 200 | 0.65 (s) |
| PHQ-2 | 434 | **-0.67 (s)** | 436 | **-0.69 (s)** | 337 | **-0.49 (m)** | 335 | **-0.68 (s)** | 317 | **-0.69 (s)** |
| GAD-2 | 434 | **-0.60 (s)** | 437 | **-0.63 (s)** | 337 | **-0.50 (m)** | 335 | **-0.68 (s)** | 317 | **-0.68 (s)** |
|  |  |  |  |  |  |  |  |  |  |  |
|  | **Under 60** | | | | | | | | | |
|  | **Australia dataset** | | **Canada dataset** | | **New Zealand* dataset** | | **United Kingdom* dataset** | | **United States dataset** | |
|  | **EQ-5D-5L country-specific value sets applied to the respective country** | | | | | | | | | |
|  | n | rho | n | rho | n | rho | n | rho | n | rho |
| EQ-HWB-9 | 973 | 0.65 (s) | 766 | 0.74 (s) | 1,033 | 0.71 (s) | 958 | 0.75 (s) | 887 | 0.72 (s) |
| EQ-HWB-25 |  |  |  |  | 1,006 | 0.68 (s) | 940 | 0.72 (s) |  |  |
| EQ-VAS | 926 | 0.52 (s) | 721 | 0.58 (s) | 941 | 0.54 (s) | 883 | 0.63 (s) | 842 | 0.51 (s) |
| WHO-5 | 990 | 0.40 (m) | 794 | 0.41 (m) |  |  |  |  | 911 | 0.35 (m) |
| PROMIS t-score physical | 986 | 0.62 (s) | 787 | 0.67 (s) | 1,055 | 0.63 (s) | 978 | 0.71 (s) | 903 | 0.64 (s) |
| PROMIS t-score mental | 986 | 0.51 (s) | 792 | 0.53 (s) | 1,052 | 0.55 (s) | 966 | 0.64 (s) | 903 | 0.51 (s) |
| ASCOT SCRQoL | 956 | **0.57 (s)** |  |  | 1,032 | **0.52 (s)** | 954 | **0.60 (s)** |  |  |
| OPQOL-brief |  |  |  |  |  |  |  |  |  |  |
| PHQ-2 | 989 | -0.49 (m) | 792 | **-0.51 (s)** | 1,068 | **-0.57 (s)** | 978 | **-0.58 (s)** | 907 | **-0.55 (s)** |
| GAD-2 | 988 | **-0.52 (s)** | 792 | -0.49 (m) | 1,062 | **-0.59 (s)** | 975 | **-0.60 (s)** | 902 | **-0.55 (s)** |
|  | **EQ-HWB-9 value set applied to the country datasets** | | | | | | | | | |
|  | n | rho | n | rho | n | rho | n | rho | n | rho |
| EQ-5D-5L value set - Australia | 973 | 0.65 (s) | 766 | 0.73 (s) | 1,033 | 0.68 (s) | 958 | 0.74 (s) | 887 | 0.70 (s) |
| EQ-5D-5L value set - Canada | 973 | 0.65 (s) | 766 | 0.74 (s) | 1,033 | 0.69 (s) | 958 | 0.75 (s) | 887 | 0.71 (s) |
| EQ-5D-5L value set - New Zealand | 973 | 0.66 (s) | 766 | 0.74 (s) | 1,033 | 0.71 (s) | 958 | 0.75 (s) | 887 | 0.72 (s) |
| EQ-5D-5L value set - UK | 973 | 0.66 (s) | 766 | 0.74 (s) | 1,033 | 0.70 (s) | 958 | 0.75 (s) | 887 | 0.71 (s) |
| EQ-5D-5L value set - US | 973 | 0.66 (s) | 766 | 0.74 (s) | 1,033 | 0.70 (s) | 958 | 0.75 (s) | 887 | 0.72 (s) |
| EQ-HWB-25 |  |  |  |  | 1,020 | 0.94 (s) | 955 | 0.94 (s) |  |  |
| EQ-VAS | 932 | 0.53 (s) | 717 | 0.53 (s) | 921 | **0.44 (m)** | 874 | 0.56 (s) | 844 | **0.45 (m)** |
| WHO-5 | 991 | 0.42 (m) | 785 | **0.53 (s)** |  |  |  |  | 913 | 0.39 (m) |
| PROMIS t-score physical | 988 | 0.76 (s) | 779 | 0.69 (s) | 1,035 | 0.69 (s) | 971 | 0.74 (s) | 906 | 0.68 (s) |
| PROMIS t-score mental | 986 | 0.59 (s) | 783 | 0.62 (s) | 1,030 | 0.59 (s) | 960 | 0.66 (s) | 907 | 0.57 (s) |
| ASCOT SCRQoL | 957 | **0.54 (s)** |  |  | 1,013 | **0.52 (s)** | 946 | **0.65 (s)** |  |  |
| OPQOL-brief |  |  |  |  |  |  |  |  |  |  |
| PHQ-2 | 989 | **-0.75 (s)** | 783 | **-0.66 (s)** | 1,045 | **-0.75 (s)** | 971 | **-0.71 (s)** | 911 | **-0.75 (s)** |
| GAD-2 | 988 | **-0.71 (s)** | 784 | **-0.63 (s)** | 1,040 | **-0.73 (s)** | 968 | **-0.70 (s)** | 905 | **-0.74 (s)** |
|  |  |  |  |  |  |  |  |  |  |  |
|  | **Coping financially** | | | | | | | | | |
|  | **Australia dataset** | | **Canada dataset** | | **New Zealand* dataset** | | **United Kingdom* dataset** | | **United States dataset** | |
|  | **EQ-5D-5L country-specific value sets applied to the respective country** | | | | | | | | | |
|  | n | rho | n | rho | n | rho | n | rho | n | rho |
| EQ-HWB-9 | 869 | 0.61 (s) | 694 | 0.70 (s) | 892 | 0.67 (s) | 804 | 0.71 (s) | 813 | 0.68 (s) |
| EQ-HWB-25 |  |  |  |  | 867 | 0.60 (s) | 785 | 0.67 (s) |  |  |
| EQ-VAS | 841 | **0.46 (m)** | 668 | 0.56 (s) | 833 | 0.51 (s) | 752 | 0.59 (s) | 778 | **0.49 (m)** |
| WHO-5 | 880 | 0.36 (m) | 716 | 0.43 (m) |  |  |  |  | 829 | 0.34 (m) |
| PROMIS t-score physical | 876 | 0.61 (s) | 711 | 0.69 (s) | 920 | 0.63 (s) | 817 | 0.68 (s) | 824 | 0.63 (s) |
| PROMIS t-score mental | 876 | **0.43 (m)** | 710 | **0.49 (m)** | 916 | 0.48 (m) | 812 | 0.58 (s) | 820 | 0.47 (m) |
| ASCOT SCRQoL | 856 | **0.54 (s)** |  |  | 894 | **0.43 (m)** | 801 | **0.53 (s)** |  |  |
| OPQOL-brief | 208 | 0.39 (m) | 222 | **0.57 (s)** | 178 | 0.42 (m) | 176 | 0.48 (m) | 149 | 0.49 (m) |
| PHQ-2 | 879 | -0.41 (m) | 710 | -0.43 (m) | 931 | -0.45 (m) | 819 | -0.50 (m) | 826 | -0.47 (m) |
| GAD-2 | 878 | -0.42 (m) | 712 | -0.40 (m) | 926 | -0.50 (m) | 815 | **-0.54 (s)** | 822 | -0.48 (m) |
|  | **EQ-HWB-9 value set applied to the country datasets** | | | | | | | | | |
|  | n | rho | n | rho | n | rho | n | rho | n | rho |
| EQ-5D-5L value set - Australia | 869 | 0.61 (s) | 694 | 0.69 (s) | 892 | 0.65 (s) | 804 | 0.69 (s) | 813 | 0.66 (s) |
| EQ-5D-5L value set - Canada | 869 | 0.62 (s) | 694 | 0.70 (s) | 892 | 0.66 (s) | 804 | 0.70 (s) | 813 | 0.67 (s) |
| EQ-5D-5L value set - New Zealand | 869 | 0.63 (s) | 694 | 0.72 (s) | 892 | 0.67 (s) | 804 | 0.72 (s) | 813 | 0.70 (s) |
| EQ-5D-5L value set - UK | 869 | 0.63 (s) | 694 | 0.72 (s) | 892 | 0.67 (s) | 804 | 0.71 (s) | 813 | 0.69 (s) |
| EQ-5D-5L value set - US | 869 | 0.62 (s) | 694 | 0.70 (s) | 892 | 0.66 (s) | 804 | 0.70 (s) | 813 | 0.68 (s) |
| EQ-HWB-25 |  |  |  |  | 877 | 0.91 (s) | 802 | 0.92 (s) |  |  |
| EQ-VAS | 853 | 0.48 (m) | 662 | 0.55 (s) | 804 | 0.40 (m) | 751 | 0.53 (s) | 783 | 0.47 (m) |
| WHO-5 | 888 | 0.43 (m) | 706 | 0.62 (s) |  |  |  |  | 835 | 0.39 (m) |
| PROMIS t-score physical | 884 | 0.75 (s) | 702 | 0.71 (s) | 893 | 0.70 (s) | 818 | 0.73 (s) | 831 | 0.71 (s) |
| PROMIS t-score mental | 883 | 0.58 (s) | 702 | 0.62 (s) | 887 | 0.56 (s) | 814 | 0.62 (s) | 828 | 0.58 (s) |
| ASCOT SCRQoL | 863 | **0.48 (m)** |  |  | 866 | **0.44 (m)** | 800 | **0.62 (s)** |  |  |
| OPQOL-brief | 212 | 0.46 (m) | 220 | 0.59 (s) | 169 | 0.41 (m) | 177 | 0.54 (s) | 150 | 0.54 (s) |
| PHQ-2 | 886 | **-0.73 (s)** | 702 | **-0.69 (s)** | 901 | **-0.70 (s)** | 820 | **-0.69 (s)** | 834 | **-0.73 (s)** |
| GAD-2 | 885 | **-0.71 (s)** | 704 | **-0.66 (s)** | 897 | **-0.70 (s)** | 816 | **-0.67 (s)** | 829 | **-0.74 (s)** |
|  |  |  |  |  |  |  |  |  |  |  |
|  | **Financially strained** | | | | | | | | | |
|  | **Australia dataset** | | **Canada dataset** | | **New Zealand* dataset** | | **United Kingdom* dataset** | | **United States dataset** | |
|  | **EQ-5D-5L country-specific value sets applied to the respective country** | | | | | | | | | |
|  | n | rho | n | rho | n | rho | n | rho | n | rho |
| EQ-HWB-9 | 524 | 0.71 (s) | 502 | 0.76 (s) | 467 | 0.77 (s) | 476 | 0.79 (s) | 381 | 0.79 (s) |
| EQ-HWB-25 |  |  |  |  | 459 | 0.72 (s) | 468 | 0.73 (s) |  |  |
| EQ-VAS | 500 | 0.59 (s) | 483 | 0.62 (s) | 444 | 0.62 (s) | 452 | 0.63 (s) | 369 | 0.59 (s) |
| WHO-5 | 534 | 0.44 (m) | 519 | 0.45 (m) |  |  |  |  | 394 | 0.43 (m) |
| PROMIS t-score physical | 532 | 0.69 (s) | 515 | 0.73 (s) | 473 | 0.71 (s) | 485 | 0.74 (s) | 388 | 0.73 (s) |
| PROMIS t-score mental | 530 | **0.50 (m)** | 518 | 0.56 (s) | 470 | 0.60 (s) | 480 | 0.62 (s) | 393 | 0.52 (s) |
| ASCOT SCRQoL | 512 | **0.45 (m)** |  |  | 465 | **0.56 (s)** | 475 | **0.59 (s)** |  |  |
| OPQOL-brief | 100 | **0.53 (s)** | 80 | **0.53 (s)** | 78 | **0.53 (s)** | 32 | **0.54 (s)** | 51 | 0.40 (m) |
| PHQ-2 | 534 | -0.48 (m) | 519 | **-0.53 (s)** | 477 | -0.48 (m) | 486 | **-0.58 (s)** | 393 | **-0.54 (s)** |
| GAD-2 | 534 | -0.49 (m) | 518 | -0.46 (m) | 476 | -0.46 (m) | 487 | **-0.56 (s)** | 392 | -0.49 (m) |
|  | **EQ-HWB-9 value set applied to the country datasets** | | | | | | | | | |
|  | n | rho | n | rho | n | rho | n | rho | n | rho |
| EQ-5D-5L value set - Australia | 524 | 0.71 (s) | 502 | 0.76 (s) | 467 | 0.74 (s) | 476 | 0.78 (s) | 381 | 0.78 (s) |
| EQ-5D-5L value set - Canada | 524 | 0.72 (s) | 502 | 0.76 (s) | 467 | 0.75 (s) | 476 | 0.79 (s) | 381 | 0.79 (s) |
| EQ-5D-5L value set - New Zealand | 524 | 0.73 (s) | 502 | 0.77 (s) | 467 | 0.77 (s) | 476 | 0.80 (s) | 381 | 0.80 (s) |
| EQ-5D-5L value set - UK | 524 | 0.72 (s) | 502 | 0.76 (s) | 467 | 0.76 (s) | 476 | 0.79 (s) | 381 | 0.79 (s) |
| EQ-5D-5L value set - US | 524 | 0.72 (s) | 502 | 0.76 (s) | 467 | 0.75 (s) | 476 | 0.79 (s) | 381 | 0.79 (s) |
| EQ-HWB-25 |  |  |  |  | 464 | 0.94 (s) | 472 | 0.94 (s) |  |  |
| EQ-VAS | 498 | 0.59 (s) | 480 | 0.55 (s) | 441 | 0.59 (s) | 445 | 0.59 (s) | 369 | 0.50 (s) |
| WHO-5 | 531 | **0.61 (s)** | 515 | **0.60 (s)** |  |  |  |  | 394 | **0.60 (s)** |
| PROMIS t-score physical | 530 | 0.75 (s) | 512 | 0.71 (s) | 468 | 0.70 (s) | 478 | 0.72 (s) | 388 | 0.69 (s) |
| PROMIS t-score mental | 527 | 0.72 (s) | 514 | 0.70 (s) | 465 | 0.74 (s) | 473 | 0.70 (s) | 394 | 0.66 (s) |
| ASCOT SCRQoL | 510 | **0.63 (s)** |  |  | 462 | **0.67 (s)** | 468 | **0.67 (s)** |  |  |
| OPQOL-brief | 101 | 0.59 (s) | 79 | 0.68 (s) | 75 | 0.58 (s) | 33 | 0.54 (s) | 50 | 0.51 (s) |
| PHQ-2 | 531 | **-0.74 (s)** | 515 | **-0.67 (s)** | 472 | **-0.69 (s)** | 479 | **-0.72 (s)** | 393 | **-0.73 (s)** |
| GAD-2 | 531 | **-0.67 (s)** | 515 | **-0.61 (s)** | 471 | **-0.65 (s)** | 480 | **-0.73 (s)** | 392 | **-0.67 (s)** |
| * full version of EQ-HWB was administered green highlight - overperformed a priori hypothesis amber highlight - hypothesised correlation not met s – strong, ≥ 0.51; m - moderate, 0.31-0.50; w – weak, 0.11-0.30; and n – none, 0.00-0.10 | | | | | | | | | | |

**Appendix *10*: Item-level correlation (EQ-5D-5L)**

| **Measure** | **Dimension / question** | **Mobility** | **Self-care** | **Usual activities** | **Pain / discomfort** | **Anxiety / depression** |
| --- | --- | --- | --- | --- | --- | --- |
| EQ-HWB-9 | Activities | 0.54 | 0.48 | 0.63 | 0.49 | 0.40 |
| EQ-HWB-9 | Mobility | 0.58 | 0.51 | 0.53 | 0.40 | 0.25 |
| EQ-HWB-9 | Pain | 0.51 | 0.37 | 0.52 | 0.73 | 0.36 |
| EQ-HWB-9 | Exhaustion | 0.28 | 0.27 | 0.39 | 0.38 | 0.49 |
| EQ-HWB-9 | Loneliness | 0.20 | 0.27 | 0.27 | 0.23 | 0.53 |
| EQ-HWB-9 | Cognition | 0.23 | 0.29 | 0.35 | 0.28 | 0.55 |
| EQ-HWB-9 | Sad/ depressed | 0.20 | 0.27 | 0.30 | 0.26 | 0.67 |
| EQ-HWB-9 | Anxious | 0.18 | 0.26 | 0.29 | 0.25 | 0.69 |
| EQ-HWB-9 | Control | 0.25 | 0.32 | 0.35 | 0.28 | 0.48 |
| WHO-5 | I have felt cheerful and in good spirits | -0.14 | -0.16 | -0.24 | -0.20 | -0.45 |
| WHO-5 | I have felt calm and relaxed | -0.14 | -0.18 | -0.24 | -0.21 | -0.50 |
| WHO-5 | I have felt active and vigorous | -0.25 | -0.18 | -0.33 | -0.31 | -0.40 |
| WHO-5 | I woke up feeling fresh and rested | -0.20 | -0.13 | -0.30 | -0.30 | -0.43 |
| WHO-5 | My daily life has been filled with things that interest me | -0.15 | -0.14 | -0.24 | -0.22 | -0.43 |
| PROMIS-10 | In general, would you say your health is: | -0.35 | -0.20 | -0.41 | -0.46 | -0.38 |
| PROMIS-10 | In general, would you say your quality of life is: | -0.26 | -0.18 | -0.34 | -0.35 | -0.43 |
| PROMIS-10 | In general, how would rate your physical health? | -0.37 | -0.21 | -0.42 | -0.47 | -0.35 |
| PROMIS-10 | In general, how would rate your mental health, including you mood and ability to think? | -0.15 | -0.14 | -0.26 | -0.27 | -0.62 |
| PROMIS-10 | In general, how would rate your satisfaction with your social activities and relationships? | -0.19 | -0.15 | -0.28 | -0.29 | -0.46 |
| PROMIS-10 | To what extent are you able to carry out your everyday physical activities such as walking, climbing stairs, carrying groceries, or moving a chair? | -0.47 | -0.38 | -0.46 | -0.56 | -0.30 |
| PROMIS-10 | How would you rate your pain on average? | -0.19 | -0.21 | -0.29 | -0.28 | -0.68 |
| PROMIS-10 | How would you rate your fatigue on average? | -0.28 | -0.23 | -0.38 | -0.35 | -0.44 |
| PROMIS-10 | In general, please rate how well you carry out your usual social activities and roles. (This includes activities at home, at work and in your community, and responsibilities as a parent, child, spouse, employee, friend, etc.) | -0.51 | -0.39 | -0.52 | -0.43 | -0.27 |
| PROMIS-10 | How often have you been bothered by emotional problems such as feeling anxious, depressed or irritable? | -0.34 | -0.33 | -0.42 | -0.38 | -0.47 |
| ASCOT | Which of the following statements best describes how much control you have over your daily life? | 0.24 | 0.24 | 0.32 | 0.31 | 0.41 |
| ASCOT | Thinking about your personal care, by which we mean being clean and presentable in appearance, which of the following statements best describes your situation? | 0.29 | 0.36 | 0.36 | 0.25 | 0.41 |
| ASCOT | Thinking about the food and drink you get, which of the following statements best describes your situation? | 0.19 | 0.28 | 0.27 | 0.21 | 0.37 |
| ASCOT | Which of the following statements best describes how safe you feel? | 0.23 | 0.28 | 0.30 | 0.26 | 0.38 |
| ASCOT | Thinking about how much contact you’ve had with people you like, which of the following statements best describes your social situation? | 0.20 | 0.21 | 0.29 | 0.26 | 0.41 |
| ASCOT | Which of the following statements best describes how you spend your time? | 0.18 | 0.15 | 0.29 | 0.31 | 0.42 |
| ASCOT | Which of the following statements best describes how clean and comfortable your home is? | 0.23 | 0.24 | 0.30 | 0.24 | 0.38 |
| ASCOT | Which of these statements best describes how having help to do things makes you think and feel about yourself? | 0.17 | 0.13 | 0.19 | 0.25 | 0.24 |
| ASCOT | Thinking about the way you are helped and treated, and how that makes you think and feel about yourself, which of these statements best describes your situation? | 0.20 | 0.16 | 0.23 | 0.24 | 0.26 |
| OPQOL | Thinking about both the good and bad things that make up your quality of life, how would you rate the quality of your life as a whole? | -0.26 | -0.27 | -0.33 | -0.30 | -0.46 |
| OPQOL | I enjoy my life overall | -0.26 | -0.27 | -0.33 | -0.30 | -0.46 |
| OPQOL | I look forward to things | -0.25 | -0.27 | -0.30 | -0.27 | -0.41 |
| OPQOL | I am healthy enough to get out and about | -0.53 | -0.41 | -0.54 | -0.44 | -0.34 |
| OPQOL | My family, friends or neighbours would help me if needed | -0.14 | -0.17 | -0.15 | -0.16 | -0.22 |
| OPQOL | I have social or leisure activities/hobbies that I enjoy doing | -0.28 | -0.29 | -0.31 | -0.27 | -0.32 |
| OPQOL | I try to stay involved with things | -0.26 | -0.24 | -0.31 | -0.24 | -0.31 |
| OPQOL | I am healthy enough to have my independence | -0.48 | -0.43 | -0.52 | -0.39 | -0.29 |
| OPQOL | I can please myself what I do | -0.30 | -0.30 | -0.34 | -0.28 | -0.32 |
| OPQOL | I feel safe where I live | -0.22 | -0.22 | -0.25 | -0.22 | -0.27 |
| OPQOL | I get pleasure from my home | -0.25 | -0.22 | -0.25 | -0.22 | -0.32 |
| OPQOL | I take life as it comes and make the best of things | -0.15 | -0.17 | -0.20 | -0.18 | -0.41 |
| OPQOL | I feel lucky compared to most people | -0.19 | -0.24 | -0.27 | -0.26 | -0.33 |
| OPQOL | I have enough money to pay for household bills | -0.25 | -0.20 | -0.30 | -0.27 | -0.32 |
| PHQ-2 | Little interest or pleasure in doing things | 0.23 | 0.29 | 0.33 | 0.26 | 0.52 |
| PHQ-2 | Feeling down, depressed, or hopeless | 0.22 | 0.30 | 0.31 | 0.24 | 0.63 |
| GAD-2 | Feeling nervous, anxious, or on edge | 0.19 | 0.26 | 0.29 | 0.25 | 0.66 |
| GAD-2 | Not being able to stop or control worrying | 0.20 | 0.26 | 0.29 | 0.24 | 0.60 |
| s – strong correlation, ≥ 0.51; m – moderate correlation, 0.31-0.50; w – weak correlation, 0.11-0.30; and n – none, 0.00-0.10 | | | | | | |

**Appendix *11*: Item-level correlation (EQ-HWB-9)**

| **Measure** | **Dimension / question** | **Activities** | **Mobility** | **Pain** | **Exhaustion** | **Loneliness** | **Cognition** | **Sad/ depressed** | **Anxious** | **Control** |
| --- | --- | --- | --- | --- | --- | --- | --- | --- | --- | --- |
| EQ-5D-5L | Mobility | 0.54 | 0.58 | 0.51 | 0.28 | 0.20 | 0.23 | 0.20 | 0.18 | 0.25 |
| EQ-5D-5L | Self- care | 0.48 | 0.51 | 0.37 | 0.27 | 0.27 | 0.29 | 0.27 | 0.26 | 0.32 |
| EQ-5D-5L | Usual activities | 0.63 | 0.53 | 0.52 | 0.39 | 0.27 | 0.35 | 0.30 | 0.29 | 0.35 |
| EQ-5D-5L | Pain discomfort | 0.49 | 0.40 | 0.73 | 0.38 | 0.23 | 0.28 | 0.26 | 0.25 | 0.28 |
| EQ-5D-5L | Anxiety depression | 0.40 | 0.25 | 0.36 | 0.49 | 0.53 | 0.55 | 0.67 | 0.69 | 0.48 |
| WHO-5 | I have felt cheerful and in good spirits | -0.32 | -0.22 | -0.22 | -0.34 | -0.42 | -0.41 | -0.51 | -0.46 | -0.42 |
| WHO-5 | I have felt calm and relaxed | -0.33 | -0.21 | -0.25 | -0.39 | -0.43 | -0.46 | -0.53 | -0.53 | -0.44 |
| WHO-5 | I have felt active and vigorous | -0.37 | -0.24 | -0.31 | -0.39 | -0.32 | -0.35 | -0.40 | -0.36 | -0.35 |
| WHO-5 | I woke up feeling fresh and rested | -0.33 | -0.18 | -0.31 | -0.46 | -0.32 | -0.41 | -0.42 | -0.41 | -0.36 |
| WHO-5 | My daily life has been filled with things that interest me | -0.31 | -0.20 | -0.22 | -0.36 | -0.41 | -0.40 | -0.46 | -0.41 | -0.40 |
| PROMIS-10 | In general, would you say your health is: | -0.39 | -0.24 | -0.43 | -0.41 | -0.25 | -0.30 | -0.33 | -0.32 | -0.28 |
| PROMIS-10 | In general, would you say your quality of life is: | -0.36 | -0.22 | -0.35 | -0.41 | -0.37 | -0.35 | -0.42 | -0.39 | -0.37 |
| PROMIS-10 | In general, how would rate your physical health? | -0.39 | -0.27 | -0.44 | -0.40 | -0.24 | -0.29 | -0.30 | -0.29 | -0.27 |
| PROMIS-10 | In general, how would rate your mental health, including you mood and ability to think? | -0.32 | -0.15 | -0.28 | -0.47 | -0.47 | -0.50 | -0.59 | -0.58 | -0.43 |
| PROMIS-10 | In general, how would rate your satisfaction with your social activities and relationships? | -0.31 | -0.18 | -0.29 | -0.39 | -0.46 | -0.39 | -0.47 | -0.44 | -0.38 |
| PROMIS-10 | To what extent are you able to carry out your everyday physical activities such as walking, climbing stairs, carrying groceries, or moving a chair? | -0.54 | -0.54 | -0.63 | -0.41 | -0.35 | -0.39 | -0.37 | -0.34 | -0.38 |
| PROMIS-10 | How would you rate your pain on average? | -0.39 | -0.24 | -0.31 | -0.55 | -0.58 | -0.60 | -0.72 | -0.73 | -0.54 |
| PROMIS-10 | How would you rate your fatigue on average? | -0.42 | -0.28 | -0.35 | -0.42 | -0.39 | -0.41 | -0.43 | -0.41 | -0.39 |
| PROMIS-10 | In general, please rate how well you carry out your usual social activities and roles. (This includes activities at home, at work and in your community, and responsibilities as a parent, child, spouse, employee, friend, etc.) | -0.55 | -0.53 | -0.44 | -0.33 | -0.26 | -0.30 | -0.28 | -0.26 | -0.31 |
| PROMIS-10 | How often have you been bothered by emotional problems such as feeling anxious, depressed or irritable? | -0.52 | -0.40 | -0.42 | -0.66 | -0.46 | -0.55 | -0.54 | -0.52 | -0.47 |
| ASCOT | Which of the following statements best describes how much control you have over your daily life? | 0.31 | 0.21 | 0.33 | 0.34 | 0.30 | 0.37 | 0.36 | 0.36 | 0.44 |
| ASCOT | Thinking about your personal care, by which we mean being clean and presentable in appearance, which of the following statements best describes your situation? | 0.40 | 0.33 | 0.29 | 0.36 | 0.37 | 0.41 | 0.41 | 0.40 | 0.41 |
| ASCOT | Thinking about the food and drink you get, which of the following statements best describes your situation? | 0.31 | 0.24 | 0.24 | 0.32 | 0.39 | 0.37 | 0.39 | 0.37 | 0.38 |
| ASCOT | Which of the following statements best describes how safe you feel? | 0.32 | 0.27 | 0.29 | 0.31 | 0.35 | 0.37 | 0.36 | 0.37 | 0.36 |
| ASCOT | Thinking about how much contact you’ve had with people you like, which of the following statements best describes your social situation? | 0.28 | 0.17 | 0.27 | 0.31 | 0.44 | 0.34 | 0.40 | 0.38 | 0.34 |
| ASCOT | Which of the following statements best describes how you spend your time? | 0.27 | 0.12 | 0.30 | 0.39 | 0.33 | 0.35 | 0.38 | 0.37 | 0.37 |
| ASCOT | Which of the following statements best describes how clean and comfortable your home is? | 0.33 | 0.22 | 0.27 | 0.34 | 0.31 | 0.35 | 0.36 | 0.36 | 0.35 |
| ASCOT | Which of these statements best describes how having help to do things makes you think and feel about yourself? | 0.16 | 0.10 | 0.24 | 0.20 | 0.12 | 0.16 | 0.16 | 0.16 | 0.13 |
| ASCOT | Thinking about the way you are helped and treated, and how that makes you think and feel about yourself, which of these statements best describes your situation? | 0.20 | 0.14 | 0.24 | 0.21 | 0.18 | 0.21 | 0.20 | 0.19 | 0.19 |
| OPQOL | Thinking about both the good and bad things that make up your quality of life, how would you rate the quality of your life as a whole? | -0.35 | -0.31 | -0.28 | -0.38 | -0.45 | -0.34 | -0.49 | -0.42 | -0.42 |
| OPQOL | I enjoy my life overall | -0.35 | -0.31 | -0.28 | -0.38 | -0.44 | -0.34 | -0.48 | -0.42 | -0.42 |
| OPQOL | I look forward to things | -0.31 | -0.30 | -0.29 | -0.36 | -0.40 | -0.33 | -0.42 | -0.37 | -0.35 |
| OPQOL | I am healthy enough to get out and about | -0.55 | -0.53 | -0.43 | -0.43 | -0.31 | -0.31 | -0.34 | -0.29 | -0.35 |
| OPQOL | My family, friends or neighbours would help me if needed | -0.18 | -0.15 | -0.15 | -0.20 | -0.26 | -0.18 | -0.19 | -0.20 | -0.21 |
| OPQOL | I have social or leisure activities/hobbies that I enjoy doing | -0.32 | -0.29 | -0.26 | -0.32 | -0.34 | -0.27 | -0.35 | -0.30 | -0.28 |
| OPQOL | I try to stay involved with things | -0.30 | -0.28 | -0.24 | -0.26 | -0.29 | -0.26 | -0.31 | -0.26 | -0.26 |
| OPQOL | I am healthy enough to have my independence | -0.50 | -0.50 | -0.38 | -0.38 | -0.25 | -0.30 | -0.29 | -0.26 | -0.35 |
| OPQOL | I can please myself what I do | -0.33 | -0.33 | -0.30 | -0.34 | -0.25 | -0.32 | -0.30 | -0.29 | -0.41 |
| OPQOL | I feel safe where I live | -0.25 | -0.25 | -0.22 | -0.28 | -0.28 | -0.23 | -0.28 | -0.26 | -0.24 |
| OPQOL | I get pleasure from my home | -0.27 | -0.27 | -0.20 | -0.29 | -0.30 | -0.24 | -0.31 | -0.28 | -0.33 |
| OPQOL | I take life as it comes and make the best of things | -0.19 | -0.19 | -0.17 | -0.26 | -0.32 | -0.29 | -0.40 | -0.37 | -0.31 |
| OPQOL | I feel lucky compared to most people | -0.27 | -0.22 | -0.23 | -0.28 | -0.29 | -0.24 | -0.34 | -0.30 | -0.33 |
| OPQOL | I have enough money to pay for household bills | -0.31 | -0.28 | -0.29 | -0.33 | -0.29 | -0.27 | -0.34 | -0.33 | -0.30 |
| PHQ-2 | Little interest or pleasure in doing things | 0.47 | 0.35 | 0.30 | 0.52 | 0.58 | 0.58 | 0.64 | 0.58 | 0.54 |
| PHQ-2 | Feeling down, depressed, or hopeless | 0.46 | 0.35 | 0.28 | 0.51 | 0.64 | 0.61 | 0.76 | 0.67 | 0.57 |
| GAD-2 | Feeling nervous, anxious, or on edge | 0.43 | 0.31 | 0.29 | 0.52 | 0.57 | 0.62 | 0.68 | 0.76 | 0.56 |
| GAD-2 | Not being able to stop or control worrying | 0.41 | 0.32 | 0.27 | 0.50 | 0.57 | 0.60 | 0.68 | 0.70 | 0.56 |
| s – strong correlation, ≥ 0.51; m – moderate correlation, 0.31-0.50; w – weak correlation, 0.11-0.30; and n – none, 0.00-0.10 | | | | | | | | | | |

**Appendix *12*: Known-groups validity - subgroup analysis (EQ-5D-5L and EQ-HWB-9 value sets in study population)**

|  | **Over 60** | | | | | | | |
| --- | --- | --- | --- | --- | --- | --- | --- | --- |
|  | **EQ-5D-5L** | | | | | | **EQ-HWB-9** | |
|  | **N** | **Australian** | **Canadian** | **New Zealand** | **United Kingdom** | **United States** | **N** | **UK pilot** |
|  |  | **value set** | **value set** | **value set** | **value set** | **value set** |  | **value set** |
| **Physical disability** |  |  |  |  |  |  |  |  |
| No | 953 | 0.942 (0.07) | 0.886 (0.07) | 0.904 (0.10) | 0.908 (0.09) | 0.902 (0.11) | 949 | 0.881 (0.12) |
| Yes | 878 | 0.773 (0.21) | 0.716 (0.18) | 0.650 (0.25) | 0.709 (0.20) | 0.637 (0.25) | 872 | 0.691 (0.22) |
|  |  | z = 26.7 (25.4, 27.9) | z = 27.1 (25.9, 28.3) | z = 26.8 (25.5, 28) | z = 26.5 (25.2, 27.8) | z = 27.7 (26.5, 28.9) |  | z = 22.9 (21.5, 24.4) |
|  |  | d = 1.59 (1.47, 1.71) | d = 1.63 (1.51, 1.75) | d = 1.6 (1.48, 1.73) | d = 1.58 (1.45, 1.7) | d = 1.7 (1.57, 1.82) |  | d = 1.27 (1.16, 1.39) |
|  |  | ES = L | ES = L | ES = L | ES = L | ES = L |  | ES = L |
| **Mental disability** |  |  |  |  |  |  |  |  |
| No | 1,476 | 0.896 (0.13) | 0.839 (0.13) | 0.833 (0.18) | 0.854 (0.14) | 0.825 (0.19) | 1,467 | 0.84 (0.15) |
| Yes | 314 | 0.703 (0.25) | 0.652 (0.21) | 0.565 (0.28) | 0.636 (0.23) | 0.557 (0.29) | 314 | 0.576 (0.26) |
|  |  | z = 17.3 (15.8, 18.8) | z = 17.3 (15.8, 18.9) | z = 18 (16.5, 19.4) | z = 18 (16.4, 19.6) | z = 17.1 (15.5, 18.7) |  | z = 18.6 (17.2, 20.1) |
|  |  | d = 0.89 (0.8, 0.99) | d = 0.9 (0.8, 0.99) | d = 0.94 (0.84, 1.03) | d = 0.94 (0.84, 1.04) | d = 0.89 (0.79, 0.99) |  | d = 0.98 (0.89, 1.08) |
|  |  | ES = L | ES = L | ES = L | ES = L | ES = M to L |  | ES = L |
| **Chronic conditions** |  |  |  |  |  |  |  |  |
| 0 to 1 health condition | 568 | 0.940 (0.09) | 0.885 (0.09) | 0.903 (0.13) | 0.908 (0.11) | 0.899 (0.13) | 567 | 0.888 (0.12) |
| 2 or more health conditions | 1,301 | 0.825 (0.19) | 0.767 (0.17) | 0.727 (0.24) | 0.769 (0.19) | 0.718 (0.25) | 1,294 | 0.743 (0.22) |
|  |  | z = 18 (16.3, 19.6) | z = 18.3 (16.7, 19.9) | z = 18.1 (16.5, 19.7) | z = 17.9 (16.3, 19.6) | z = 18.5 (16.9, 20) |  | z = 16.4 (14.7, 18) |
|  |  | d = 0.91 (0.81, 1.01) | d = 0.94 (0.84, 1.03) | d = 0.92 (0.82, 1.02) | d = 0.91 (0.81, 1.01) | d = 0.94 (0.85, 1.04) |  | d = 0.82 (0.72, 0.92) |
|  |  | ES = L | ES = L | ES = L | ES = L | ES = L |  | ES = M to L |
| **Sleep quality** |  |  |  |  |  |  |  |  |
| Good to very good | 799 | 0.915 (0.11) | 0.858 (0.11) | 0.862 (0.16) | 0.877 (0.13) | 0.855 (0.16) | 793 | 0.867 (0.14) |
| Very poor to fair | 1,065 | 0.818 (0.20) | 0.762 (0.18) | 0.721 (0.25) | 0.763 (0.20) | 0.712 (0.26) | 1,065 | 0.728 (0.23) |
|  |  | z = 13.7 (11.9, 15.5) | z = 14.1 (12.3, 15.8) | z = 14.5 (12.7, 16.2) | z = 14.4 (12.6, 16.2) | z = 14.1 (12.4, 15.9) |  | z = 16.5 (14.9, 18.2) |
|  |  | d = 0.6 (0.53, 0.68) | d = 0.62 (0.55, 0.69) | d = 0.64 (0.57, 0.71) | d = 0.63 (0.56, 0.7) | d = 0.62 (0.55, 0.69) |  | d = 0.72 (0.65, 0.78) |
|  |  | ES = M | ES = M | ES = M | ES = M | ES = M |  | ES = M |
| **Social support** |  |  |  |  |  |  |  |  |
| Good to excellent | 1,361 | 0.884 (0.16) | 0.828 (0.14) | 0.817 (0.20) | 0.840 (0.16) | 0.809 (0.21) | 1,352 | 0.829 (0.17) |
| Poor to fair | 498 | 0.794 (0.21) | 0.737 (0.19) | 0.683 (0.26) | 0.733 (0.21) | 0.676 (0.27) | 499 | 0.675 (0.24) |
|  |  | z = 10.9 (9.1, 12.8) | z = 11.1 (9.2, 12.9) | z = 11.7 (9.9, 13.5) | z = 11.7 (9.8, 13.5) | z = 10.9 (9, 12.9) |  | z = 14.4 (12.6, 16.2) |
|  |  | d = 0.49 (0.41, 0.57) | d = 0.5 (0.42, 0.58) | d = 0.52 (0.45, 0.6) | d = 0.52 (0.45, 0.6) | d = 0.49 (0.41, 0.57) |  | d = 0.64 (0.56, 0.71) |
|  |  | ES = S to M | ES = S to M | ES = S to M | ES = S to M | ES = S to M |  | ES = M |
| **Time for caring** |  |  |  |  |  |  |  |  |
| Always/sometimes enough time | 1,192 | 0.874 (0.16) | 0.817 (0.15) | 0.801 (0.21) | 0.828 (0.17) | 0.794 (0.22) | 1,190 | 0.812 (0.18) |
| Never enough time | 668 | 0.835 (0.20) | 0.778 (0.18) | 0.745 (0.25) | 0.781 (0.20) | 0.737 (0.26) | 662 | 0.743 (0.23) |
|  |  | z = 4.6 (2.7, 6.6) | z = 4.7 (2.7, 6.6) | z = 4.8 (2.7, 6.8) | z = 4.9 (2.9, 6.9) | z = 4.6 (2.7, 6.6) |  | z = 6.6 (4.7, 8.5) |
|  |  | d = 0.21 (0.12, 0.31) | d = 0.21 (0.13, 0.3) | d = 0.22 (0.12, 0.31) | d = 0.23 (0.14, 0.32) | d = 0.21 (0.12, 0.3) |  | d = 0.3 (0.22, 0.39) |
|  |  | ES = Nil to S | ES = Nil to S | ES = Nil to S | ES = Nil to S | ES = Nil to S |  | ES = S |
| **Impact of caring on own health** |  |  |  |  |  |  |  |  |
| No impact | 779 | 0.903 (0.14) | 0.847 (0.13) | 0.847 (0.18) | 0.866 (0.15) | 0.840 (0.19) | 788 | 0.860 (0.15) |
| Impacts | 1,090 | 0.829 (0.19) | 0.771 (0.18) | 0.733 (0.24) | 0.772 (0.19) | 0.726 (0.25) | 1,073 | 0.734 (0.22) |
|  |  | z = 11.4 (9.5, 13.3) | z = 11.6 (9.7, 13.5) | z = 12.5 (10.6, 14.4) | z = 12.6 (10.8, 14.4) | z = 11.5 (9.7, 13.4) |  | z = 15.4 (13.6, 17.1) |
|  |  | d = 0.55 (0.45, 0.64) | d = 0.55 (0.46, 0.65) | d = 0.6 (0.5, 0.71) | d = 0.61 (0.51, 0.7) | d = 0.55 (0.46, 0.65) |  | d = 0.76 (0.66, 0.86) |
|  |  | ES = S to M | ES = S to M | ES = M | ES = M | ES = S to M |  | ES = M to L |
| **Psychological wellbeing** |  |  |  |  |  |  |  |  |
| Good mental wellbeing | 767 | 0.909 (0.11) | 0.851 (0.11) | 0.852 (0.16) | 0.868 (0.12) | 0.845 (0.16) | 770 | 0.866 (0.12) |
| Poor mental wellbeing | 1,102 | 0.826 (0.20) | 0.769 (0.18) | 0.731 (0.25) | 0.772 (0.21) | 0.723 (0.26) | 1,091 | 0.732 (0.23) |
|  |  | z = 10 (8.1, 11.9) | z = 10.4 (8.5, 12.2) | z = 11.1 (9.3, 12.9) | z = 11 (9.2, 12.8) | z = 10.6 (8.8, 12.4) |  | z = 13.8 (12, 15.5) |
|  |  | d = 0.48 (0.38, 0.57) | d = 0.49 (0.4, 0.59) | d = 0.53 (0.44, 0.63) | d = 0.53 (0.43, 0.62) | d = 0.51 (0.41, 0.6) |  | d = 0.67 (0.58, 0.77) |
|  |  | ES = S to M | ES = S to M | ES = S to M | ES = S to M | ES = S to M |  | ES = M |
| **Self-reported quality of life** |  |  |  |  |  |  |  |  |
| Excellent/Very good | 855 | 0.933 (0.09) | 0.877 (0.09) | 0.893 (0.13) | 0.900 (0.11) | 0.885 (0.13) | 854 | 0.890 (0.12) |
| Good | 663 | 0.849 (0.15) | 0.788 (0.14) | 0.753 (0.20) | 0.789 (0.16) | 0.746 (0.20) | 659 | 0.778 (0.16) |
| Poor/Fair | 347 | 0.704 (0.25) | 0.653 (0.21) | 0.563 (0.28) | 0.639 (0.23) | 0.554 (0.29) | 345 | 0.553 (0.25) |
|  |  | KW = 512 (447, 578) | KW = 537 (469, 605) | KW = 584 (518, 651) | KW = 580 (511, 649) | KW = 543 (476, 610) |  | KW = 684 (620, 747) |
|  |  | d = 1.23 (1.12, 1.34) | d = 1.27 (1.16, 1.38) | d = 1.35 (1.24, 1.46) | d = 1.34 (1.23, 1.46) | d = 1.28 (1.17, 1.39) |  | d = 1.52 (1.41, 1.64) |
|  |  | ES = L | ES = L | ES = L | ES = L | ES = L |  | ES = L |
|  |  |  |  |  |  |  |  |  |
|  | **Under 60** | | | | | | | |
|  | **EQ-5D-5L** | | | | | | **EQ-HWB-9** | |
|  | **N** | **Australian** | **Canadian** | **New Zealand** | **United Kingdom** | **United States** | **N** | **UK pilot** |
|  |  | **value set** | **value set** | **value set** | **value set** | **value set** |  | **value set** |
| **Physical disability** |  |  |  |  |  |  |  |  |
| No | 3,111 | 0.902 (0.14) | 0.848 (0.13) | 0.845 (0.18) | 0.863 (0.14) | 0.849 (0.18) | 3,078 | 0.761 (0.20) |
| Yes | 1,448 | 0.673 (0.31) | 0.634 (0.26) | 0.538 (0.36) | 0.621 (0.28) | 0.534 (0.35) | 1,446 | 0.504 (0.29) |
|  |  | z = 32.2 (30.6, 33.8) | z = 32.9 (31.2, 34.5) | z = 32.8 (31.3, 34.4) | z = 32.6 (30.9, 34.3) | z = 33.6 (32, 35.2) |  | z = 30.1 (28.6, 31.7) |
|  |  | d = 1.08 (1.01, 1.15) | d = 1.11 (1.04, 1.19) | d = 1.11 (1.04, 1.18) | d = 1.1 (1.03, 1.18) | d = 1.15 (1.08, 1.22) |  | d = 1 (0.94, 1.07) |
|  |  | ES = L | ES = L | ES = L | ES = L | ES = L |  | ES = L |
| **Mental disability** |  |  |  |  |  |  |  |  |
| No | 2,732 | 0.913 (0.14) | 0.857 (0.13) | 0.859 (0.18) | 0.875 (0.15) | 0.86 (0.19) | 2,705 | 0.788 (0.19) |
| Yes | 1,733 | 0.692 (0.29) | 0.653 (0.24) | 0.565 (0.33) | 0.642 (0.26) | 0.567 (0.33) | 1,725 | 0.501 (0.27) |
|  |  | z = 35.5 (33.9, 37.1) | z = 36 (34.5, 37.4) | z = 37 (35.6, 38.4) | z = 36.3 (34.8, 37.8) | z = 36.4 (35, 37.9) |  | z = 36.3 (34.9, 37.7) |
|  |  | d = 1.25 (1.18, 1.33) | d = 1.28 (1.2, 1.35) | d = 1.33 (1.26, 1.4) | d = 1.29 (1.22, 1.37) | d = 1.3 (1.23, 1.37) |  | d = 1.3 (1.23, 1.37) |
|  |  | ES = L | ES = L | ES = L | ES = L | ES = L |  | ES = L |
| **Chronic conditions** |  |  |  |  |  |  |  |  |
| 0 to 1 health condition | 2,233 | 0.914 (0.16) | 0.860 (0.14) | 0.863 (0.21) | 0.879 (0.16) | 0.864 (0.20) | 2,210 | 0.778 (0.21) |
| 2 or more health conditions | 2,513 | 0.749 (0.26) | 0.704 (0.22) | 0.638 (0.31) | 0.698 (0.24) | 0.640 (0.30) | 2,499 | 0.584 (0.27) |
|  |  | z = 33.3 (31.8, 34.9) | z = 33.5 (31.9, 35) | z = 33.8 (32.3, 35.3) | z = 33.6 (32, 35.1) | z = 33.4 (31.8, 35) |  | z = 27.6 (26, 29.3) |
|  |  | d = 1.11 (1.04, 1.18) | d = 1.11 (1.04, 1.18) | d = 1.13 (1.06, 1.19) | d = 1.12 (1.05, 1.18) | d = 1.11 (1.04, 1.18) |  | d = 0.88 (0.82, 0.94) |
|  |  | ES = L | ES = L | ES = L | ES = L | ES = L |  | ES = L |
| **Sleep quality** |  |  |  |  |  |  |  |  |
| Good to very good | 1,727 | 0.870 (0.24) | 0.820 (0.21) | 0.804 (0.31) | 0.834 (0.24) | 0.806 (0.30) | 1,717 | 0.735 (0.27) |
| Very poor to fair | 3,011 | 0.803 (0.23) | 0.754 (0.20) | 0.710 (0.27) | 0.754 (0.21) | 0.711 (0.27) | 2,986 | 0.641 (0.25) |
|  |  | z = 19.2 (17.3, 21) | z = 18.8 (16.9, 20.7) | z = 19 (17.1, 21) | z = 18.8 (16.9, 20.7) | z = 18.3 (16.4, 20.2) |  | z = 16.5 (14.6, 18.4) |
|  |  | d = 0.54 (0.49, 0.59) | d = 0.53 (0.48, 0.58) | d = 0.53 (0.48, 0.58) | d = 0.53 (0.48, 0.58) | d = 0.51 (0.46, 0.56) |  | d = 0.47 (0.42, 0.52) |
|  |  | ES = S to M | ES = S to M | ES = S to M | ES = S to M | ES = S to M |  | ES = S to M |
| **Social support** |  |  |  |  |  |  |  |  |
| Good to excellent | 3,333 | 0.852 (0.22) | 0.802 (0.19) | 0.777 (0.28) | 0.811 (0.22) | 0.779 (0.27) | 3,312 | 0.716 (0.25) |
| Poor to fair | 1,367 | 0.768 (0.25) | 0.722 (0.21) | 0.668 (0.28) | 0.719 (0.23) | 0.668 (0.29) | 1,354 | 0.579 (0.27) |
|  |  | z = 16.1 (14.3, 17.9) | z = 15.9 (14.1, 17.7) | z = 16.5 (14.6, 18.3) | z = 16.3 (14.5, 18.1) | z = 15.7 (13.9, 17.6) |  | z = 17.6 (15.9, 19.4) |
|  |  | d = 0.46 (0.41, 0.51) | d = 0.45 (0.4, 0.5) | d = 0.47 (0.42, 0.52) | d = 0.46 (0.41, 0.51) | d = 0.45 (0.4, 0.5) |  | d = 0.5 (0.45, 0.55) |
|  |  | ES = S to M | ES = S to M | ES = S to M | ES = S to M | ES = S |  | ES = S to M |
| **Time for caring** |  |  |  |  |  |  |  |  |
| Always enough time | 2,037 | 0.831 (0.25) | 0.785 (0.22) | 0.755 (0.31) | 0.794 (0.24) | 0.758 (0.30) | 2,031 | 0.681 (0.28) |
| Never/sometimes enough time | 2,687 | 0.824 (0.22) | 0.772 (0.19) | 0.736 (0.27) | 0.775 (0.21) | 0.736 (0.27) | 2,657 | 0.671 (0.25) |
|  |  | z = 5.9 (3.9, 7.9) | z = 6 (4, 8) | z = 6.2 (4.3, 8.1) | z = 6.5 (4.6, 8.5) | z = 6.2 (4.2, 8.2) |  | z = 3.8 (1.8, 5.8) |
|  |  | d = 0.17 (0.11, 0.23) | d = 0.17 (0.12, 0.23) | d = 0.18 (0.13, 0.24) | d = 0.19 (0.13, 0.24) | d = 0.18 (0.12, 0.24) |  | d = 0.11 (0.05, 0.17) |
|  |  | ES = Nil to S | ES = Nil to S | ES = Nil to S | ES = Nil to S | ES = Nil to S |  | ES = Nil |
| **Impact of caring on own health** |  |  |  |  |  |  |  |  |
| No impact | 972 | 0.921 (0.14) | 0.868 (0.13) | 0.876 (0.18) | 0.891 (0.15) | 0.878 (0.18) | 979 | 0.831 (0.20) |
| Impacts | 3,774 | 0.803 (0.25) | 0.754 (0.21) | 0.710 (0.30) | 0.756 (0.23) | 0.711 (0.30) | 3,730 | 0.634 (0.26) |
|  |  | z = 19.2 (17.4, 20.9) | z = 19.6 (17.8, 21.3) | z = 20.2 (18.4, 21.9) | z = 20.2 (18.5, 22) | z = 20 (18.3, 21.7) |  | z = 25 (23.1, 26.8) |
|  |  | d = 0.58 (0.52, 0.64) | d = 0.59 (0.54, 0.65) | d = 0.61 (0.55, 0.67) | d = 0.61 (0.56, 0.67) | d = 0.61 (0.55, 0.66) |  | d = 0.78 (0.71, 0.85) |
|  |  | ES = M | ES = M | ES = M | ES = M | ES = M |  | ES = M to L |
| **Psychological wellbeing** |  |  |  |  |  |  |  |  |
| Good mental wellbeing | 1,206 | 0.865 (0.24) | 0.816 (0.20) | 0.799 (0.30) | 0.830 (0.23) | 0.803 (0.29) | 1,201 | 0.748 (0.27) |
| Poor mental wellbeing | 3,540 | 0.814 (0.23) | 0.764 (0.20) | 0.725 (0.28) | 0.767 (0.22) | 0.726 (0.28) | 3,508 | 0.651 (0.26) |
|  |  | z = 11.4 (9.5, 13.3) | z = 11.7 (9.8, 13.5) | z = 12.5 (10.5, 14.4) | z = 12.3 (10.4, 14.2) | z = 11.9 (10, 13.9) |  | z = 14.8 (12.8, 16.7) |
|  |  | d = 0.34 (0.28, 0.39) | d = 0.34 (0.29, 0.4) | d = 0.37 (0.31, 0.43) | d = 0.36 (0.31, 0.42) | d = 0.35 (0.29, 0.41) |  | d = 0.44 (0.38, 0.5) |
|  |  | ES = S | ES = S | ES = S | ES = S | ES = S |  | ES = S to M |
| **Self-reported quality of life** |  |  |  |  |  |  |  |  |
| Excellent/Very good | 2,080 | 0.885 (0.21) | 0.834 (0.18) | 0.824 (0.27) | 0.849 (0.21) | 0.827 (0.26) | 2,062 | 0.760 (0.24) |
| Good | 1,541 | 0.850 (0.18) | 0.793 (0.16) | 0.762 (0.22) | 0.796 (0.18) | 0.764 (0.23) | 1,535 | 0.696 (0.21) |
| Poor/Fair | 1,110 | 0.686 (0.28) | 0.649 (0.23) | 0.567 (0.31) | 0.641 (0.25) | 0.566 (0.32) | 1,101 | 0.486 (0.27) |
|  |  | KW = 813 (717, 910) | KW = 814 (717, 910) | KW = 856 (761, 952) | KW = 826 (729, 924) | KW = 812 (717, 906) |  | KW = 904 (808, 1000) |
|  |  | d = 0.91 (0.85, 0.98) | d = 0.91 (0.85, 0.98) | d = 0.94 (0.88, 1) | d = 0.92 (0.85, 0.98) | d = 0.91 (0.85, 0.97) |  | d = 0.98 (0.91, 1.04) |
|  |  | ES = L | ES = L | ES = L | ES = L | ES = L |  | ES = L |
|  |  |  |  |  |  |  |  |  |
|  | **Coping financially** | | | | | | | |
|  | **EQ-5D-5L** | | | | | | **EQ-HWB-9** | |
|  | **N** | **Australian** | **Canadian** | **New Zealand** | **United Kingdom** | **United States** | **N** | **UK pilot** |
|  |  | **value set** | **value set** | **value set** | **value set** | **value set** |  | **value set** |
| **Physical disability** |  |  |  |  |  |  |  |  |
| No | 2,712 | 0.930 (0.11) | 0.874 (0.10) | 0.885 (0.15) | 0.895 (0.12) | 0.885 (0.15) | 2,691 | 0.823 (0.17) |
| Yes | 1,355 | 0.756 (0.27) | 0.707 (0.23) | 0.636 (0.33) | 0.700 (0.25) | 0.630 (0.31) | 1,348 | 0.624 (0.27) |
|  |  | z = 31.1 (29.5, 32.7) | z = 31.5 (30, 33.1) | z = 31 (29.4, 32.5) | z = 30.7 (29.1, 32.4) | z = 32.1 (30.6, 33.7) |  | z = 26.5 (24.8, 28.1) |
|  |  | d = 1.12 (1.04, 1.19) | d = 1.14 (1.07, 1.21) | d = 1.11 (1.04, 1.18) | d = 1.1 (1.02, 1.18) | d = 1.17 (1.09, 1.24) |  | d = 0.92 (0.85, 0.99) |
|  |  | ES = L | ES = L | ES = L | ES = L | ES = L |  | ES = L |
| **Mental disability** |  |  |  |  |  |  |  |  |
| No | 2,949 | 0.920 (0.12) | 0.864 (0.12) | 0.870 (0.17) | 0.884 (0.14) | 0.867 (0.17) | 2,930 | 0.827 (0.17) |
| Yes | 1,056 | 0.734 (0.29) | 0.688 (0.24) | 0.609 (0.34) | 0.677 (0.26) | 0.611 (0.33) | 1,049 | 0.557 (0.27) |
|  |  | z = 27.4 (25.7, 29) | z = 27.9 (26.2, 29.6) | z = 28.9 (27.2, 30.6) | z = 28.5 (26.8, 30.1) | z = 28.1 (26.5, 29.7) |  | z = 31.4 (29.9, 32.9) |
|  |  | d = 0.96 (0.89, 1.03) | d = 0.98 (0.91, 1.06) | d = 1.03 (0.95, 1.1) | d = 1.01 (0.93, 1.08) | d = 0.99 (0.92, 1.06) |  | d = 1.15 (1.08, 1.22) |
|  |  | ES = L | ES = L | ES = L | ES = L | ES = L |  | ES = L |
| **Chronic conditions** |  |  |  |  |  |  |  |  |
| 0 to 1 health condition | 2,012 | 0.926 (0.14) | 0.872 (0.13) | 0.881 (0.20) | 0.894 (0.16) | 0.881 (0.19) | 1,995 | 0.813 (0.20) |
| 2 or more health conditions | 2,166 | 0.820 (0.22) | 0.767 (0.19) | 0.726 (0.27) | 0.767 (0.21) | 0.723 (0.27) | 2,157 | 0.701 (0.24) |
|  |  | z = 26.9 (25.3, 28.6) | z = 26.9 (25.3, 28.6) | z = 26.5 (24.8, 28.2) | z = 26.4 (24.7, 28.2) | z = 26.9 (25.2, 28.6) |  | z = 18.2 (16.4, 20) |
|  |  | d = 0.92 (0.85, 0.99) | d = 0.92 (0.85, 0.99) | d = 0.9 (0.83, 0.97) | d = 0.9 (0.82, 0.97) | d = 0.92 (0.85, 0.98) |  | d = 0.59 (0.53, 0.65) |
|  |  | ES = L | ES = L | ES = L | ES = L | ES = L |  | ES = M |
| **Sleep quality** |  |  |  |  |  |  |  |  |
| Good to very good | 1,972 | 0.893 (0.21) | 0.841 (0.18) | 0.835 (0.27) | 0.858 (0.21) | 0.834 (0.26) | 1,961 | 0.785 (0.25) |
| Very poor to fair | 2,199 | 0.852 (0.18) | 0.797 (0.16) | 0.770 (0.23) | 0.802 (0.18) | 0.768 (0.23) | 2,186 | 0.728 (0.21) |
|  |  | z = 15.8 (14, 17.7) | z = 15.8 (13.9, 17.7) | z = 16.2 (14.4, 18.1) | z = 16.1 (14.2, 18) | z = 15.4 (13.5, 17.2) |  | z = 14.6 (12.7, 16.5) |
|  |  | d = 0.48 (0.42, 0.53) | d = 0.47 (0.42, 0.53) | d = 0.49 (0.43, 0.54) | d = 0.48 (0.43, 0.54) | d = 0.46 (0.41, 0.52) |  | d = 0.44 (0.39, 0.5) |
|  |  | ES = S to M | ES = S to M | ES = S to M | ES = S to M | ES = S to M |  | ES = S |
| **Social support** |  |  |  |  |  |  |  |  |
| Good to excellent | 3,323 | 0.880 (0.20) | 0.827 (0.17) | 0.815 (0.25) | 0.840 (0.20) | 0.813 (0.25) | 3,305 | 0.770 (0.23) |
| Poor to fair | 816 | 0.837 (0.19) | 0.781 (0.17) | 0.748 (0.23) | 0.784 (0.19) | 0.747 (0.24) | 810 | 0.694 (0.23) |
|  |  | z = 10.6 (8.8, 12.5) | z = 10.4 (8.6, 12.3) | z = 11.3 (9.5, 13.1) | z = 11.2 (9.4, 13) | z = 10.2 (8.4, 12.1) |  | z = 11.2 (9.5, 12.9) |
|  |  | d = 0.33 (0.27, 0.38) | d = 0.32 (0.27, 0.38) | d = 0.35 (0.29, 0.4) | d = 0.34 (0.29, 0.4) | d = 0.31 (0.26, 0.37) |  | d = 0.34 (0.29, 0.4) |
|  |  | ES = S | ES = S | ES = S | ES = S | ES = S |  | ES = S |
| **Time for caring** |  |  |  |  |  |  |  |  |
| Always enough time | 2,193 | 0.871 (0.21) | 0.821 (0.18) | 0.806 (0.27) | 0.835 (0.21) | 0.806 (0.26) | 2,191 | 0.764 (0.25) |
| Never/sometimes enough time | 1,964 | 0.871 (0.18) | 0.814 (0.16) | 0.795 (0.23) | 0.822 (0.18) | 0.793 (0.23) | 1,941 | 0.745 (0.21) |
|  |  | z = 4.5 (2.5, 6.4) | z = 4.6 (2.7, 6.6) | z = 5.3 (3.4, 7.3) | z = 5.6 (3.7, 7.6) | z = 4.8 (2.8, 6.8) |  | z = 6.6 (4.7, 8.6) |
|  |  | d = 0.14 (0.08, 0.2) | d = 0.14 (0.08, 0.2) | d = 0.16 (0.1, 0.22) | d = 0.17 (0.11, 0.23) | d = 0.15 (0.09, 0.21) |  | d = 0.21 (0.15, 0.26) |
|  |  | ES = Nil | ES = Nil to S | ES = Nil to S | ES = Nil to S | ES = Nil to S |  | ES = Nil to S |
| **Impact of caring on own health** |  |  |  |  |  |  |  |  |
| No impact | 1,321 | 0.929 (0.11) | 0.875 (0.11) | 0.886 (0.16) | 0.899 (0.13) | 0.883 (0.16) | 1,334 | 0.874 (0.14) |
| Impacts | 2,857 | 0.844 (0.22) | 0.791 (0.19) | 0.761 (0.28) | 0.796 (0.22) | 0.760 (0.27) | 2,818 | 0.698 (0.24) |
|  |  | z = 15.7 (13.9, 17.6) | z = 16.2 (14.5, 18) | z = 17.6 (15.8, 19.4) | z = 17.8 (16.1, 19.6) | z = 16.4 (14.6, 18.3) |  | z = 27.5 (25.8, 29.2) |
|  |  | d = 0.5 (0.44, 0.56) | d = 0.52 (0.46, 0.58) | d = 0.56 (0.5, 0.63) | d = 0.57 (0.51, 0.63) | d = 0.53 (0.46, 0.59) |  | d = 0.94 (0.87, 1.01) |
|  |  | ES = S to M | ES = S to M | ES = M | ES = M | ES = S to M |  | ES = L |
| **Psychological wellbeing** |  |  |  |  |  |  |  |  |
| Good mental wellbeing | 1,495 | 0.888 (0.20) | 0.836 (0.17) | 0.829 (0.26) | 0.853 (0.20) | 0.829 (0.25) | 1,501 | 0.798 (0.23) |
| Poor mental wellbeing | 2,683 | 0.861 (0.19) | 0.807 (0.17) | 0.785 (0.24) | 0.815 (0.20) | 0.782 (0.25) | 2,651 | 0.730 (0.22) |
|  |  | z = 7.2 (5.3, 9) | z = 7.5 (5.6, 9.4) | z = 8.7 (6.8, 10.6) | z = 8.7 (6.8, 10.6) | z = 7.7 (5.7, 9.7) |  | z = 13.3 (11.3, 15.2) |
|  |  | d = 0.22 (0.16, 0.28) | d = 0.23 (0.17, 0.3) | d = 0.27 (0.21, 0.33) | d = 0.27 (0.21, 0.33) | d = 0.24 (0.18, 0.3) |  | d = 0.42 (0.36, 0.49) |
|  |  | ES = Nil to S | ES = Nil to S | ES = S | ES = S | ES = Nil to S |  | ES = S |
| **Self-reported quality of life** |  |  |  |  |  |  |  |  |
| Excellent/Very good | 2,390 | 0.903 (0.19) | 0.851 (0.16) | 0.851 (0.24) | 0.869 (0.19) | 0.850 (0.24) | 2,378 | 0.802 (0.23) |
| Good | 1,283 | 0.866 (0.16) | 0.806 (0.15) | 0.780 (0.21) | 0.810 (0.17) | 0.778 (0.21) | 1,280 | 0.744 (0.19) |
| Poor/Fair | 494 | 0.733 (0.25) | 0.686 (0.21) | 0.612 (0.28) | 0.679 (0.23) | 0.610 (0.29) | 487 | 0.554 (0.24) |
|  |  | KW = 541 (458, 623) | KW = 549 (471, 628) | KW = 595 (511, 680) | KW = 578 (492, 664) | KW = 547 (465, 629) |  | KW = 620 (542, 698) |
|  |  | d = 0.77 (0.7, 0.84) | d = 0.78 (0.71, 0.84) | d = 0.82 (0.75, 0.88) | d = 0.8 (0.73, 0.87) | d = 0.78 (0.71, 0.84) |  | d = 0.84 (0.78, 0.9) |
|  |  | ES = M to L | ES = M to L | ES = M to L | ES = M to L | ES = M to L |  | ES = M to L |
|  |  |  |  |  |  |  |  |  |
|  | **Financially strained** | | | | | | | |
|  | **EQ-5D-5L** | | | | | | **EQ-HWB-9** | |
|  | **N** | **Australian** | **Canadian** | **New Zealand** | **United Kingdom** | **United States** | **N** | **UK pilot** |
|  |  | **value set** | **value set** | **value set** | **value set** | **value set** |  | **value set** |
| **Physical disability** |  |  |  |  |  |  |  |  |
| No | 1,337 | 0.875 (0.15) | 0.821 (0.13) | 0.807 (0.19) | 0.831 (0.15) | 0.813 (0.18) | 1,321 | 0.720 (0.21) |
| Yes | 962 | 0.646 (0.28) | 0.605 (0.23) | 0.502 (0.31) | 0.590 (0.25) | 0.491 (0.31) | 961 | 0.504 (0.28) |
|  |  | z = 25.7 (24.3, 27.1) | z = 26.3 (24.9, 27.8) | z = 26.6 (25.1, 28) | z = 26.2 (24.8, 27.7) | z = 27.4 (26.1, 28.8) |  | z = 19 (17.3, 20.7) |
|  |  | d = 1.27 (1.17, 1.37) | d = 1.31 (1.21, 1.42) | d = 1.33 (1.23, 1.43) | d = 1.31 (1.2, 1.41) | d = 1.39 (1.29, 1.5) |  | d = 0.87 (0.78, 0.96) |
|  |  | ES = L | ES = L | ES = L | ES = L | ES = L |  | ES = M to L |
| **Mental disability** |  |  |  |  |  |  |  |  |
| No | 1,245 | 0.877 (0.16) | 0.820 (0.15) | 0.803 (0.20) | 0.830 (0.16) | 0.803 (0.21) | 1,228 | 0.758 (0.19) |
| Yes | 982 | 0.650 (0.27) | 0.614 (0.23) | 0.517 (0.30) | 0.601 (0.24) | 0.516 (0.30) | 981 | 0.465 (0.27) |
|  |  | z = 25.1 (23.7, 26.5) | z = 25.2 (23.7, 26.7) | z = 26.1 (24.7, 27.5) | z = 25.7 (24.3, 27.1) | z = 25.3 (23.9, 26.7) |  | z = 26.4 (25, 27.8) |
|  |  | d = 1.26 (1.16, 1.36) | d = 1.26 (1.16, 1.37) | d = 1.33 (1.23, 1.43) | d = 1.3 (1.2, 1.4) | d = 1.27 (1.17, 1.37) |  | d = 1.36 (1.25, 1.46) |
|  |  | ES = L | ES = L | ES = L | ES = L | ES = L |  | ES = L |
| **Chronic conditions** |  |  |  |  |  |  |  |  |
| 0 to 1 health condition | 776 | 0.902 (0.15) | 0.848 (0.13) | 0.846 (0.19) | 0.863 (0.15) | 0.849 (0.19) | 769 | 0.770 (0.19) |
| 2 or more health conditions | 1,635 | 0.715 (0.26) | 0.671 (0.22) | 0.593 (0.29) | 0.662 (0.23) | 0.591 (0.30) | 1,623 | 0.556 (0.27) |
|  |  | z = 23.7 (22.1, 25.4) | z = 24.2 (22.6, 25.8) | z = 24.7 (23.2, 26.2) | z = 24.3 (22.7, 25.8) | z = 24.5 (23, 26) |  | z = 20 (18.4, 21.6) |
|  |  | d = 1.11 (1.01, 1.2) | d = 1.13 (1.03, 1.23) | d = 1.16 (1.07, 1.26) | d = 1.14 (1.04, 1.23) | d = 1.15 (1.06, 1.24) |  | d = 0.9 (0.81, 0.98) |
|  |  | ES = L | ES = L | ES = L | ES = L | ES = L |  | ES = L |
| **Sleep quality** |  |  |  |  |  |  |  |  |
| Good to very good | 544 | 0.853 (0.21) | 0.800 (0.19) | 0.774 (0.27) | 0.809 (0.21) | 0.776 (0.26) | 539 | 0.749 (0.24) |
| Very poor to fair | 1,861 | 0.754 (0.25) | 0.707 (0.21) | 0.646 (0.28) | 0.703 (0.23) | 0.645 (0.29) | 1,849 | 0.589 (0.26) |
|  |  | z = 11.6 (9.8, 13.5) | z = 11.6 (9.7, 13.5) | z = 11.9 (10.1, 13.8) | z = 11.8 (9.9, 13.6) | z = 11.4 (9.6, 13.3) |  | z = 14.5 (12.6, 16.4) |
|  |  | d = 0.46 (0.39, 0.53) | d = 0.46 (0.39, 0.53) | d = 0.47 (0.4, 0.54) | d = 0.47 (0.4, 0.54) | d = 0.45 (0.38, 0.53) |  | d = 0.57 (0.5, 0.64) |
|  |  | ES = S to M | ES = S to M | ES = S to M | ES = S to M | ES = S to M |  | ES = S to M |
| **Social support** |  |  |  |  |  |  |  |  |
| Good to excellent | 1,354 | 0.816 (0.22) | 0.765 (0.19) | 0.725 (0.27) | 0.768 (0.21) | 0.726 (0.27) | 1,342 | 0.696 (0.24) |
| Poor to fair | 1,043 | 0.726 (0.26) | 0.682 (0.22) | 0.612 (0.29) | 0.675 (0.24) | 0.610 (0.30) | 1,037 | 0.535 (0.27) |
|  |  | z = 10.6 (8.7, 12.6) | z = 10.8 (8.9, 12.7) | z = 11.1 (9.3, 13) | z = 11.1 (9.2, 13.1) | z = 10.8 (9, 12.7) |  | z = 15.5 (13.7, 17.3) |
|  |  | d = 0.42 (0.35, 0.5) | d = 0.43 (0.36, 0.5) | d = 0.44 (0.37, 0.51) | d = 0.44 (0.37, 0.52) | d = 0.43 (0.36, 0.5) |  | d = 0.61 (0.54, 0.67) |
|  |  | ES = S | ES = S to M | ES = S to M | ES = S to M | ES = S to M |  | ES = M |
| **Time for caring** |  |  |  |  |  |  |  |  |
| Always enough time | 1,021 | 0.793 (0.24) | 0.745 (0.21) | 0.698 (0.28) | 0.746 (0.22) | 0.697 (0.28) | 1,015 | 0.656 (0.26) |
| Never/sometimes enough time | 1,381 | 0.762 (0.25) | 0.715 (0.21) | 0.656 (0.29) | 0.712 (0.23) | 0.656 (0.30) | 1,368 | 0.601 (0.27) |
|  |  | z = 3.6 (1.7, 5.6) | z = 3.8 (1.8, 5.7) | z = 3.9 (1.9, 5.9) | z = 4 (2, 5.9) | z = 3.6 (1.7, 5.6) |  | z = 5.6 (3.7, 7.6) |
|  |  | d = 0.15 (0.07, 0.23) | d = 0.15 (0.08, 0.23) | d = 0.16 (0.08, 0.24) | d = 0.16 (0.08, 0.24) | d = 0.15 (0.07, 0.23) |  | d = 0.23 (0.15, 0.31) |
|  |  | ES = Nil to S | ES = Nil to S | ES = Nil to S | ES = Nil to S | ES = Nil to S |  | ES = Nil to S |
| **Impact of caring on own health** |  |  |  |  |  |  |  |  |
| No impact | 424 | 0.863 (0.19) | 0.810 (0.17) | 0.793 (0.23) | 0.821 (0.19) | 0.793 (0.24) | 427 | 0.749 (0.24) |
| Impacts | 1,987 | 0.757 (0.25) | 0.710 (0.21) | 0.649 (0.29) | 0.707 (0.23) | 0.648 (0.30) | 1,965 | 0.598 (0.27) |
|  |  | z = 10.8 (8.9, 12.7) | z = 10.9 (9, 12.7) | z = 11.4 (9.4, 13.3) | z = 11.1 (9.2, 12.9) | z = 11 (9.1, 12.8) |  | z = 12.2 (10.3, 14.1) |
|  |  | d = 0.45 (0.37, 0.53) | d = 0.45 (0.37, 0.54) | d = 0.48 (0.39, 0.56) | d = 0.46 (0.38, 0.54) | d = 0.46 (0.38, 0.54) |  | d = 0.52 (0.43, 0.6) |
|  |  | ES = S to M | ES = S to M | ES = S to M | ES = S to M | ES = S to M |  | ES = S to M |
| **Psychological wellbeing** |  |  |  |  |  |  |  |  |
| Good mental wellbeing | 471 | 0.860 (0.19) | 0.808 (0.17) | 0.789 (0.23) | 0.817 (0.19) | 0.789 (0.23) | 463 | 0.780 (0.21) |
| Poor mental wellbeing | 1,940 | 0.755 (0.25) | 0.708 (0.22) | 0.647 (0.29) | 0.705 (0.23) | 0.646 (0.30) | 1,929 | 0.587 (0.27) |
|  |  | z = 10.5 (8.6, 12.3) | z = 10.8 (9, 12.6) | z = 11.5 (9.7, 13.4) | z = 11.3 (9.4, 13.1) | z = 11 (9.2, 12.9) |  | z = 16 (14.2, 17.7) |
|  |  | d = 0.44 (0.36, 0.52) | d = 0.45 (0.37, 0.53) | d = 0.48 (0.4, 0.57) | d = 0.47 (0.39, 0.55) | d = 0.46 (0.38, 0.54) |  | d = 0.69 (0.61, 0.78) |
|  |  | ES = S to M | ES = S to M | ES = S to M | ES = S to M | ES = S to M |  | ES = M |
| **Self-reported quality of life** |  |  |  |  |  |  |  |  |
| Excellent/Very good | 534 | 0.879 (0.18) | 0.827 (0.16) | 0.814 (0.23) | 0.84 (0.18) | 0.817 (0.23) | 527 | 0.784 (0.2) |
| Good | 916 | 0.827 (0.18) | 0.771 (0.17) | 0.731 (0.22) | 0.771 (0.18) | 0.731 (0.23) | 909 | 0.689 (0.21) |
| Poor/Fair | 953 | 0.667 (0.28) | 0.63 (0.23) | 0.541 (0.31) | 0.62 (0.25) | 0.538 (0.32) | 949 | 0.474 (0.27) |
|  |  | KW = 413 (344, 483) | KW = 424 (353, 494) | KW = 452 (382, 522) | KW = 440 (369, 512) | KW = 434 (364, 504) |  | KW = 596 (518, 673) |
|  |  | d = 0.91 (0.82, 1) | d = 0.92 (0.83, 1.02) | d = 0.96 (0.87, 1.05) | d = 0.95 (0.85, 1.04) | d = 0.94 (0.85, 1.03) |  | d = 1.15 (1.05, 1.25) |
|  |  | ES = L | ES = L | ES = L | ES = L | ES = L |  | ES = L |
| All differences between groups were significant (p<0.001) | | | | | | | | |
| z: Mann-Whitney z-score; KW: Kruskal-Wallis H-statistic; d: Cohen’s d effect size | | | | | | | | |
| Cohen's d effect sizes: ≥0.2 small (S), ≥0.5 medium (M) and ≥0.8 large (L) | | | | | | | | |

**Appendix *13*: Known-groups validity - subgroup analysis (EQ-5D-5L country value sets in country datasets)**

|  | **Over 60** | | | | | | | | | |
| --- | --- | --- | --- | --- | --- | --- | --- | --- | --- | --- |
|  | **EQ-5D-5L** | | | | | | | | | |
|  | **Australian value set** | | **Canadian value set** | | **New Zealand value set** | | **United Kingdom value set** | | **United States value set** | |
| **Physical disability** |  |  |  |  |  |  |  |  |  |  |
| No | 217 | 0.947 (0.06) | 217 | 0.877 (0.08) | 163 | 0.914 (0.11) | 207 | 0.907 (0.10) | 149 | 0.905 (0.09) |
| Yes | 203 | 0.801 (0.18) | 213 | 0.699 (0.18) | 184 | 0.695 (0.23) | 120 | 0.688 (0.22) | 158 | 0.588 (0.28) |
|  | z = 12.7 (11.5, 14) | | z = 13.4 (12.3, 14.6) | | z = 11.2 (9.9, 12.5) | | z = 10.8 (9.5, 12.1) | | z = 12.2 (11.2, 13.2) | |
|  | d = 1.59 (1.33, 1.84) | | d = 1.7 (1.44, 1.97) | | d = 1.51 (1.23, 1.78) | | d = 1.49 (1.2, 1.77) | | d = 1.93 (1.62, 2.24) | |
|  | ES = L | | ES = L | | ES = L | | ES = L | | ES = L | |
| **Mental disability** |  |  |  |  |  |  |  |  |  |  |
| No | 345 | 0.905 (0.12) | 339 | 0.825 (0.14) | 282 | 0.825 (0.19) | 269 | 0.874 (0.13) | 241 | 0.814 (0.20) |
| Yes | 70 | 0.750 (0.22) | 82 | 0.649 (0.19) | 53 | 0.665 (0.27) | 50 | 0.587 (0.26) | 59 | 0.475 (0.30) |
|  | z = 8.1 (6.6, 9.6) | | z = 8.9 (7.4, 10.3) | | z = 4.8 (3, 6.5) | | z = 8.5 (7, 10) | | z = 8.4 (7, 9.8) | |
|  | d = 0.87 (0.67, 1.06) | | d = 0.96 (0.76, 1.15) | | d = 0.54 (0.33, 0.76) | | d = 1.08 (0.83, 1.33) | | d = 1.11 (0.87, 1.35) | |
|  | ES = M to L | | ES = M to L | | ES = S to M | | ES = L | | ES = L | |
| **Chronic conditions** |  |  |  |  |  |  |  |  |  |  |
| 0 to 1 health condition | 122 | 0.950 (0.06) | 128 | 0.878 (0.11) | 101 | 0.902 (0.13) | 137 | 0.910 (0.10) | 80 | 0.890 (0.12) |
| 2 or more health conditions | 308 | 0.847 (0.17) | 315 | 0.750 (0.17) | 248 | 0.753 (0.23) | 197 | 0.768 (0.21) | 233 | 0.687 (0.28) |
|  | z = 8.5 (6.9, 10.1) | | z = 9.9 (8.3, 11.4) | | z = 6.9 (5.2, 8.6) | | z = 7.7 (6.1, 9.3) | | z = 6.7 (5.2, 8.3) | |
|  | d = 0.9 (0.7, 1.11) | | d = 1.06 (0.85, 1.28) | | d = 0.79 (0.57, 1.02) | | d = 0.93 (0.7, 1.16) | | d = 0.82 (0.6, 1.05) | |
|  | ES = M to L | | ES = L | | ES = M to L | | ES = M to L | | ES = M to L | |
| **Sleep quality** |  |  |  |  |  |  |  |  |  |  |
| Good to very good | 180 | 0.923 (0.10) | 198 | 0.842 (0.12) | 163 | 0.858 (0.17) | 125 | 0.907 (0.11) | 133 | 0.852 (0.17) |
| Very poor to fair | 250 | 0.842 (0.18) | 244 | 0.742 (0.18) | 185 | 0.741 (0.23) | 209 | 0.779 (0.21) | 177 | 0.657 (0.28) |
|  | z = 6.1 (4.3, 7.9) | | z = 6.9 (5.1, 8.7) | | z = 5.7 (3.8, 7.5) | | z = 7 (5.3, 8.8) | | z = 6.9 (5.2, 8.6) | |
|  | d = 0.56 (0.41, 0.72) | | d = 0.62 (0.48, 0.77) | | d = 0.58 (0.41, 0.75) | | d = 0.72 (0.56, 0.88) | | d = 0.73 (0.57, 0.89) | |
|  | ES = S to M | | ES = S to M | | ES = S to M | | ES = M to L | | ES = M to L | |
| **Social support** |  |  |  |  |  |  |  |  |  |  |
| Good to excellent | 322 | 0.897 (0.13) | 311 | 0.814 (0.15) | 263 | 0.824 (0.20) | 226 | 0.865 (0.15) | 239 | 0.774 (0.23) |
| Poor to fair | 106 | 0.809 (0.19) | 129 | 0.722 (0.18) | 83 | 0.714 (0.23) | 108 | 0.746 (0.23) | 72 | 0.622 (0.32) |
|  | z = 5.5 (3.6, 7.5) | | z = 6.2 (4.3, 8.1) | | z = 4.3 (2.4, 6.2) | | z = 5.6 (3.8, 7.4) | | z = 3.9 (2, 5.8) | |
|  | d = 0.52 (0.35, 0.68) | | d = 0.57 (0.41, 0.73) | | d = 0.45 (0.26, 0.64) | | d = 0.59 (0.42, 0.76) | | d = 0.43 (0.23, 0.63) | |
|  | ES = S to M | | ES = S to M | | ES = S to M | | ES = S to M | | ES = S to M | |
| **Time for caring** |  |  |  |  |  |  |  |  |  |  |
| Always enough time | 304 | 0.885 (0.14) | 263 | 0.801 (0.15) | 212 | 0.819 (0.20) | 215 | 0.843 (0.17) | 198 | 0.765 (0.25) |
| Never/sometimes enough time | 124 | 0.856 (0.19) | 179 | 0.765 (0.18) | 133 | 0.760 (0.23) | 119 | 0.796 (0.22) | 113 | 0.692 (0.27) |
|  | z = 0.6 (1.4, 2.6) | | z = 2.5 (0.6, 4.4) | | z = 2.3 (0.4, 4.3) | | z = 1.6 (0.4, 3.6) | | z = 2.9 (1, 4.8) | |
|  | d = 0.06 (0.13, 0.25) | | d = 0.24 (0.06, 0.41) | | d = 0.25 (0.04, 0.46) | | d = 0.17 (0.05, 0.4) | | d = 0.32 (0.12, 0.53) | |
|  | ES = Nil to S | | ES = Nil to S | | ES = Nil to S | | ES = Nil to S | | ES = Nil to M | |
| **Impact of caring on own health** |  |  |  |  |  |  |  |  |  |  |
| No impact | 197 | 0.908 (0.12) | 174 | 0.833 (0.14) | 161 | 0.855 (0.18) | 133 | 0.883 (0.13) | 114 | 0.819 (0.21) |
| Impacts | 233 | 0.849 (0.17) | 269 | 0.757 (0.17) | 188 | 0.746 (0.23) | 201 | 0.789 (0.21) | 199 | 0.694 (0.28) |
|  | z = 5.4 (3.6, 7.2) | | z = 5.7 (3.8, 7.5) | | z = 5.3 (3.6, 7.1) | | z = 4.9 (3.1, 6.7) | | z = 4.6 (2.7, 6.4) | |
|  | d = 0.54 (0.34, 0.73) | | d = 0.56 (0.37, 0.75) | | d = 0.6 (0.38, 0.81) | | d = 0.56 (0.33, 0.78) | | d = 0.53 (0.3, 0.77) | |
|  | ES = S to M | | ES = S to M | | ES = S to L | | ES = S to M | | ES = S to M | |
| **Psychological wellbeing** |  |  |  |  |  |  |  |  |  |  |
| Good mental wellbeing | 287 | 0.914 (0.11) | 280 | 0.849 (0.10) |  | |  | | 200 | 0.838 (0.18) |
| Poor mental wellbeing | 143 | 0.799 (0.19) | 163 | 0.679 (0.20) |  |  |  |  | 113 | 0.564 (0.29) |
|  | z = 9.1 (7.4, 10.7) | | z = 10.4 (8.9, 12) | |  |  |  |  | z = 9 (7.5, 10.5) | |
|  | d = 0.97 (0.75, 1.19) | | d = 1.14 (0.91, 1.37) | |  |  |  |  | d = 1.19 (0.92, 1.46) | |
|  | ES = M to L | | ES = L | |  |  |  |  | ES = L | |
| **Self-reported quality of life** |  |  |  |  |  |  |  |  |  |  |
| Excellent/Very good | 181 | 0.942 (0.07) | 186 | 0.877 (0.08) | 190 | 0.89 (0.13) | 137 | 0.916 (0.1) | 161 | 0.859 (0.17) |
| Good | 180 | 0.864 (0.15) | 158 | 0.776 (0.13) | 110 | 0.739 (0.2) | 119 | 0.832 (0.14) | 96 | 0.682 (0.25) |
| Poor/Fair | 68 | 0.732 (0.22) | 97 | 0.64 (0.2) | 48 | 0.566 (0.27) | 78 | 0.661 (0.25) | 56 | 0.495 (0.3) |
|  | KW = 116 (83, 148) | | KW = 160 (128, 192) | | KW = 102 (73, 130) | | KW = 98 (71, 125) | | KW = 98 (70, 125) | |
|  | d = 1.21 (0.97, 1.45) | | d = 1.5 (1.26, 1.74) | | d = 1.27 (1.02, 1.53) | | d = 1.28 (1.03, 1.53) | | d = 1.34 (1.06, 1.61) | |
|  | ES = L | | ES = L | | ES = L | | ES = L | | ES = L | |
|  |  |  |  |  |  |  |  |  |  |  |
|  | **Under 60** | | | | | | | | | |
|  | **EQ-5D-5L** | | | | | | | | | |
|  | **Australian value set** | | **Canadian value set** | | **New Zealand value set** | | **United Kingdom value set** | | **United States value set** | |
| **Physical disability** |  |  |  |  |  |  |  |  |  |  |
| No | 644 | 0.898 (0.15) | 513 | 0.832 (0.13) | 727 | 0.865 (0.15) | 664 | 0.873 (0.15) | 563 | 0.842 (0.18) |
| Yes | 306 | 0.702 (0.26) | 237 | 0.612 (0.22) | 302 | 0.585 (0.37) | 282 | 0.600 (0.29) | 321 | 0.513 (0.40) |
|  | z = 14.5 (12.9, 16.1) | | z = 14.3 (12.8, 15.7) | | z = 14.6 (12.9, 16.2) | | z = 15.4 (13.8, 17.1) | | z = 14.4 (12.8, 16.1) | |
|  | d = 1.07 (0.91, 1.22) | | d = 1.22 (1.05, 1.39) | | d = 1.02 (0.87, 1.16) | | d = 1.16 (0.99, 1.32) | | d = 1.11 (0.95, 1.27) | |
|  | ES = L | | ES = L | | ES = L | | ES = L | | ES = L | |
| **Mental disability** |  |  |  |  |  |  |  |  |  |  |
| No | 554 | 0.910 (0.13) | 431 | 0.832 (0.15) | 644 | 0.889 (0.14) | 596 | 0.886 (0.15) | 507 | 0.853 (0.19) |
| Yes | 373 | 0.707 (0.25) | 316 | 0.658 (0.21) | 349 | 0.597 (0.33) | 338 | 0.624 (0.27) | 357 | 0.534 (0.38) |
|  | z = 15.9 (14.4, 17.3) | | z = 13.2 (11.6, 14.8) | | z = 18.5 (17.1, 19.8) | | z = 17.4 (16, 18.8) | | z = 15.8 (14.3, 17.4) | |
|  | d = 1.22 (1.07, 1.38) | | d = 1.11 (0.93, 1.28) | | d = 1.45 (1.28, 1.61) | | d = 1.39 (1.22, 1.55) | | d = 1.28 (1.1, 1.45) | |
|  | ES = L | | ES = L | | ES = L | | ES = L | | ES = L | |
| **Chronic conditions** |  |  |  |  |  |  |  |  |  |  |
| 0 to 1 health condition | 452 | 0.918 (0.13) | 347 | 0.839 (0.14) | 551 | 0.877 (0.19) | 503 | 0.897 (0.15) | 380 | 0.845 (0.26) |
| 2 or more health conditions | 538 | 0.758 (0.24) | 447 | 0.695 (0.21) | 519 | 0.682 (0.30) | 478 | 0.676 (0.25) | 531 | 0.631 (0.33) |
|  | z = 15.3 (13.8, 16.9) | | z = 12.4 (10.7, 14) | | z = 15.4 (13.9, 16.9) | | z = 17.2 (15.7, 18.7) | | z = 14 (12.3, 15.6) | |
|  | d = 1.11 (0.97, 1.26) | | d = 0.98 (0.82, 1.14) | | d = 1.07 (0.93, 1.2) | | d = 1.31 (1.15, 1.48) | | d = 1.04 (0.89, 1.2) | |
|  | ES = L | | ES = L | | ES = L | | ES = L | | ES = L | |
| **Sleep quality** |  |  |  |  |  |  |  |  |  |  |
| Good to very good | 376 | 0.879 (0.20) | 238 | 0.827 (0.16) | 385 | 0.800 (0.33) | 350 | 0.880 (0.18) | 378 | 0.751 (0.38) |
| Very poor to fair | 613 | 0.801 (0.22) | 552 | 0.730 (0.20) | 682 | 0.773 (0.22) | 631 | 0.739 (0.24) | 533 | 0.699 (0.27) |
|  | z = 8.5 (6.6, 10.3) | | z = 8.4 (6.5, 10.2) | | z = 6.7 (4.7, 8.7) | | z = 11.7 (9.9, 13.5) | | z = 7.2 (5.3, 9.2) | |
|  | d = 0.52 (0.41, 0.62) | | d = 0.57 (0.46, 0.68) | | d = 0.4 (0.29, 0.52) | | d = 0.7 (0.6, 0.8) | | d = 0.47 (0.35, 0.59) | |
|  | ES = S to M | | ES = S to M | | ES = S to M | | ES = M | | ES = S to M | |
| **Social support** |  |  |  |  |  |  |  |  |  |  |
| Good to excellent | 699 | 0.854 (0.20) | 537 | 0.789 (0.18) | 747 | 0.805 (0.28) | 664 | 0.829 (0.21) | 686 | 0.748 (0.33) |
| Poor to fair | 288 | 0.773 (0.23) | 249 | 0.695 (0.21) | 301 | 0.740 (0.23) | 311 | 0.706 (0.27) | 218 | 0.639 (0.28) |
|  | z = 7.3 (5.5, 9.1) | | z = 6.5 (4.7, 8.3) | | z = 6.8 (5, 8.7) | | z = 8.1 (6.3, 10) | | z = 7.5 (5.8, 9.2) | |
|  | d = 0.45 (0.35, 0.56) | | d = 0.45 (0.33, 0.57) | | d = 0.41 (0.31, 0.52) | | d = 0.5 (0.4, 0.61) | | d = 0.48 (0.38, 0.59) | |
|  | ES = S to M | | ES = S to M | | ES = S to M | | ES = S to M | | ES = S to M | |
| **Time for caring** |  |  |  |  |  |  |  |  |  |  |
| Always enough time | 448 | 0.846 (0.21) | 310 | 0.768 (0.2) | 448 | 0.772 (0.32) | 412 | 0.827 (0.21) | 419 | 0.701 (0.37) |
| Never/sometimes enough time | 538 | 0.818 (0.21) | 483 | 0.751 (0.19) | 610 | 0.793 (0.22) | 564 | 0.761 (0.24) | 492 | 0.737 (0.27) |
|  | z = 3.7 (1.6, 5.7) | | z = 2.4 (0.4, 4.4) | | z = 1.7 (0.3, 3.7) | | z = 5 (3.1, 6.9) | | z = 0.7 (1.2, 2.6) | |
|  | d = 0.23 (0.11, 0.36) | | d = 0.17 (0.03, 0.31) | | d = 0.1 (0.02, 0.22) | | d = 0.32 (0.2, 0.43) | | d = 0.05 (0.08, 0.17) | |
|  | ES = Nil to S | | ES = Nil to S | | ES = Nil to S | | ES = Nil to S | | ES = Nil | |
| **Impact of caring on own health** |  |  |  |  |  |  |  |  |  |  |
| No impact | 205 | 0.926 (0.13) | 143 | 0.850 (0.15) | 220 | 0.886 (0.14) | 228 | 0.900 (0.16) | 176 | 0.866 (0.18) |
| Impacts | 785 | 0.806 (0.22) | 651 | 0.737 (0.20) | 850 | 0.756 (0.29) | 753 | 0.756 (0.24) | 735 | 0.685 (0.34) |
|  | z = 9.7 (8, 11.4) | | z = 7.9 (6.2, 9.6) | | z = 7.3 (5.5, 9.1) | | z = 10.4 (8.6, 12.1) | | z = 8.3 (6.5, 10) | |
|  | d = 0.65 (0.52, 0.78) | | d = 0.58 (0.44, 0.72) | | d = 0.46 (0.34, 0.58) | | d = 0.7 (0.57, 0.84) | | d = 0.57 (0.44, 0.7) | |
|  | ES = M | | ES = S to M | | ES = S to M | | ES = M to L | | ES = S to M | |
| **Psychological wellbeing** |  |  |  |  |  |  |  |  |  |  |
| Good mental wellbeing | 431 | 0.888 (0.18) | 306 | 0.828 (0.16) |  | |  | | 469 | 0.768 (0.35) |
| Poor mental wellbeing | 559 | 0.787 (0.22) | 488 | 0.714 (0.20) |  |  |  |  | 442 | 0.670 (0.27) |
|  | z = 10.8 (9, 12.6) | | z = 9.7 (8, 11.5) | |  |  |  |  | z = 9.5 (7.7, 11.4) | |
|  | d = 0.73 (0.59, 0.87) | | d = 0.74 (0.58, 0.89) | |  |  |  |  | d = 0.66 (0.52, 0.81) | |
|  | ES = M to L | | ES = M to L | |  |  |  |  | ES = M to L | |
| **Self-reported quality of life** |  |  |  |  |  |  |  |  |  |  |
| Excellent/Very good | 446 | 0.888 (0.18) | 274 | 0.824 (0.15) | 530 | 0.836 (0.27) | 395 | 0.893 (0.15) | 435 | 0.781 (0.34) |
| Good | 329 | 0.847 (0.17) | 305 | 0.79 (0.16) | 330 | 0.783 (0.22) | 294 | 0.817 (0.17) | 283 | 0.728 (0.26) |
| Poor/Fair | 215 | 0.689 (0.26) | 214 | 0.626 (0.22) | 204 | 0.638 (0.28) | 285 | 0.615 (0.29) | 192 | 0.572 (0.3) |
|  | KW = 161 (118, 203) | | KW = 136 (95, 176) | | KW = 163 (121, 205) | | KW = 256 (206, 306) | | KW = 126 (87, 164) | |
|  | d = 0.88 (0.74, 1.02) | | d = 0.9 (0.74, 1.07) | | d = 0.85 (0.72, 0.98) | | d = 1.19 (1.03, 1.35) | | d = 0.79 (0.65, 0.94) | |
|  | ES = M to L | | ES = M to L | | ES = M to L | | ES = L | | ES = M to L | |
|  |  |  |  |  |  |  |  |  |  |  |
|  | **Financially coping** | | | | | | | | | |
|  | **EQ-5D-5L** | | | | | | | | | |
|  | **Australian value set** | | **Canadian value set** | | **New Zealand value set** | | **United Kingdom value set** | | **United States value set** | |
| **Physical disability** |  |  |  |  |  |  |  |  |  |  |
| No | 573 | 0.930 (0.11) | 453 | 0.864 (0.10) | 606 | 0.897 (0.13) | 568 | 0.903 (0.12) | 512 | 0.879 (0.16) |
| Yes | 285 | 0.799 (0.20) | 237 | 0.699 (0.19) | 303 | 0.651 (0.36) | 231 | 0.695 (0.24) | 299 | 0.588 (0.38) |
|  | z = 13.7 (12.1, 15.3) | | z = 14 (12.6, 15.4) | | z = 14.2 (12.5, 15.8) | | z = 13.6 (12, 15.3) | | z = 14.5 (12.9, 16) | |
|  | d = 1.06 (0.9, 1.22) | | d = 1.26 (1.08, 1.44) | | d = 1.06 (0.91, 1.22) | | d = 1.1 (0.93, 1.28) | | d = 1.18 (1.01, 1.34) | |
|  | ES = L | | ES = L | | ES = L | | ES = L | | ES = L | |
| **Mental disability** |  |  |  |  |  |  |  |  |  |  |
| No | 640 | 0.922 (0.11) | 501 | 0.842 (0.13) | 648 | 0.892 (0.15) | 599 | 0.896 (0.13) | 561 | 0.859 (0.18) |
| Yes | 203 | 0.770 (0.22) | 186 | 0.707 (0.19) | 238 | 0.612 (0.38) | 191 | 0.677 (0.24) | 238 | 0.558 (0.40) |
|  | z = 11.9 (10.2, 13.5) | | z = 10.3 (8.6, 11.9) | | z = 14.3 (12.7, 16) | | z = 13.1 (11.5, 14.7) | | z = 13.2 (11.6, 14.8) | |
|  | d = 0.9 (0.74, 1.05) | | d = 0.85 (0.69, 1.01) | | d = 1.1 (0.93, 1.26) | | d = 1.05 (0.89, 1.22) | | d = 1.06 (0.9, 1.22) | |
|  | ES = M to L | | ES = M to L | | ES = L | | ES = L | | ES = L | |
| **Chronic conditions** |  |  |  |  |  |  |  |  |  |  |
| 0 to 1 health condition | 416 | 0.934 (0.11) | 306 | 0.862 (0.13) | 488 | 0.884 (0.20) | 449 | 0.911 (0.12) | 353 | 0.859 (0.25) |
| 2 or more health conditions | 464 | 0.841 (0.18) | 410 | 0.763 (0.17) | 444 | 0.742 (0.29) | 372 | 0.756 (0.22) | 476 | 0.706 (0.31) |
|  | z = 12.2 (10.5, 13.9) | | z = 10.9 (9.1, 12.6) | | z = 11.6 (9.9, 13.3) | | z = 13.2 (11.6, 14.8) | | z = 11.5 (9.8, 13.2) | |
|  | d = 0.9 (0.75, 1.05) | | d = 0.89 (0.71, 1.06) | | d = 0.82 (0.68, 0.96) | | d = 1.04 (0.88, 1.2) | | d = 0.87 (0.71, 1.02) | |
|  | ES = M to L | | ES = M to L | | ES = M to L | | ES = L | | ES = M to L | |
| **Sleep quality** |  |  |  |  |  |  |  |  |  |  |
| Good to very good | 435 | 0.905 (0.16) | 307 | 0.845 (0.14) | 449 | 0.816 (0.31) | 358 | 0.905 (0.14) | 423 | 0.795 (0.34) |
| Very poor to fair | 444 | 0.865 (0.15) | 408 | 0.776 (0.17) | 479 | 0.818 (0.19) | 463 | 0.791 (0.21) | 405 | 0.746 (0.24) |
|  | z = 6.6 (4.7, 8.4) | | z = 7 (5.1, 8.9) | | z = 4.8 (2.9, 6.8) | | z = 10.4 (8.6, 12.3) | | z = 7.3 (5.4, 9.3) | |
|  | d = 0.43 (0.32, 0.55) | | d = 0.51 (0.38, 0.63) | | d = 0.31 (0.19, 0.44) | | d = 0.69 (0.58, 0.79) | | d = 0.49 (0.37, 0.62) | |
|  | ES = S to M | | ES = S to M | | ES = Nil to S | | ES = M | | ES = S to M | |
| **Social support** |  |  |  |  |  |  |  |  |  |  |
| Good to excellent | 715 | 0.891 (0.16) | 556 | 0.818 (0.16) | 734 | 0.822 (0.27) | 620 | 0.866 (0.17) | 698 | 0.781 (0.30) |
| Poor to fair | 160 | 0.858 (0.15) | 156 | 0.757 (0.16) | 180 | 0.804 (0.19) | 198 | 0.765 (0.23) | 122 | 0.726 (0.23) |
|  | z = 4.3 (2.5, 6.2) | | z = 5.4 (3.6, 7.2) | | z = 3.8 (2, 5.7) | | z = 6.5 (4.6, 8.4) | | z = 4.6 (2.8, 6.4) | |
|  | d = 0.29 (0.17, 0.41) | | d = 0.4 (0.27, 0.52) | | d = 0.25 (0.13, 0.37) | | d = 0.44 (0.32, 0.57) | | d = 0.32 (0.19, 0.44) | |
|  | ES = Nil to S | | ES = S to M | | ES = Nil to S | | ES = S to M | | ES = Nil to S | |
| **Time for caring** |  |  |  |  |  |  |  |  |  |  |
| Always enough time | 502 | 0.891 (0.16) | 364 | 0.817 (0.16) | 451 | 0.806 (0.31) | 435 | 0.863 (0.17) | 441 | 0.755 (0.34) |
| Never/sometimes enough time | 375 | 0.878 (0.16) | 350 | 0.793 (0.16) | 468 | 0.831 (0.19) | 384 | 0.815 (0.21) | 387 | 0.789 (0.24) |
|  | z = 2 (0.2, 3.9) | | z = 2.6 (0.6, 4.6) | | z = 1.8 (0.2, 3.8) | | z = 3.6 (1.7, 5.6) | | z = 0.8 (1.1, 2.8) | |
|  | d = 0.14 (0.01, 0.26) | | d = 0.19 (0.05, 0.34) | | d = 0.12 (0.01, 0.25) | | d = 0.25 (0.12, 0.39) | | d = 0.06 (0.08, 0.2) | |
|  | ES = Nil to S | | ES = Nil to S | | ES = Nil to S | | ES = Nil to S | | ES = Nil | |
| **Impact of caring on own health** |  |  |  |  |  |  |  |  |  |  |
| No impact | 307 | 0.937 (0.09) | 228 | 0.858 (0.12) | 291 | 0.886 (0.16) | 271 | 0.911 (0.12) | 224 | 0.874 (0.16) |
| Impacts | 573 | 0.857 (0.18) | 488 | 0.781 (0.17) | 641 | 0.785 (0.29) | 550 | 0.806 (0.21) | 605 | 0.733 (0.32) |
|  | z = 8.6 (6.7, 10.4) | | z = 6.5 (4.8, 8.3) | | z = 6.2 (4.4, 7.9) | | z = 8.4 (6.7, 10.2) | | z = 7 (5.1, 8.8) | |
|  | d = 0.6 (0.46, 0.75) | | d = 0.5 (0.36, 0.65) | | d = 0.41 (0.29, 0.53) | | d = 0.62 (0.48, 0.76) | | d = 0.5 (0.36, 0.64) | |
|  | ES = S to M | | ES = S to M | | ES = S to M | | ES = S to M | | ES = S to M | |
| **Psychological wellbeing** |  |  |  |  |  |  |  |  |  |  |
| Good mental wellbeing | 536 | 0.905 (0.16) | 413 | 0.849 (0.13) |  | |  | | 546 | 0.797 (0.32) |
| Poor mental wellbeing | 344 | 0.854 (0.15) | 303 | 0.746 (0.18) |  |  |  |  | 283 | 0.720 (0.23) |
|  | z = 8.1 (6.2, 9.9) | | z = 9 (7.2, 10.8) | |  |  |  |  | z = 8 (6.2, 9.8) | |
|  | d = 0.56 (0.43, 0.7) | | d = 0.72 (0.55, 0.88) | |  |  |  |  | d = 0.58 (0.43, 0.72) | |
|  | ES = S to M | | ES = M to L | |  |  |  |  | ES = S to M | |
| **Self-reported quality of life** |  |  |  |  |  |  |  |  |  |  |
| Excellent/Very good | 502 | 0.908 (0.15) | 362 | 0.856 (0.13) | 593 | 0.848 (0.26) | 426 | 0.91 (0.12) | 507 | 0.807 (0.32) |
| Good | 294 | 0.885 (0.13) | 248 | 0.791 (0.15) | 249 | 0.786 (0.21) | 254 | 0.832 (0.16) | 238 | 0.743 (0.24) |
| Poor/Fair | 84 | 0.747 (0.19) | 104 | 0.672 (0.19) | 85 | 0.679 (0.32) | 138 | 0.645 (0.26) | 83 | 0.632 (0.26) |
|  | KW = 104 (68, 140) | | KW = 135 (96, 174) | | KW = 82 (49, 115) | | KW = 183 (142, 225) | | KW = 87 (54, 121) | |
|  | d = 0.73 (0.58, 0.87) | | d = 0.96 (0.79, 1.13) | | d = 0.62 (0.48, 0.76) | | d = 1.07 (0.91, 1.23) | | d = 0.68 (0.53, 0.83) | |
|  | ES = M to L | | ES = M to L | | ES = S to M | | ES = L | | ES = M to L | |
|  |  |  |  |  |  |  |  |  |  |  |
|  | **Financially strained** | | | | | | | | | |
|  | **EQ-5D-5L** | | | | | | | | | |
|  | **Australian value set** | | **Canadian value set** | | **New Zealand value set** | | **United Kingdom value set** | | **United States value set** | |
| **Physical disability** |  |  |  |  |  |  |  |  |  |  |
| No | 284 | 0.870 (0.16) | 276 | 0.814 (0.14) | 276 | 0.828 (0.17) | 301 | 0.840 (0.16) | 200 | 0.794 (0.18) |
| Yes | 222 | 0.667 (0.26) | 212 | 0.603 (0.22) | 181 | 0.586 (0.26) | 168 | 0.530 (0.29) | 179 | 0.452 (0.33) |
|  | z = 11.9 (10.4, 13.4) | | z = 12.3 (10.8, 13.7) | | z = 11 (9.6, 12.5) | | z = 12.6 (11.2, 13.9) | | z = 11.2 (9.9, 12.6) | |
|  | d = 1.24 (1.03, 1.46) | | d = 1.33 (1.1, 1.57) | | d = 1.21 (0.99, 1.42) | | d = 1.42 (1.19, 1.65) | | d = 1.41 (1.16, 1.66) | |
|  | ES = L | | ES = L | | ES = L | | ES = L | | ES = L | |
| **Mental disability** |  |  |  |  |  |  |  |  |  |  |
| No | 255 | 0.873 (0.15) | 267 | 0.806 (0.15) | 275 | 0.817 (0.19) | 261 | 0.851 (0.17) | 187 | 0.783 (0.21) |
| Yes | 238 | 0.664 (0.26) | 212 | 0.611 (0.21) | 159 | 0.597 (0.24) | 196 | 0.561 (0.27) | 177 | 0.482 (0.32) |
|  | z = 11.2 (9.7, 12.8) | | z = 11.2 (9.7, 12.6) | | z = 10.7 (9.2, 12.1) | | z = 12.9 (11.7, 14.2) | | z = 10.2 (8.7, 11.7) | |
|  | d = 1.17 (0.96, 1.39) | | d = 1.19 (0.98, 1.4) | | d = 1.19 (0.97, 1.41) | | d = 1.52 (1.29, 1.76) | | d = 1.27 (1, 1.53) | |
|  | ES = L | | ES = L | | ES = L | | ES = L | | ES = L | |
| **Chronic conditions** |  |  |  |  |  |  |  |  |  |  |
| 0 to 1 health condition | 154 | 0.897 (0.14) | 168 | 0.827 (0.14) | 158 | 0.882 (0.12) | 189 | 0.873 (0.17) | 107 | 0.834 (0.20) |
| 2 or more health conditions | 380 | 0.728 (0.25) | 351 | 0.665 (0.21) | 319 | 0.652 (0.25) | 298 | 0.635 (0.26) | 287 | 0.552 (0.31) |
|  | z = 10.2 (8.5, 11.8) | | z = 10.3 (8.7, 12) | | z = 11.6 (10.2, 13) | | z = 12.1 (10.7, 13.5) | | z = 9.6 (8.2, 11.1) | |
|  | d = 0.98 (0.79, 1.18) | | d = 1.02 (0.82, 1.22) | | d = 1.26 (1.05, 1.47) | | d = 1.31 (1.09, 1.52) | | d = 1.11 (0.89, 1.33) | |
|  | ES = M to L | | ES = L | | ES = L | | ES = L | | ES = L | |
| **Sleep quality** |  |  |  |  |  |  |  |  |  |  |
| Good to very good | 117 | 0.843 (0.21) | 128 | 0.807 (0.16) | 96 | 0.827 (0.19) | 115 | 0.834 (0.22) | 88 | 0.690 (0.33) |
| Very poor to fair | 417 | 0.758 (0.24) | 387 | 0.690 (0.20) | 381 | 0.704 (0.25) | 372 | 0.695 (0.26) | 304 | 0.612 (0.30) |
|  | z = 4.7 (2.9, 6.6) | | z = 6.6 (4.7, 8.5) | | z = 5.2 (3.5, 7) | | z = 6.8 (4.9, 8.6) | | z = 3 (1, 5.1) | |
|  | d = 0.4 (0.25, 0.56) | | d = 0.56 (0.41, 0.7) | | d = 0.47 (0.32, 0.62) | | d = 0.59 (0.44, 0.73) | | d = 0.3 (0.11, 0.5) | |
|  | ES = S to M | | ES = S to M | | ES = S to M | | ES = S to M | | ES = Nil to M | |
| **Social support** |  |  |  |  |  |  |  |  |  |  |
| Good to excellent | 302 | 0.812 (0.22) | 290 | 0.761 (0.18) | 269 | 0.778 (0.22) | 267 | 0.773 (0.23) | 226 | 0.674 (0.30) |
| Poor to fair | 232 | 0.731 (0.25) | 222 | 0.666 (0.22) | 204 | 0.673 (0.25) | 217 | 0.671 (0.28) | 168 | 0.568 (0.31) |
|  | z = 5 (3.2, 6.9) | | z = 5.1 (3.3, 7) | | z = 5.3 (3.4, 7.2) | | z = 4.9 (3.1, 6.7) | | z = 4 (2.1, 5.9) | |
|  | d = 0.43 (0.28, 0.58) | | d = 0.44 (0.29, 0.59) | | d = 0.47 (0.31, 0.63) | | d = 0.44 (0.28, 0.59) | | d = 0.39 (0.21, 0.57) | |
|  | ES = S to M | | ES = S to M | | ES = S to M | | ES = S to M | | ES = S to M | |
| **Time for caring** |  |  |  |  |  |  |  |  |  |  |
| Always enough time | 246 | 0.798 (0.22) | 207 | 0.727 (0.20) | 205 | 0.748 (0.23) | 188 | 0.761 (0.25) | 175 | 0.639 (0.33) |
| Never/sometimes enough time | 285 | 0.756 (0.25) | 312 | 0.711 (0.20) | 270 | 0.712 (0.25) | 296 | 0.706 (0.26) | 218 | 0.620 (0.29) |
|  | z = 2.1 (0.2, 4) | | z = 1.1 (0.8, 3.1) | | z = 1.3 (0.7, 3.2) | | z = 2.5 (0.5, 4.4) | | z = 1.3 (0.8, 3.3) | |
|  | d = 0.18 (0.02, 0.35) | | d = 0.1 (0.07, 0.27) | | d = 0.12 (0.06, 0.29) | | d = 0.22 (0.05, 0.39) | | d = 0.13 (0.07, 0.33) | |
|  | ES = Nil to S | | ES = Nil to S | | ES = Nil to S | | ES = Nil to S | | ES = Nil to S | |
| **Impact of caring on own health** |  |  |  |  |  |  |  |  |  |  |
| No impact | 92 | 0.846 (0.18) | 88 | 0.800 (0.18) | 88 | 0.832 (0.15) | 90 | 0.842 (0.22) | 66 | 0.758 (0.26) |
| Impacts | 442 | 0.762 (0.24) | 431 | 0.700 (0.20) | 389 | 0.705 (0.25) | 397 | 0.702 (0.26) | 328 | 0.603 (0.31) |
|  | z = 3.9 (2, 5.9) | | z = 5.4 (3.5, 7.3) | | z = 4.5 (2.8, 6.2) | | z = 6 (4.1, 7.8) | | z = 4.5 (2.5, 6.4) | |
|  | d = 0.35 (0.17, 0.52) | | d = 0.49 (0.3, 0.67) | | d = 0.42 (0.25, 0.59) | | d = 0.56 (0.37, 0.75) | | d = 0.46 (0.25, 0.67) | |
|  | ES = Nil to M | | ES = S to M | | ES = S to M | | ES = S to M | | ES = S to M | |
| **Psychological wellbeing** |  |  |  |  |  |  |  |  |  |  |
| Good mental wellbeing | 177 | 0.877 (0.16) | 172 | 0.811 (0.15) |  | |  | | 122 | 0.753 (0.28) |
| Poor mental wellbeing | 357 | 0.726 (0.25) | 347 | 0.671 (0.21) |  |  |  |  | 272 | 0.573 (0.30) |
|  | z = 8.9 (7.2, 10.6) | | z = 8.3 (6.6, 10.1) | |  |  |  |  | z = 6.8 (5, 8.5) | |
|  | d = 0.83 (0.64, 1.03) | | d = 0.79 (0.6, 0.98) | |  |  |  |  | d = 0.72 (0.51, 0.93) | |
|  | ES = M to L | | ES = M to L | |  |  |  |  | ES = M to L | |
| **Self-reported quality of life** |  |  |  |  |  |  |  |  |  |  |
| Excellent/Very good | 121 | 0.880 (0.18) | 97 | 0.808 (0.15) | 122 | 0.867 (0.16) | 105 | 0.854 (0.20) | 89 | 0.772 (0.26) |
| Good | 214 | 0.809 (0.19) | 215 | 0.779 (0.15) | 189 | 0.755 (0.22) | 157 | 0.804 (0.15) | 141 | 0.671 (0.28) |
| Poor/Fair | 198 | 0.678 (0.27) | 206 | 0.611 (0.22) | 164 | 0.595 (0.25) | 221 | 0.609 (0.29) | 164 | 0.514 (0.31) |
|  | KW = 82 (50, 114) | | KW = 91 (58, 124) | | KW = 121 (90, 151) | | KW = 99 (65, 133) | | KW = 60 (33, 86) | |
|  | d = 0.84 (0.64, 1.04) | | d = 0.91 (0.71, 1.12) | | d = 1.16 (0.96, 1.36) | | d = 1.01 (0.78, 1.23) | | d = 0.83 (0.61, 1.06) | |
|  | ES = M to L | | ES = M to L | | ES = L | | ES = M to L | | ES = M to L | |
| All differences between groups were significant (p<0.001) | | | | | | | | | | |
| z: Mann-Whitney z-score; KW: Kruskal-Wallis H-statistic; d: Cohen’s d effect size | | | | | | | | | | |
| Cohen's d effect sizes: ≥0.2 small (S), ≥0.5 medium (M) and ≥0.8 large (L) | | | | | | | | | | |

**Appendix *14*: Known-groups validity - subgroup analysis (EQ-HWB-9 UK pilot value set in country datasets)**

|  | **Over 60** | | | | | | | | | |
| --- | --- | --- | --- | --- | --- | --- | --- | --- | --- | --- |
|  | **EQ-HWB-9 UK pilot value set** | | | | | | | | | |
|  | **Australian dataset** | | **Canadian dataset** | | **New Zealand dataset** | | **United Kingdom dataset** | | **United States dataset** | |
| **Physical disability** |  |  |  |  |  |  |  |  |  |  |
| No | 219 | 0.878 (0.13) | 216 | 0.874 (0.13) | 155 | 0.892 (0.11) | 207 | 0.882 (0.13) | 152 | 0.881 (0.12) |
| Yes | 205 | 0.718 (0.21) | 209 | 0.684 (0.21) | 180 | 0.745 (0.19) | 120 | 0.673 (0.26) | 158 | 0.619 (0.25) |
|  | z = 10.2 (8.7, 11.8) | | z = 11.3 (9.8, 12.7) | | z = 9.4 (7.8, 11) | | z = 9.5 (8.1, 10.9) | | z = 10.9 (9.7, 12.2) | |
|  | d = 1.14 (0.91, 1.37) | | d = 1.3 (1.06, 1.54) | | d = 1.2 (0.93, 1.47) | | d = 1.23 (0.98, 1.49) | | d = 1.58 (1.29, 1.87) | |
|  | ES = L | | ES = L | | ES = L | | ES = L | | ES = L | |
| **Mental disability** |  |  |  |  |  |  |  |  |  |  |
| No | 348 | 0.845 (0.15) | 336 | 0.832 (0.16) | 272 | 0.839 (0.15) | 269 | 0.864 (0.13) | 242 | 0.817 (0.18) |
| Yes | 71 | 0.588 (0.23) | 80 | 0.593 (0.23) | 51 | 0.684 (0.22) | 50 | 0.516 (0.30) | 62 | 0.502 (0.27) |
|  | z = 9.4 (8, 10.8) | | z = 9.4 (8, 10.8) | | z = 5.3 (3.6, 7.1) | | z = 8.4 (6.9, 10) | | z = 8.5 (7, 9.9) | |
|  | d = 1.04 (0.84, 1.23) | | d = 1.04 (0.84, 1.24) | | d = 0.62 (0.4, 0.85) | | d = 1.07 (0.81, 1.33) | | d = 1.11 (0.86, 1.36) | |
|  | ES = L | | ES = L | | ES = S to L | | ES = L | | ES = L | |
| **Chronic conditions** |  |  |  |  |  |  |  |  |  |  |
| 0 to 1 health condition | 125 | 0.891 (0.12) | 126 | 0.885 (0.11) | 98 | 0.885 (0.13) | 138 | 0.892 (0.11) | 80 | 0.881 (0.11) |
| 2 or more health conditions | 309 | 0.759 (0.21) | 312 | 0.734 (0.21) | 239 | 0.781 (0.18) | 197 | 0.742 (0.24) | 237 | 0.700 (0.25) |
|  | z = 7.9 (6.2, 9.6) | | z = 8.1 (6.4, 9.7) | | z = 6.4 (4.5, 8.3) | | z = 7.2 (5.6, 8.8) | | z = 6.4 (4.8, 7.9) | |
|  | d = 0.82 (0.61, 1.02) | | d = 0.84 (0.63, 1.04) | | d = 0.74 (0.49, 0.99) | | d = 0.85 (0.62, 1.08) | | d = 0.77 (0.55, 0.98) | |
|  | ES = M to L | | ES = M to L | | ES = S to L | | ES = M to L | | ES = M to L | |
| **Sleep quality** |  |  |  |  |  |  |  |  |  |  |
| Good to very good | 182 | 0.869 (0.15) | 195 | 0.852 (0.14) | 159 | 0.869 (0.12) | 123 | 0.892 (0.13) | 134 | 0.862 (0.15) |
| Very poor to fair | 252 | 0.745 (0.21) | 242 | 0.717 (0.22) | 178 | 0.759 (0.20) | 212 | 0.753 (0.23) | 181 | 0.659 (0.26) |
|  | z = 7.9 (6.2, 9.6) | | z = 7.6 (5.8, 9.3) | | z = 6.2 (4.5, 7.9) | | z = 7.4 (5.6, 9.1) | | z = 8.4 (6.8, 10) | |
|  | d = 0.71 (0.57, 0.84) | | d = 0.68 (0.54, 0.82) | | d = 0.64 (0.48, 0.8) | | d = 0.75 (0.59, 0.9) | | d = 0.86 (0.72, 0.99) | |
|  | ES = M to L | | ES = M to L | | ES = S to M | | ES = M to L | | ES = M to L | |
| **Social support** |  |  |  |  |  |  |  |  |  |  |
| Good to excellent | 324 | 0.834 (0.16) | 306 | 0.824 (0.17) | 256 | 0.841 (0.16) | 225 | 0.859 (0.15) | 241 | 0.787 (0.21) |
| Poor to fair | 108 | 0.684 (0.25) | 129 | 0.670 (0.22) | 78 | 0.715 (0.19) | 110 | 0.690 (0.26) | 74 | 0.607 (0.28) |
|  | z = 6.1 (4.3, 7.9) | | z = 7.9 (6.2, 9.6) | | z = 6 (4.4, 7.7) | | z = 7.2 (5.5, 8.9) | | z = 5.4 (3.6, 7.2) | |
|  | d = 0.56 (0.41, 0.71) | | d = 0.71 (0.58, 0.84) | | d = 0.63 (0.47, 0.78) | | d = 0.73 (0.58, 0.89) | | d = 0.58 (0.41, 0.76) | |
|  | ES = S to M | | ES = M to L | | ES = S to M | | ES = M to L | | ES = S to M | |
| **Time for caring** |  |  |  |  |  |  |  |  |  |  |
| Always enough time | 309 | 0.813 (0.18) | 261 | 0.803 (0.18) | 208 | 0.836 (0.15) | 215 | 0.830 (0.17) | 197 | 0.779 (0.23) |
| Never/sometimes enough time | 123 | 0.760 (0.23) | 176 | 0.739 (0.23) | 125 | 0.767 (0.20) | 120 | 0.758 (0.26) | 118 | 0.688 (0.25) |
|  | z = 2.3 (0.4, 4.2) | | z = 3.1 (1.2, 5) | | z = 3.1 (1.1, 5) | | z = 2.1 (0.1, 4.1) | | z = 3.8 (1.9, 5.6) | |
|  | d = 0.22 (0.04, 0.4) | | d = 0.3 (0.12, 0.47) | | d = 0.33 (0.13, 0.54) | | d = 0.23 (0.02, 0.44) | | d = 0.42 (0.22, 0.61) | |
|  | ES = Nil to S | | ES = Nil to S | | ES = Nil to M | | ES = Nil to S | | ES = S to M | |
| **Impact of caring on own health** |  |  |  |  |  |  |  |  |  |  |
| No impact | 199 | 0.869 (0.14) | 175 | 0.849 (0.15) | 158 | 0.876 (0.12) | 136 | 0.871 (0.14) | 120 | 0.825 (0.21) |
| Impacts | 235 | 0.736 (0.22) | 263 | 0.730 (0.22) | 179 | 0.754 (0.19) | 199 | 0.758 (0.24) | 197 | 0.697 (0.24) |
|  | z = 8.2 (6.5, 9.9) | | z = 6.8 (5, 8.5) | | z = 7.5 (5.9, 9.2) | | z = 5.5 (3.8, 7.2) | | z = 6.2 (4.3, 8) | |
|  | d = 0.86 (0.65, 1.07) | | d = 0.68 (0.49, 0.88) | | d = 0.9 (0.66, 1.13) | | d = 0.63 (0.41, 0.85) | | d = 0.74 (0.49, 0.99) | |
|  | ES = M to L | | ES = S to L | | ES = M to L | | ES = S to L | | ES = S to L | |
| **Psychological wellbeing** |  |  |  |  |  |  |  |  |  |  |
| Good mental wellbeing | 289 | 0.871 (0.12) | 280 | 0.869 (0.11) |  | |  | | 201 | 0.855 (0.15) |
| Poor mental wellbeing | 145 | 0.650 (0.23) | 158 | 0.615 (0.23) |  |  |  |  | 116 | 0.556 (0.24) |
|  | z = 11.2 (9.9, 12.6) | | z = 12.6 (11.2, 13.9) | |  |  |  |  | z = 11.4 (10.2, 12.5) | |
|  | d = 1.28 (1.06, 1.51) | | d = 1.5 (1.25, 1.75) | |  |  |  |  | d = 1.66 (1.37, 1.95) | |
|  | ES = L | | ES = L | |  |  |  |  | ES = L | |
| **Self-reported quality of life** |  |  |  |  |  |  |  |  |  |  |
| Excellent/Very good | 183 | 0.889 (0.13) | 185 | 0.897 (0.09) | 184 | 0.889 (0.10) | 140 | 0.909 (0.10) | 162 | 0.868 (0.15) |
| Good | 183 | 0.793 (0.15) | 157 | 0.781 (0.15) | 105 | 0.771 (0.15) | 117 | 0.819 (0.14) | 97 | 0.706 (0.20) |
| Poor/Fair | 67 | 0.561 (0.24) | 95 | 0.540 (0.23) | 47 | 0.603 (0.24) | 78 | 0.593 (0.28) | 58 | 0.470 (0.24) |
|  | KW = 139 (107, 171) | | KW = 202 (171, 233) | | KW = 109 (79, 140) | | KW = 119 (90, 147) | | KW = 134 (108, 161) | |
|  | d = 1.37 (1.13, 1.6) | | d = 1.85 (1.59, 2.11) | | d = 1.38 (1.09, 1.67) | | d = 1.47 (1.19, 1.75) | | d = 1.71 (1.41, 2) | |
|  | ES = L | | ES = L | | ES = L | | ES = L | | ES = L | |
|  |  |  |  |  |  |  |  |  |  |  |
|  | **Under 60** | | | | | | | | | |
|  | **EQ-5D-5L** | | | | | | | | | |
|  | **Australian value set** | | **Canadian value set** | | **New Zealand value set** | | **United Kingdom value set** | | **United States value set** | |
| **Physical disability** |  |  |  |  |  |  |  |  |  |  |
| No | 644 | 0.739 (0.23) | 504 | 0.732 (0.20) | 706 | 0.789 (0.18) | 661 | 0.777 (0.19) | 563 | 0.755 (0.20) |
| Yes | 309 | 0.507 (0.27) | 237 | 0.503 (0.26) | 299 | 0.530 (0.27) | 278 | 0.506 (0.31) | 323 | 0.474 (0.31) |
|  | z = 12.8 (11.2, 14.4) | | z = 11.6 (10, 13.2) | | z = 14.9 (13.3, 16.4) | | z = 13.8 (12.2, 15.4) | | z = 13.9 (12.4, 15.4) | |
|  | d = 0.91 (0.78, 1.05) | | d = 0.94 (0.78, 1.1) | | d = 1.06 (0.92, 1.2) | | d = 1.01 (0.86, 1.15) | | d = 1.05 (0.91, 1.2) | |
|  | ES = M to L | | ES = M to L | | ES = L | | ES = L | | ES = L | |
| **Mental disability** |  |  |  |  |  |  |  |  |  |  |
| No | 554 | 0.762 (0.22) | 423 | 0.757 (0.19) | 626 | 0.820 (0.16) | 591 | 0.804 (0.18) | 511 | 0.787 (0.18) |
| Yes | 373 | 0.501 (0.27) | 315 | 0.518 (0.25) | 345 | 0.526 (0.25) | 334 | 0.504 (0.29) | 358 | 0.460 (0.30) |
|  | z = 15 (13.5, 16.5) | | z = 13.2 (11.7, 14.7) | | z = 18.5 (17.3, 19.8) | | z = 16.8 (15.4, 18.3) | | z = 16.9 (15.6, 18.2) | |
|  | d = 1.13 (0.98, 1.28) | | d = 1.11 (0.94, 1.28) | | d = 1.48 (1.33, 1.63) | | d = 1.33 (1.17, 1.49) | | d = 1.4 (1.24, 1.56) | |
|  | ES = L | | ES = L | | ES = L | | ES = L | | ES = L | |
| **Chronic conditions** |  |  |  |  |  |  |  |  |  |  |
| 0 to 1 health condition | 454 | 0.758 (0.23) | 341 | 0.754 (0.19) | 532 | 0.797 (0.19) | 504 | 0.801 (0.19) | 379 | 0.767 (0.23) |
| 2 or more health conditions | 537 | 0.576 (0.27) | 444 | 0.577 (0.25) | 515 | 0.623 (0.25) | 469 | 0.578 (0.28) | 534 | 0.566 (0.29) |
|  | z = 11.6 (9.9, 13.3) | | z = 10.2 (8.5, 11.9) | | z = 13 (11.2, 14.7) | | z = 14.2 (12.6, 15.8) | | z = 12 (10.2, 13.7) | |
|  | d = 0.79 (0.66, 0.93) | | d = 0.78 (0.63, 0.93) | | d = 0.87 (0.73, 1.01) | | d = 1.02 (0.88, 1.17) | | d = 0.86 (0.71, 1.01) | |
|  | ES = M to L | | ES = M to L | | ES = M to L | | ES = L | | ES = M to L | |
| **Sleep quality** |  |  |  |  |  |  |  |  |  |  |
| Good to very good | 378 | 0.705 (0.27) | 233 | 0.764 (0.20) | 377 | 0.739 (0.28) | 351 | 0.793 (0.24) | 378 | 0.690 (0.32) |
| Very poor to fair | 612 | 0.631 (0.26) | 549 | 0.610 (0.25) | 668 | 0.697 (0.21) | 622 | 0.637 (0.26) | 535 | 0.621 (0.25) |
|  | z = 5.5 (3.6, 7.4) | | z = 8.8 (6.9, 10.6) | | z = 6.4 (4.3, 8.5) | | z = 11.1 (9.3, 12.9) | | z = 6.3 (4.3, 8.2) | |
|  | d = 0.34 (0.23, 0.46) | | d = 0.6 (0.48, 0.71) | | d = 0.39 (0.26, 0.51) | | d = 0.67 (0.58, 0.77) | | d = 0.41 (0.29, 0.53) | |
|  | ES = S | | ES = S to M | | ES = S to M | | ES = M | | ES = S to M | |
| **Social support** |  |  |  |  |  |  |  |  |  |  |
| Good to excellent | 696 | 0.700 (0.25) | 528 | 0.712 (0.21) | 736 | 0.742 (0.24) | 665 | 0.736 (0.24) | 687 | 0.687 (0.28) |
| Poor to fair | 292 | 0.563 (0.28) | 249 | 0.539 (0.26) | 290 | 0.642 (0.23) | 303 | 0.599 (0.29) | 220 | 0.534 (0.26) |
|  | z = 7.5 (5.8, 9.3) | | z = 8.7 (7, 10.5) | | z = 7.7 (6.1, 9.4) | | z = 7.9 (6.1, 9.7) | | z = 8.3 (6.6, 10.1) | |
|  | d = 0.46 (0.36, 0.57) | | d = 0.6 (0.49, 0.71) | | d = 0.47 (0.37, 0.57) | | d = 0.49 (0.39, 0.6) | | d = 0.53 (0.43, 0.64) | |
|  | ES = S to M | | ES = S to M | | ES = S to M | | ES = S to M | | ES = S to M | |
| **Time for caring** |  |  |  |  |  |  |  |  |  |  |
| Always enough time | 448 | 0.671 (0.28) | 311 | 0.669 (0.25) | 439 | 0.698 (0.27) | 410 | 0.733 (0.27) | 423 | 0.633 (0.31) |
| Never/sometimes enough time | 539 | 0.650 (0.26) | 473 | 0.645 (0.24) | 597 | 0.724 (0.22) | 558 | 0.663 (0.26) | 490 | 0.664 (0.25) |
|  | z = 2.1 (0.1, 4) | | z = 1.7 (0.3, 3.6) | | z = 0.2 (2.2, 1.7) | | z = 5.6 (3.7, 7.5) | | z = 0.4 (2.4, 1.5) | |
|  | d = 0.13 (0.01, 0.25) | | d = 0.12 (0.02, 0.26) | | d = 0.01 (0.14, 0.11) | | d = 0.35 (0.24, 0.47) | | d = 0.03 (0.16, 0.1) | |
|  | ES = Nil to S | | ES = Nil to S | | ES = Nil | | ES = S | | ES = Nil | |
| **Impact of caring on own health** |  |  |  |  |  |  |  |  |  |  |
| No impact | 206 | 0.854 (0.18) | 144 | 0.797 (0.20) | 222 | 0.835 (0.18) | 228 | 0.836 (0.20) | 179 | 0.819 (0.22) |
| Impacts | 785 | 0.609 (0.26) | 641 | 0.622 (0.24) | 825 | 0.678 (0.25) | 745 | 0.650 (0.26) | 734 | 0.608 (0.28) |
|  | z = 13.8 (12.2, 15.5) | | z = 8.8 (6.9, 10.7) | | z = 10.2 (8.4, 12) | | z = 11.6 (9.9, 13.4) | | z = 10.7 (9, 12.5) | |
|  | d = 0.98 (0.83, 1.13) | | d = 0.66 (0.5, 0.82) | | d = 0.66 (0.53, 0.79) | | d = 0.8 (0.67, 0.94) | | d = 0.76 (0.62, 0.9) | |
|  | ES = L | | ES = M to L | | ES = M | | ES = M to L | | ES = M to L | |
| **Psychological wellbeing** |  |  |  |  |  |  |  |  |  |  |
| Good mental wellbeing | 431 | 0.753 (0.26) | 299 | 0.785 (0.19) |  | |  | | 471 | 0.719 (0.30) |
| Poor mental wellbeing | 560 | 0.588 (0.25) | 486 | 0.574 (0.24) |  |  |  |  | 442 | 0.575 (0.24) |
|  | z = 12 (10.2, 13.8) | | z = 13 (11.4, 14.6) | |  |  |  |  | z = 11.1 (9.2, 12.9) | |
|  | d = 0.82 (0.68, 0.97) | | d = 1.05 (0.88, 1.21) | |  |  |  |  | d = 0.79 (0.64, 0.94) | |
|  | ES = M to L | | ES = L | |  |  |  |  | ES = M to L | |
| **Self-reported quality of life** |  |  |  |  |  |  |  |  |  |  |
| Excellent/Very good | 447 | 0.745 (0.24) | 270 | 0.756 (0.20) | 516 | 0.779 (0.23) | 396 | 0.801 (0.22) | 433 | 0.719 (0.30) |
| Good | 327 | 0.693 (0.21) | 301 | 0.697 (0.19) | 322 | 0.716 (0.19) | 294 | 0.724 (0.19) | 291 | 0.645 (0.23) |
| Poor/Fair | 217 | 0.433 (0.27) | 213 | 0.466 (0.26) | 204 | 0.533 (0.24) | 278 | 0.504 (0.29) | 189 | 0.496 (0.26) |
|  | KW = 202 (157, 247) | | KW = 168 (127, 210) | | KW = 193 (146, 240) | | KW = 236 (189, 284) | | KW = 126 (87, 166) | |
|  | d = 1.01 (0.87, 1.15) | | d = 1.04 (0.88, 1.2) | | d = 0.95 (0.8, 1.09) | | d = 1.13 (0.98, 1.28) | | d = 0.8 (0.65, 0.94) | |
|  | ES = L | | ES = L | | ES = L | | ES = L | | ES = M to L | |
|  |  |  |  |  |  |  |  |  |  |  |
|  | **Financially coping** | | | | | | | | | |
|  | **EQ-HWB-9 UK pilot value set** | | | | | | | | | |
|  | **Australian dataset** | | **Canadian dataset** | | **New Zealand dataset** | | **United Kingdom dataset** | | **United States dataset** | |
| **Physical disability** |  |  |  |  |  |  |  |  |  |  |
| No | 576 | 0.809 (0.19) | 447 | 0.815 (0.17) | 582 | 0.833 (0.16) | 573 | 0.836 (0.16) | 513 | 0.818 (0.17) |
| Yes | 290 | 0.654 (0.24) | 233 | 0.664 (0.22) | 296 | 0.620 (0.27) | 227 | 0.630 (0.28) | 302 | 0.563 (0.31) |
|  | z = 10.8 (9.1, 12.4) | | z = 9.8 (8.1, 11.5) | | z = 12.8 (11.2, 14.4) | | z = 11.7 (10, 13.4) | | z = 13.5 (11.9, 15) | |
|  | d = 0.79 (0.65, 0.93) | | d = 0.81 (0.64, 0.98) | | d = 0.96 (0.81, 1.11) | | d = 0.91 (0.75, 1.06) | | d = 1.07 (0.91, 1.23) | |
|  | ES = M to L | | ES = M to L | | ES = L | | ES = M to L | | ES = L | |
| **Mental disability** |  |  |  |  |  |  |  |  |  |  |
| No | 644 | 0.812 (0.19) | 496 | 0.816 (0.17) | 622 | 0.843 (0.15) | 603 | 0.842 (0.16) | 565 | 0.821 (0.16) |
| Yes | 207 | 0.577 (0.25) | 181 | 0.609 (0.23) | 234 | 0.554 (0.26) | 186 | 0.571 (0.28) | 241 | 0.492 (0.31) |
|  | z = 13.1 (11.7, 14.6) | | z = 11.5 (9.9, 13.1) | | z = 15.9 (14.4, 17.3) | | z = 13.7 (12.2, 15.2) | | z = 15.5 (14.1, 16.9) | |
|  | d = 1.01 (0.87, 1.15) | | d = 0.98 (0.82, 1.15) | | d = 1.29 (1.13, 1.46) | | d = 1.12 (0.96, 1.28) | | d = 1.31 (1.14, 1.47) | |
|  | ES = L | | ES = L | | ES = L | | ES = L | | ES = L | |
| **Chronic conditions** |  |  |  |  |  |  |  |  |  |  |
| 0 to 1 health condition | 422 | 0.790 (0.22) | 301 | 0.817 (0.17) | 464 | 0.814 (0.20) | 454 | 0.839 (0.17) | 354 | 0.802 (0.22) |
| 2 or more health conditions | 466 | 0.722 (0.22) | 405 | 0.716 (0.22) | 438 | 0.709 (0.24) | 367 | 0.698 (0.26) | 481 | 0.663 (0.28) |
|  | z = 6.5 (4.6, 8.4) | | z = 6.9 (5, 8.8) | | z = 8.4 (6.5, 10.3) | | z = 9.2 (7.5, 11) | | z = 9 (7.1, 10.9) | |
|  | d = 0.45 (0.31, 0.59) | | d = 0.54 (0.38, 0.69) | | d = 0.58 (0.44, 0.73) | | d = 0.68 (0.53, 0.83) | | d = 0.66 (0.5, 0.81) | |
|  | ES = S to M | | ES = S to M | | ES = S to M | | ES = M to L | | ES = M to L | |
| **Sleep quality** |  |  |  |  |  |  |  |  |  |  |
| Good to very good | 438 | 0.765 (0.25) | 303 | 0.827 (0.17) | 437 | 0.771 (0.27) | 358 | 0.838 (0.20) | 425 | 0.745 (0.30) |
| Very poor to fair | 449 | 0.744 (0.20) | 402 | 0.708 (0.21) | 463 | 0.757 (0.18) | 463 | 0.728 (0.23) | 409 | 0.697 (0.22) |
|  | z = 4.3 (2.2, 6.3) | | z = 8.6 (6.8, 10.4) | | z = 5.5 (3.5, 7.5) | | z = 9.1 (7.3, 11) | | z = 6.2 (4.3, 8.1) | |
|  | d = 0.29 (0.15, 0.42) | | d = 0.62 (0.5, 0.74) | | d = 0.36 (0.23, 0.48) | | d = 0.61 (0.5, 0.72) | | d = 0.42 (0.3, 0.54) | |
|  | ES = Nil to S | | ES = M | | ES = S | | ES = S to M | | ES = S to M | |
| **Social support** |  |  |  |  |  |  |  |  |  |  |
| Good to excellent | 717 | 0.759 (0.23) | 548 | 0.783 (0.19) | 715 | 0.773 (0.24) | 624 | 0.804 (0.20) | 701 | 0.739 (0.26) |
| Poor to fair | 166 | 0.732 (0.21) | 154 | 0.673 (0.23) | 169 | 0.732 (0.18) | 195 | 0.684 (0.27) | 126 | 0.634 (0.24) |
|  | z = 2.7 (0.9, 4.4) | | z = 6.2 (4.5, 7.9) | | z = 5.1 (3.4, 6.7) | | z = 6.6 (4.8, 8.4) | | z = 5.6 (3.8, 7.3) | |
|  | d = 0.18 (0.06, 0.3) | | d = 0.46 (0.33, 0.58) | | d = 0.33 (0.23, 0.44) | | d = 0.45 (0.33, 0.57) | | d = 0.38 (0.27, 0.5) | |
|  | ES = Nil to S | | ES = S to M | | ES = S | | ES = S to M | | ES = S | |
| **Time for caring** |  |  |  |  |  |  |  |  |  |  |
| Always enough time | 510 | 0.766 (0.23) | 362 | 0.787 (0.20) | 439 | 0.752 (0.26) | 434 | 0.810 (0.21) | 446 | 0.709 (0.30) |
| Never/sometimes enough time | 375 | 0.740 (0.21) | 342 | 0.730 (0.21) | 451 | 0.777 (0.19) | 385 | 0.737 (0.23) | 388 | 0.736 (0.22) |
|  | z = 3.4 (1.4, 5.4) | | z = 4.4 (2.5, 6.3) | | z = 0.9 (1.1, 3) | | z = 5.9 (4, 7.8) | | z = 0.7 (1.3, 2.6) | |
|  | d = 0.23 (0.1, 0.36) | | d = 0.33 (0.19, 0.46) | | d = 0.06 (0.07, 0.2) | | d = 0.4 (0.28, 0.53) | | d = 0.05 (0.09, 0.18) | |
|  | ES = Nil to S | | ES = Nil to S | | ES = Nil | | ES = S to M | | ES = Nil | |
| **Impact of caring on own health** |  |  |  |  |  |  |  |  |  |  |
| No impact | 310 | 0.894 (0.12) | 230 | 0.863 (0.14) | 289 | 0.869 (0.15) | 275 | 0.877 (0.15) | 230 | 0.863 (0.16) |
| Impacts | 578 | 0.680 (0.23) | 476 | 0.709 (0.21) | 613 | 0.713 (0.24) | 546 | 0.725 (0.24) | 605 | 0.668 (0.27) |
|  | z = 15.7 (14.2, 17.1) | | z = 10.6 (8.9, 12.2) | | z = 11.7 (10.1, 13.4) | | z = 11.1 (9.4, 12.8) | | z = 12 (10.3, 13.7) | |
|  | d = 1.24 (1.08, 1.39) | | d = 0.87 (0.7, 1.03) | | d = 0.85 (0.71, 0.99) | | d = 0.84 (0.69, 1) | | d = 0.91 (0.75, 1.07) | |
|  | ES = L | | ES = M to L | | ES = M to L | | ES = M to L | | ES = M to L | |
| **Psychological wellbeing** |  |  |  |  |  |  |  |  |  |  |
| Good mental wellbeing | 542 | 0.803 (0.23) | 411 | 0.842 (0.16) |  | |  | | 548 | 0.760 (0.28) |
| Poor mental wellbeing | 346 | 0.678 (0.20) | 295 | 0.643 (0.21) |  |  |  |  | 287 | 0.648 (0.2) |
|  | z = 11.3 (9.6, 13) | | z = 14.1 (12.6, 15.6) | |  |  |  |  | z = 9.9 (8.2, 11.6) | |
|  | d = 0.82 (0.68, 0.97) | | d = 1.25 (1.06, 1.44) | |  |  |  |  | d = 0.73 (0.59, 0.87) | |
|  | ES = M to L | | ES = L | |  |  |  |  | ES = M to L | |
| **Self-reported quality of life** |  |  |  |  |  |  |  |  |  |  |
| Excellent/Very good | 506 | 0.785 (0.23) | 358 | 0.829 (0.17) | 574 | 0.803 (0.22) | 432 | 0.845 (0.18) | 508 | 0.761 (0.28) |
| Good | 297 | 0.762 (0.17) | 244 | 0.751 (0.17) | 240 | 0.731 (0.18) | 254 | 0.775 (0.18) | 245 | 0.694 (0.21) |
| Poor/Fair | 85 | 0.543 (0.23) | 103 | 0.537 (0.22) | 84 | 0.582 (0.26) | 133 | 0.552 (0.27) | 82 | 0.561 (0.22) |
|  | KW = 101 (68, 134) | | KW = 168 (131, 205) | | KW = 114 (77, 151) | | KW = 177 (138, 217) | | KW = 96 (64, 128) | |
|  | d = 0.71 (0.58, 0.84) | | d = 1.11 (0.95, 1.27) | | d = 0.76 (0.61, 0.9) | | d = 1.05 (0.9, 1.2) | | d = 0.71 (0.58, 0.85) | |
|  | ES = M to L | | ES = L | | ES = M to L | | ES = L | | ES = M to L | |
|  |  |  |  |  |  |  |  |  |  |  |
|  | **Financially strained** | | | | | | | | | |
|  | **EQ-HWB-9 UK pilot value set** | | | | | | | | | |
|  | **Australian dataset** | | **Canadian dataset** | | **New Zealand dataset** | | **United Kingdom dataset** | | **United States dataset** | |
| **Physical disability** |  |  |  |  |  |  |  |  |  |  |
| No | 283 | 0.702 (0.24) | 272 | 0.710 (0.20) | 271 | 0.756 (0.18) | 293 | 0.734 (0.20) | 202 | 0.691 (0.22) |
| Yes | 222 | 0.512 (0.28) | 212 | 0.504 (0.27) | 181 | 0.594 (0.25) | 168 | 0.455 (0.31) | 178 | 0.450 (0.28) |
|  | z = 7.9 (6.2, 9.7) | | z = 8.7 (7, 10.3) | | z = 7.2 (5.5, 9) | | z = 10 (8.4, 11.5) | | z = 8.4 (6.9, 10) | |
|  | d = 0.75 (0.56, 0.95) | | d = 0.86 (0.66, 1.05) | | d = 0.72 (0.53, 0.92) | | d = 1.05 (0.84, 1.26) | | d = 0.96 (0.74, 1.18) | |
|  | ES = M to L | | ES = M to L | | ES = M to L | | ES = L | | ES = M to L | |
| **Mental disability** |  |  |  |  |  |  |  |  |  |  |
| No | 254 | 0.747 (0.22) | 261 | 0.744 (0.19) | 273 | 0.786 (0.17) | 252 | 0.779 (0.18) | 188 | 0.723 (0.21) |
| Yes | 235 | 0.462 (0.27) | 214 | 0.470 (0.25) | 157 | 0.533 (0.24) | 197 | 0.443 (0.29) | 178 | 0.428 (0.27) |
|  | z = 12 (10.5, 13.4) | | z = 11.9 (10.6, 13.3) | | z = 11.1 (9.7, 12.5) | | z = 12.9 (11.6, 14.2) | | z = 10.3 (8.8, 11.7) | |
|  | d = 1.29 (1.07, 1.51) | | d = 1.31 (1.09, 1.52) | | d = 1.27 (1.04, 1.5) | | d = 1.54 (1.29, 1.78) | | d = 1.27 (1.02, 1.52) | |
|  | ES = L | | ES = L | | ES = L | | ES = L | | ES = L | |
| **Chronic conditions** |  |  |  |  |  |  |  |  |  |  |
| 0 to 1 health condition | 153 | 0.775 (0.20) | 165 | 0.740 (0.19) | 160 | 0.812 (0.15) | 186 | 0.776 (0.20) | 105 | 0.735 (0.23) |
| 2 or more health conditions | 378 | 0.548 (0.28) | 350 | 0.557 (0.26) | 312 | 0.621 (0.24) | 294 | 0.536 (0.28) | 289 | 0.514 (0.27) |
|  | z = 9.3 (7.7, 11) | | z = 7.8 (6.1, 9.6) | | z = 9.4 (7.7, 11) | | z = 10.1 (8.6, 11.7) | | z = 7.5 (5.8, 9.3) | |
|  | d = 0.89 (0.7, 1.07) | | d = 0.74 (0.55, 0.93) | | d = 0.96 (0.75, 1.16) | | d = 1.04 (0.84, 1.25) | | d = 0.82 (0.6, 1.04) | |
|  | ES = M to L | | ES = M to L | | ES = M to L | | ES = L | | ES = M to L | |
| **Sleep quality** |  |  |  |  |  |  |  |  |  |  |
| Good to very good | 118 | 0.733 (0.26) | 124 | 0.750 (0.19) | 96 | 0.811 (0.18) | 114 | 0.763 (0.26) | 87 | 0.684 (0.29) |
| Very poor to fair | 413 | 0.579 (0.27) | 388 | 0.576 (0.25) | 376 | 0.654 (0.23) | 366 | 0.587 (0.27) | 306 | 0.540 (0.27) |
|  | z = 6.4 (4.4, 8.3) | | z = 7 (5.2, 8.9) | | z = 7.2 (5.4, 9) | | z = 7.3 (5.4, 9.2) | | z = 5 (3, 7) | |
|  | d = 0.53 (0.38, 0.68) | | d = 0.59 (0.45, 0.74) | | d = 0.63 (0.48, 0.77) | | d = 0.63 (0.48, 0.78) | | d = 0.49 (0.31, 0.68) | |
|  | ES = S to M | | ES = S to M | | ES = S to M | | ES = S to M | | ES = S to M | |
| **Social support** |  |  |  |  |  |  |  |  |  |  |
| Good to excellent | 299 | 0.703 (0.24) | 284 | 0.697 (0.21) | 270 | 0.756 (0.20) | 263 | 0.680 (0.26) | 226 | 0.634 (0.27) |
| Poor to fair | 232 | 0.498 (0.28) | 224 | 0.522 (0.26) | 199 | 0.594 (0.24) | 214 | 0.567 (0.29) | 168 | 0.491 (0.27) |
|  | z = 8.8 (7.1, 10.5) | | z = 7.8 (6.1, 9.6) | | z = 7.9 (6.2, 9.7) | | z = 4.8 (3, 6.6) | | z = 5.4 (3.5, 7.2) | |
|  | d = 0.72 (0.6, 0.83) | | d = 0.66 (0.52, 0.79) | | d = 0.69 (0.56, 0.82) | | d = 0.43 (0.28, 0.58) | | d = 0.52 (0.36, 0.68) | |
|  | ES = M to L | | ES = M | | ES = M to L | | ES = S to M | | ES = S to M | |
| **Time for caring** |  |  |  |  |  |  |  |  |  |  |
| Always enough time | 243 | 0.653 (0.26) | 208 | 0.632 (0.25) | 204 | 0.721 (0.21) | 187 | 0.666 (0.28) | 173 | 0.601 (0.29) |
| Never/sometimes enough time | 285 | 0.579 (0.28) | 307 | 0.605 (0.25) | 266 | 0.657 (0.24) | 290 | 0.605 (0.28) | 220 | 0.550 (0.27) |
|  | z = 3.2 (1.3, 5.2) | | z = 1.3 (0.7, 3.2) | | z = 2.9 (1, 4.9) | | z = 3 (1.1, 4.9) | | z = 2 (0, 4) | |
|  | d = 0.28 (0.11, 0.45) | | d = 0.11 (0.06, 0.28) | | d = 0.27 (0.1, 0.44) | | d = 0.27 (0.1, 0.44) | | d = 0.2 (0, 0.4) | |
|  | ES = Nil to S | | ES = Nil to S | | ES = Nil to S | | ES = Nil to S | | ES = Nil to S | |
| **Impact of caring on own health** |  |  |  |  |  |  |  |  |  |  |
| No impact | 92 | 0.752 (0.24) | 88 | 0.732 (0.22) | 89 | 0.800 (0.18) | 89 | 0.761 (0.24) | 69 | 0.683 (0.29) |
| Impacts | 439 | 0.584 (0.28) | 427 | 0.592 (0.25) | 383 | 0.659 (0.23) | 391 | 0.599 (0.28) | 325 | 0.550 (0.27) |
|  | z = 6 (4, 8) | | z = 5.1 (3.3, 6.9) | | z = 5.8 (4, 7.6) | | z = 5.8 (3.9, 7.8) | | z = 4.2 (2.1, 6.3) | |
|  | d = 0.54 (0.35, 0.73) | | d = 0.46 (0.29, 0.63) | | d = 0.55 (0.36, 0.74) | | d = 0.55 (0.35, 0.75) | | d = 0.44 (0.21, 0.66) | |
|  | ES = S to M | | ES = S to M | | ES = S to M | | ES = S to M | | ES = S to M | |
| **Psychological wellbeing** |  |  |  |  |  |  |  |  |  |  |
| Good mental wellbeing | 173 | 0.792 (0.21) | 167 | 0.785 (0.17) |  | |  | | 123 | 0.756 (0.25) |
| Poor mental wellbeing | 358 | 0.527 (0.27) | 348 | 0.535 (0.25) |  |  |  |  | 271 | 0.490 (0.25) |
|  | z = 11.9 (10.3, 13.5) | | z = 11.5 (10.1, 12.8) | |  |  |  |  | z = 10.1 (8.5, 11.8) | |
|  | d = 1.2 (0.99, 1.42) | | d = 1.17 (0.98, 1.36) | |  |  |  |  | d = 1.19 (0.92, 1.45) | |
|  | ES = L | | ES = L | |  |  |  |  | ES = L | |
| **Self-reported quality of life** |  |  |  |  |  |  |  |  |  |  |
| Excellent/Very good | 120 | 0.793 (0.17) | 96 | 0.759 (0.19) | 121 | 0.836 (0.15) | 103 | 0.764 (0.25) | 87 | 0.752 (0.25) |
| Good | 212 | 0.685 (0.22) | 214 | 0.696 (0.19) | 185 | 0.729 (0.18) | 155 | 0.714 (0.18) | 143 | 0.603 (0.24) |
| Poor/Fair | 198 | 0.428 (0.28) | 204 | 0.464 (0.26) | 164 | 0.526 (0.23) | 219 | 0.502 (0.30) | 164 | 0.452 (0.27) |
|  | KW = 160 (122, 197) | | KW = 121 (86, 156) | | KW = 156 (120, 192) | | KW = 92 (59, 125) | | KW = 82 (53, 112) | |
|  | d = 1.31 (1.08, 1.53) | | d = 1.1 (0.89, 1.31) | | d = 1.4 (1.16, 1.65) | | d = 0.97 (0.75, 1.19) | | d = 1.02 (0.78, 1.25) | |
|  | ES = L | | ES = L | | ES = L | | ES = M to L | | ES = M to L | |
| All differences between groups were significant (p<0.001) | | | | | | | | | | |
| z: Mann-Whitney z-score; KW: Kruskal-Wallis H-statistic; d: Cohen’s d effect size | | | | | | | | | | |
| Cohen's d effect sizes: ≥0.2 small (S), ≥0.5 medium (M) and ≥0.8 large (L) | | | | | | | | | | |

**Appendix 15: Summary of psychometric *p*erformance *of the EQ-5D-5L and EQ-HWB-9* - subgroup analysis**

| **Over 60 (N=1,901)** | | | | | | |
| --- | --- | --- | --- | --- | --- | --- |
|  | **EQ-5D-5L Australia value set** | **EQ-5D-5L Canada value set** | **EQ-5D-5L New Zealand value set** | **EQ-5D-5L United Kingdom value set** | **EQ-5D-5L United States value set** | **EQ-HWB-9 UK pilot value set** |
| **Convergent validity ^a^** | ✓ | ✓ | ✓ | ✓ | ✓ | ✓ |
| **Known-group validity ^b^** | ✓ | ✓ | ✓ | ✓ | ✓ | ✓ |
|  | (9/9 groups) | (9/9 groups) | (9/9 groups) | (9/9 groups) | (9/9 groups) | (9/9 groups) |
| **EQ-5D-5L country value sets in country datasets** | | | | | | |
|  | **Australia dataset and value set** | **Canada dataset and value set** | **New Zealand dataset and value set** | **United Kingdom dataset and value set** | **United States dataset and value set** |  |
| **Convergent validity ^a^** | ✓ | ✓ | ✓ | ✓ | ✓ |  |
| **Known-group validity ^b^** | ✓ | ✓ | ✓ | ✓ | ✓ |  |
|  | (8/9 groups) | (9/9 groups) | (8/8 groups) | (7/8 groups) | (9/9 groups) |  |
| **EQ-HWB-9 UK pilot in country datasets** | | | | | | |
|  | **Australia dataset** | **Canada dataset** | **New Zealand dataset** | **United Kingdom dataset** | **United States dataset** |  |
| **Convergent validity ^a^** | ✓ | ✓ | ✓ | ✓ | ✓ |  |
| **Known-group validity ^b^** | ✓ | ✓ | ✓ | ✓ | ✓ |  |
|  | (9/9 groups) | (9/9 groups) | (8/8 groups) | (8/8 groups) | (9/9 groups) |  |
|  | | | | | | |
| **Under 60 (N=4,842)** | | | | | | |
|  | **EQ-5D-5L Australia value set** | **EQ-5D-5L Canada value set** | **EQ-5D-5L New Zealand value set** | **EQ-5D-5L United Kingdom value set** | **EQ-5D-5L United States value set** | **EQ-HWB-9 UK pilot value set** |
| **Convergent validity ^a^** | ✓ | ✓ | ✓ | ✓ | ✓ | ✓ |
| **Known-group validity ^b^** | ✓ | ✓ | ✓ | ✓ | ✓ | ✓ |
|  | (8/9 groups) | (8/9 groups) | (8/9 groups) | (8/9 groups) | (8/9 groups) | (8/9 groups) |
| **EQ-5D-5L country value sets in country datasets** | | | | | | |
|  | **Australia dataset and value set** | **Canada dataset and value set** | **New Zealand dataset and value set** | **United Kingdom dataset and value set** | **United States dataset and value set** |  |
| **Convergent validity ^a^** | ✓ | ✓ | ✓ | ✓ | ✓ |  |
| **Known-group validity ^b^** | ✓ | ✓ | ✓ | ✓ | ✓ |  |
|  | (9/9 groups) | (8/9 groups) | (7/8 groups) | (8/8 groups) | (8/9 groups) |  |
| **EQ-HWB-9 UK pilot in country datasets** | | | | | | |
|  | **Australia dataset** | **Canada dataset** | **New Zealand dataset** | **United Kingdom dataset** | **United States dataset** |  |
| **Convergent validity ^a^** | ✓ | ✓ | ✓ | ✓ | ✓ |  |
| **Known-group validity ^b^** | ✓ | ✓ | ✓ | ✓ | ✓ |  |
|  | (8/9 groups) | (8/9 groups) | (7/8 groups) | (8/8 groups) | (8/9 groups) |  |
|  | | | | | | |
| **Coping financially (N=4,259)** | | | | | | |
|  | **EQ-5D-5L Australia value set** | **EQ-5D-5L Canada value set** | **EQ-5D-5L New Zealand value set** | **EQ-5D-5L United Kingdom value set** | **EQ-5D-5L United States value set** | **EQ-HWB-9 UK pilot value set** |
| **Convergent validity ^a^** | ± | ± | ✓ | ± | ± | ✓ |
| **Known-group validity ^b^** | ✓ | ✓ | ✓ | ✓ | ✓ | ✓ |
|  | (8/9 groups) | (8/9 groups) | (8/9 groups) | (8/9 groups) | (8/9 groups) | (9/9 groups) |
| **EQ-5D-5L country value sets in country datasets** | | | | | | |
|  | **Australia dataset and value set** | **Canada dataset and value set** | **New Zealand dataset and value set** | **United Kingdom dataset and value set** | **United States dataset and value set** |  |
| **Convergent validity ^a^** | ± | ✓ | ± | ✓ | ± |  |
| **Known-group validity ^b^** | ✓ | ✓ | ✓ | ✓ | ✓ |  |
|  | (8/9 groups) | (9/9 groups) | (7/8 groups) | (8/8 groups) | (8/9 groups) |  |
| **EQ-HWB-9 UK pilot in country datasets** | | | | | | |
|  | **Australia dataset** | **Canada dataset** | **New Zealand dataset** | **United Kingdom dataset** | **United States dataset** |  |
| **Convergent validity ^a^** | ✓ | ✓ | ✓ | ✓ | ✓ |  |
| **Known-group validity ^b^** | ✓ | ✓ | ✓ | ✓ | ✓ |  |
|  | (8/9 groups) | (9/9 groups) | (7/8 groups) | (8/8 groups) | (8/9 groups) |  |
|  | | | | | | |
| **Financially strained (N=2,458)** | | | | | | |
|  | **EQ-5D-5L Australia value set** | **EQ-5D-5L Canada value set** | **EQ-5D-5L New Zealand value set** | **EQ-5D-5L United Kingdom value set** | **EQ-5D-5L United States value set** | **EQ-HWB-9 UK pilot value set** |
| **Convergent validity ^a^** | ✓ | ✓ | ✓ | ✓ | ✓ | ✓ |
| **Known-group validity ^b^** | ✓ | ✓ | ✓ | ✓ | ✓ | ✓ |
|  | (8/9 groups) | (8/9 groups) | (8/9 groups) | (8/9 groups) | (8/9 groups) | (9/9 groups) |
| **EQ-5D-5L country value sets in country datasets** | | | | | | |
|  | **Australia dataset and value set** | **Canada dataset and value set** | **New Zealand dataset and value set** | **United Kingdom dataset and value set** | **United States dataset and value set** |  |
| **Convergent validity ^a^** | ± | ✓ | ✓ | ✓ | ✓ |  |
| **Known-group validity ^b^** | ✓ | ✓ | ✓ | ✓ | ✓ |  |
|  | (8/9 groups) | (8/9 groups) | (7/8 groups) | (8/8 groups) | (8/9 groups) |  |
| **EQ-HWB-9 UK pilot in country datasets** | | | | | | |
|  | **Australia dataset** | **Canada dataset** | **New Zealand dataset** | **United Kingdom dataset** | **United States dataset** |  |
| **Convergent validity ^a^** | ✓ | ✓ | ✓ | ✓ | ✓ |  |
| **Known-group validity ^b^** | ✓ | ✓ | ✓ | ✓ | ✓ |  |
|  | (9/9 groups) | (8/9 groups) | (8/8 groups) | (8/8 groups) | (9/9 groups) |  |
| ^a^ Convergent validity: ✓ strong correlations (≥0.60) across majority of correlation coefficients; ± moderate correlations across majority of correlation coefficients (0.40–0.59). ^b^ Known-group validity: ✓ at least small effect sizes (≥0.20) for at least 75% groups; otherwise ±. | | | | | | |
